# Automated Ischemic Lesion Segmentation in MRI Mouse Brain Data after Transient Middle Cerebral Artery Occlusion

**DOI:** 10.3389/fninf.2017.00003

**Published:** 2017-01-31

**Authors:** Inge A. Mulder, Artem Khmelinskii, Oleh Dzyubachyk, Sebastiaan de Jong, Nathalie Rieff, Marieke J. H. Wermer, Mathias Hoehn, Boudewijn P. F. Lelieveldt, Arn M. J. M. van den Maagdenberg

**Affiliations:** ^1^Department of Neurology, Leiden University Medical CenterLeiden, Netherlands; ^2^Division of Image Processing (LKEB), Department of Radiology, Leiden University Medical CenterLeiden, Netherlands; ^3^Percuros B.V.Enschede, Netherlands; ^4^Department of Human Genetics, Leiden University Medical CenterLeiden, Netherlands; ^5^In-vivo-NMR Laboratory, Max Planck Institute for Metabolism ResearchCologne, Germany; ^6^Intelligent Systems Group, Faculty of Electrical Engineering, Mathematics and Computer Science, Delft University of TechnologyDelft, Netherlands

**Keywords:** automated segmentation, quantification, ischemic stroke, MRI, lesion, volume, mouse

## Abstract

Magnetic resonance imaging (MRI) has become increasingly important in ischemic stroke experiments in mice, especially because it enables longitudinal studies. Still, quantitative analysis of MRI data remains challenging mainly because segmentation of mouse brain lesions in MRI data heavily relies on time-consuming manual tracing and thresholding techniques. Therefore, in the present study, a fully automated approach was developed to analyze longitudinal MRI data for quantification of ischemic lesion volume progression in the mouse brain. We present a level-set-based lesion segmentation algorithm that is built using a minimal set of assumptions and requires only one MRI sequence (T2) as input. To validate our algorithm we used a heterogeneous data set consisting of 121 mouse brain scans of various age groups and time points after infarct induction and obtained using different MRI hardware and acquisition parameters. We evaluated the volumetric accuracy and regional overlap of ischemic lesions segmented by our automated method against the ground truth obtained in a semi-automated fashion that includes a highly time-consuming manual correction step. Our method shows good agreement with human observations and is accurate on heterogeneous data, whilst requiring much shorter average execution time. The algorithm developed here was compiled into a toolbox and made publically available, as well as all the data sets.

## Introduction

In pre-clinical research on ischemic stroke, histological evaluation of brain infarct volume is accepted as the gold standard. However, errors introduced due to changes in brain morphology during processing of brain sections (swelling/shrinkage of tissue) combined with its manual-labor-intensive nature make it a suboptimal evaluation method. Moreover, animal sacrifice makes longitudinal studies and multiple readout times impossible, or, alternatively, will considerably increase the number of animals used in case parallel groups are investigated. Hence, magnetic resonance imaging (MRI) has become increasingly important to assess infarct volume development in animal experiments. With MRI, ischemic stroke can be analyzed in the first few days after induction using spin-spin relaxation time contrast T2 (Jacobs et al., 2000), which is sensitive to vasogenic edema (Dijkhuizen and Nicolay, 2003). Using MRI, infarct characteristics such as infarct volume and progression can be examined in a longitudinal manner (Hoehn-Berlage et al., 1995a,b; Weber et al., 2006). Efficient use of computational image processing methods allows for automated reproducible quantitative analysis of such data.

In clinical research, several algorithms for detection, segmentation and classification of different brain abnormalities (brain tumors, trauma lesions, hematoma, edemas, Alzheimer's disease) in both MRI and CT have been developed (Ghosh et al., 2014). However, segmentation of brain lesion in MRI animal (especially mouse) imaging data still heavily relies on manual tracing and semi-automated thresholding techniques (Rekik et al., 2012; Leithner et al., 2015; Yao et al., 2015; Yin et al., 2015; Zheng et al., 2015; Donath et al., 2016; Moraga et al., 2016; Yip et al., 2016), making the analysis time-consuming, with low inter- and intra-observer reproducibility (Niimi et al., 2007; Ghosh et al., 2011; Rekik et al., 2012). Also, there is considerable variability between research centers with respect to the methods used to calculate lesion volume using MRI (Milidonis et al., 2015), which can result in unjustified conclusions.

When it comes to ischemic brain tissue segmentation of pre-clinical data, only a few (semi-) automated algorithms have been developed. These algorithms typically use data from multiparametric MR imaging, more specifically by combining the apparent diffusion coefficient (ADC) maps with T1, T2, T1- and T2-weighted images to distinguish and characterize healthy and ischemic brain tissues (Hoehn-Berlage et al., 1995a; Li et al., 2002; Sotak, 2002; van Dorsten et al., 2002; Wang et al., 2007). Ghosh et al. (2011, 2012, 2014) developed an automated method—Hierarchical Region Splitting (HRS)—where adaptive thresholds are automatically selected to detect, quantify and distinguish between core and penumbral tissue regions in T2-weighted MRI data of HII Sprague-Dawley rats. Jacobs et al. (1999a,b, 2000, 2001a,b) developed an unsupervised segmentation algorithm based on K-means clustering—iterative self-organizing data analysis technique (ISODATA)—for analysis of multiparametric MRI data of focal cerebral ischemia in Wistar rats that was validated with histology data. Ding et al. (2004, 2006) applied a modified version of the same method to analyze embolic stroke rat data. A comprehensive overview of all available image analysis methods for ischemic stroke lesion and a discussion of their pros and cons was provided by Rekik et al. (2012).

Most of the aforementioned methods were developed for rats, are age- and disease-specific and require manual intervention. The only two automated segmentation methods developed for pre-clinical data—HRS (Ghosh et al., 2011, 2012, 2014) and ISODATA (Jacobs et al., 1999a,b, 2000, 2001a,b)—obtained promising results but specifically focused on neonatal and adult rat and were validated on relatively small data sets.

Moreover, current pre-clinical stroke studies are compromised by reproducibility issues (Dirnagl, 2016; Llovera and Liesz, 2016). Having a reproducible automated method available might be of particular value when considering published guidelines for reporting animal research studies (Kilkenny et al., 2010). To the best of our knowledge, there are no automated methods that have been developed to segment ischemic lesions in MRI mouse brain data.

Thus, the goal of this study was to develop an automated approach to quantify ischemic lesion volumes in MRI data of mouse brains and to make it publicly available. Our algorithm is built upon existing segmentation paradigm (level sets) with a minimal set of assumptions and requires only one MRI sequence (T2) as input. We validated our algorithm on heterogeneous data consisting of 121 mouse brain scans, that covered various age groups, time points after infarct induction and were obtained with different MRI hardware and acquisition parameters. Performance of our automated approach was compared against the ground truth obtained in a semi-automated fashion by two observers, evaluating the volumetric accuracy and regional overlap of segmented ischemic lesions. Our approach showed good agreement with results obtained by the human observers and high accuracy on heterogeneous data, whilst having much shorter execution times. Thus, it has considerable potential in replacing the biased manual labor in quantifying ischemic lesion volume in mouse brain MRI data and might be of value for MRI stroke volume analysis in a multicenter setting.

## Materials and methods

### Animals

Our computational approach was tested on three main groups of male mice that were labeled as “*Leiden-Set,” “Cologne-Set-1”* and “*Cologne-Set-2”* depending on the origin of the data and data acquisition protocol.

*Leiden-Set*: C57BL/6J mice (*n* = 65) were further subdivided into three age groups (3- to 5-, 12- to 14- and 20- to 24-month-old). Mice were repeatedly scanned at different time points: 4 h, 24 h, 48 h and 8 d after infarct induction (see Section “Experimental Infarct Model”). Histological specimens were obtained on a small subset of animals (*n* = 6) and used for visual validation of the infarct area.
*Cologne-Set-1*: C57BL/6J mice (*n* = 6; 3-month-old) were scanned at 18 h and 4 d after infarct induction.
*Cologne-Set-2*: Transgenic mice expressing luciferase under doublecortin control (DCX-Luc, Couillard-Despres et al., 2008) (*n* = 10; 2- and 12-month-old) were scanned at 48 h after infarct induction.

The group of 3- to 5-month-old mice from the *Leiden-Set*, 24 h after stroke induction, was used as a primary validation cohort as it was the largest group (Table 1) and because the 24-h time point is often chosen in cerebral infarction experiments. For better assessment of the performance of our method on different infarct shapes and volumes, we subdivided this cohort into striatal (Str) and corticostriatal (Ctx+Str) lesions.

**Table 1:** **Overview of all the used mice and accompanying MRI data sets: origin/acquisition protocol, number, age, time points after experimental infarct induction**.

	**Data sets**													
	***Leiden-Set*** **C57BL/6J**										***Cologne-Set-1*** **C57BL/6J**		***Cologne-Set-2*** **DCX-Luc**	
Age (months)	3–5				12–14			20–24			3		2	12
Infarct induction	4 h	24 h	48 h	8 d	4 h	24 h	48 h	4 h	24 h	48 h	18 h	4 d	48 h	
*N*	11	36	8	6	7	9	8	5	7	5	6	3	5	5
**MRI ACQUISITION PARAMETERS**														
Magnet strength (T)	7										11.7		11.7	
Repetition time (ms)	4000										5000		4500	
Echo time (ms)	9										10.25		10	
Number of echoes	20										16		16	
Number of averages	2										1		1	
Field of view (mm^2^)	15 × 15										14 × 14		20 × 20	
Matrix	128 × 128										128 × 128		196 × 196	
Number of slices	16										10		10	12
Slice thickness (mm)	0.50										0.80		0.60	
Voxel size (mm^3^)	0.12 × 0.12 × 0.50										0.11 × 0.11 × 0.80		0.10 × 0.10 × 0.60	
Inter-slice gap (mm)	no gap										no gap		no gap	
Bandwidth (Hz)	59,523.8										50,000		75,000	
Acquisition time	12 min 48 s										10 min 40 s		9 min 18 s	

Animals were housed with littermates, in a temperature-controlled environment, with food and water *ad libitum*. All animal experiments performed at the Leiden University Medical Center (LUMC) were approved by the local committee for animal health, ethics and research of LUMC. All animal experiments conducted at the Max Plank Institute for Metabolism Research in Cologne were performed in accordance with the German Animal Welfare Act and approved by the local authorities (Landesamt für Naturschutz, Umwelt und Verbraucherschutz NRW).

### Experimental infarct model

Infarcts were induced using a modified transient middle cerebral artery occlusion (*t*MCAo) model first described by Longa et al. (1989). Mice were anesthetized using isoflurane (3% induction, 1.5% maintenance) in 70% pressurized air and 30% O_2_. Painkiller carprofen (5 mg/kg, s.c.; Carporal, 50 mg/mL, AST Farma BV, Oudewater, the Netherlands) was given before surgery. During surgery, the mouse body temperature was maintained at 37°C using a rectal probe and feedback system. During the surgical procedure, a silicone-coated nylon monofilament (7017PK5Re; Doccol Company, Redlands, CA, USA) was inserted into the right common carotid artery and advanced via the internal carotid artery and circle of Willis to eventually block the middle cerebral artery (MCA) at its origin (decreasing blood flow substantially in the MCA territory, in the right hemisphere) and the skin was sutured. During the occlusion period, the mouse was allowed to wake up in a temperature-controlled incubator (V1200; Peco Services Ltd, Brough, UK). After 30 min of occlusion, the mouse was re-anesthetized in order to remove the suture and withdraw the monofilament to allow reperfusion. After surgery, the animal was allowed to recover for 2 h in the incubator to maintain body temperature at 37°C, with easy access to food and water. At the end of the experiment, brains were fixated by transcardially perfusing the mice using fresh cold 4% buffered PFA (Paraformaldehyde P6148; Sigma-Aldrich Co. LLC, Saint Louis, MO, USA). Brains were collected, processed and sectioned. Sections were stained with Nissl (Cresyl Violet, Merck Millipore, Billerica, MA, USA) using standard protocol.

### Magnetic resonance imaging

Scans were acquired with small-animal Bruker MRI systems using a Multi-Slice Multi-Echo sequence protocol at different time points after infarct induction. Animals from the *Leiden-Set* were scanned at 7 T (Pharmascan, Bruker BioSpin, Ettlingen, Germany), whilst animals from the *Cologne-Sets* were scanned at 11.7 T (Biospec 11.7 T/16, Bruker BioSpin). Quantitative T2 maps were calculated from the multi-echo trains using Paravision 5.1 software (Bruker Pharmascan) for the *Leiden-Set* and IDL software for the *Cologne-Sets*. Table 1 shows a complete overview of all 121 scans, together with a summary of the main imaging acquisition parameters.

### Ischemic lesion segmentation challenges

The ischemic lesion is characterized by elevated T2 values with respect to the healthy brain tissue. However, it is not possible to segment the ischemic lesion with a simple threshold as several other objects, like ventricles, structures surrounding the brain and other regions in the periventricular zone of the brain, share similar T2 values to that of the infarct lesion. Also, both the ventricles and the stroke regions vary considerably in shape and size between different subjects and different time points after infarct induction, and in many cases the stroke region engulfs the ventricles making the segmentation task even more difficult. Manual delineation of the ischemic lesion by experts takes into account all this information, together with possible stroke density differences and contiguity of the lesion area throughout the MRI image stack.

### Semi-automated ischemic lesion segmentation

**Figure 1:**
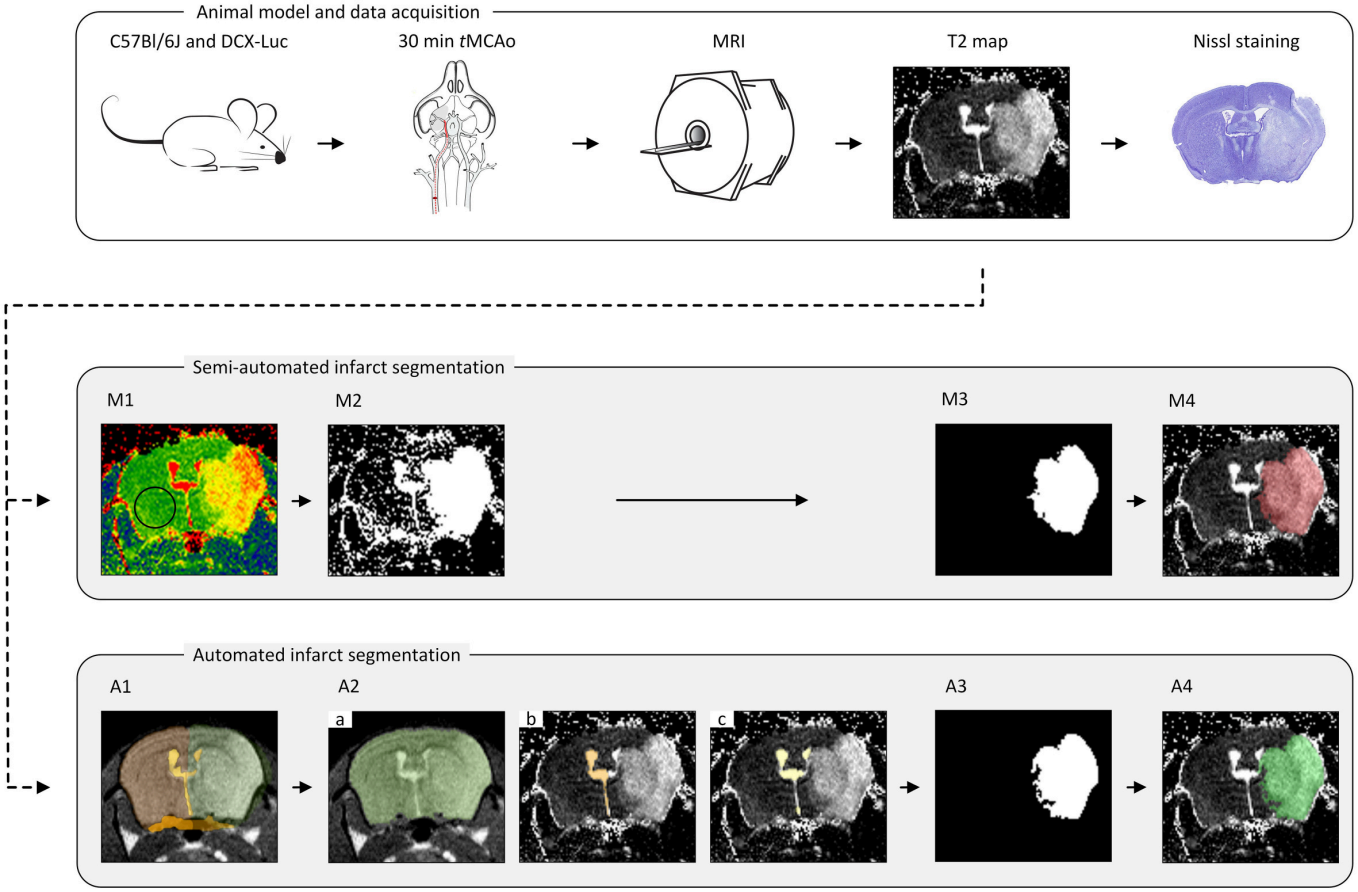
**The pipeline for the animal model, data acquisition and lesion segmentation**. The transient middle cerebral artery occlusion (*t*MCAo) model was used for infarct induction in mice of different age groups. A Multi-Slice Multi-Echo MRI scan was acquired for determination of quantitative T2 maps at multiple time points (*T* = 4 h, 18 h, 24 h, 48 h, 4 d and 8 d) after *t*MCAo. A subset of infarcted brains was used for histological validation. All MRI scans were analyzed in a semi-automated fashion by two observers (IM and SdJ) using threshold determination **(M1)**, threshold mask **(M2)**, manual exclusion of non-infarct-related areas **(M3)** and lesion volume determination **(M4)**. Automated lesion segmentation was performed using image registration **(A1)**, brain segmentation **(A2.a)**, contralateral ventricle segmentation **(A2.b)**, ventricles segmentation **(A2.c)**, infarct ROI segmentation **(A3)** and infarct ROI volume quantification **(A4)**.

Semi-automated segmentation was performed in a slice-by-slice manner by two trained observers (IM and SdJ) using freely available ImageJ software (https://imagej.nih.gov/ij/); see Figure 1.

M1. *Threshold determination*. The threshold, the same for both observers, was determined as the mean plus two standard deviations of a vector containing average T2 values within a ROI in the contralateral hemisphere for every group of animals (concerning time point, age and data set origin) (Figure 1M1).
M2. *Threshold mask*. A mask was compiled of all high-intensity pixels above the threshold value (Figure 1M2).
M3. *Infarct ROI segmentation*. Confounding regions (ventricles and other non-infarct-related high-intensity areas) were manually removed by both observers individually (thereby highly relying on anatomical knowledge, left-right symmetry, density and experience), resulting in the delineated infarct lesion (Figure 1M3).
M4. *Infarct ROI volume quantification*. For each observer, the number of voxels located inside the ROIs representing the infarcted area of each MRI slice was multiplied by the voxel size to obtain the total infarct lesion volume (Figure 1M4).

### Automated ischemic lesion segmentation

The infarct lesion was segmented on the T2 map based on its elevated T2 values with respect to the healthy brain tissue. However, as mentioned above, several other objects appearing on the T2 map share similar T2 values to that of the infarct lesion. To discriminate the infarcted area from other brain structures and to quantify the infarct lesion volume, we developed a fully automated approach that integrates a series of image processing steps depicted in Figure 1.

Our approach is based on two general assumptions that: (i) T2 value distribution of the infarct lesion differs from that of the healthy tissue; and (ii) only the ipsilateral hemisphere is affected by the infarct, whereas the contralateral hemisphere remains unaffected. Thus, we use the latter to learn the T2 value distributions of the ventricles and of the healthy brain tissue and apply this information to segment the infarcted area. All segmentations were performed on 3D image volumes using a modification of the region-based level sets method of Chan and Vese (2001). The parameters of our method (listed in Table 2) were optimized on the *Leiden-Set* and fixed for all three data sets during validation. The remainder of this section provides a detailed description of each particular step.

**Table 2:** **Parameters of the level-set-based segmentation algorithm for different segmentation steps**.

	**Whole brain**	**Contralateral ventricle**	**Ventricles**	**Stroke**
*I*(**x**)	4th echo	T2 map	T2 map	T2 map
*P*(*I*(**x**); Ω _*O*_)	Gaussian	Gaussian	Histogram; fixed	Histogram
*P*(*I*(**x**); Ω _*B*_)	Gaussian	Gaussian	Histogram	Histogram; fixed
Ω	ℝ^3^	*R*_*CBH*_	*R*_*WB*_	*R*_*IBH*_\(*R*_*V*_ ∪ *M*_*PVZ*_)
ϕ_0_(**x**)	*M*_*WB*_	*M*_*CV*_	*M*_*V*_	$R_{s}^{init}$
α	5	0.1	2	0.3
μ	2	0	1.5	0
*n*_*iter*_	80	120	120	80
*g*(**x**)	−	−	+	−

#### Image registration

In this step, each brain scan was registered to a template brain consisting of a number of manually drawn labels: whole-brain (*M*_*WB*_), ipsilateral hemisphere (*M*_*IBH*_), contralateral hemisphere (*M*_*CBH*_), ipsilateral ventricle (*M*_*IV*_), contralateral ventricle (*M*_*CV*_) and periventricular zone (*M*_*PVZ*_); see Figure 1A1. The template labels were propagated to each subject and used to initialize the subsequent steps. In the following, by referring to a region being occupied by a certain label we mean the result of the label propagation.

For each subject, the sum of all its echo images was used to register the scan of that particular subject to the reference brain scan. Consequently, the template labels were propagated to the individual data sets using the information provided by the deformation field for each subject-to-reference registration. The labels were used to initialize the segmentation of the whole brain and the ventricles as described in the next section. The quality and success of the registration was visually inspected by three independent observers (AK, OD and IM).

Registration was performed in a coarse-to-fine fashion. Initially, rigid registration was performed to compensate for translation and rotation. Afterwards, affine registration was conducted to compensate for differences in brain size. Because large deformations occur in stroke brains, a non-rigid B-spline registration was necessary to compensate for the large local changes (especially in the ipsilateral hemisphere and the ventricles region). A Gaussian image pyramid was employed in all registration steps, applying four resolutions for the rigid and two for the affine and B-spline registrations each. Normalized Correlation Coefficient was used as a similarity metric.

#### Segmentation

For each particular segmentation, we used a level set function ϕ(**x**) to partition the image space Ω into two classes, further referred to as “object” (Ω_*O*_ = {**x** ∈ Ω : ϕ(**x**) ≥ 0}) and “background” (Ω_*B*_ = {**x** ∈ Ω : ϕ(**x**) < 0}), respectively. Here **x** ∈ Ω ⊂ ℝ^3^ are the Cartesian coordinates. The level set function ϕ(**x**) was evolved from its initial state ϕ_0_(**x**) for the predefined number of iterations *n*_*iter*_ or till the stopping criterion was reached. The energy functional *E*(ϕ) contains both image-based and regularization-based terms and, in its most general form, is given by the following equation (Rousson and Deriche, 2002):

$$\begin{array}{l} E\left(\phi \right)=\alpha \cdot g\left(\text{x}\right)\cdot \text{Length}\left(\partial \Omega_{O}\right)+\mu \cdot \text{Area}\left(\Omega_{O}\right)\\ -\log P\left(I\left(\text{x}\right);\Omega_{O}\right)-\log P\left(I\left(\text{x}\right);\Omega_{B}\right). \end{array}$$

Here *g*(**x**) is the gradient map (Dufour et al., 2005) that can be optionally used to drive the segmentation toward the boundaries of the structures of interest, and α and μ are weights. Parameter values for different segmentation steps described in the remainder of this section are summarized in Table 2.

##### Data preprocessing

After the label propagation was completed, each scan was cropped to the rectangle containing the volume of interest. The cropping rectangle was defined as the smallest rectangle that contains the brain mask *M*_*WB*_ obtained as result of the label propagation. The volume intensities were consequently scaled to the [0;1] range. For the echo images we performed an additional per-slice intensity normalization by mapping the cumulative intensity distribution of each slice to that of the chosen reference slice (the one with the maximum entropy) and rescaling the image intensity accordingly.

##### Whole brain segmentation

In this step, the segmentation was performed by minimizing the energy functional *E*(ϕ) on the fourth echo image; see Figure 1A2.a. The level set function in this case was initialized by the whole-brain mask *M*_*WB*_ obtained as result of label propagation. The final result, denoted as *R*_*WB*_, was obtained by: (1) Applying the morphological hole filling and morphological opening with a disk of radius of two pixels as the structure element on the result of the level set propagation; and (2) Selecting the largest connected component. The parameter values for this step are provided in Table 2.

##### Contralateral ventricle segmentation

Contralateral ventricle *R*_*CV*_ was segmented from the T2 map by running the level sets starting from the result of the label propagation; see Figure 1A2.b. In this case, the level set evolution was restricted to the contralateral hemisphere *R*_*CBH*_ = *R*_*WB*_\*M*_*IBH*_, the rest of the parameter values are listed in Table 2.

##### Ventricles segmentation

Ventricles *R*_*V*_ were segmented on the T2 map by evolving the level sets inside *R*_*WB*_; see Figure 1A2.c. The object (ventricle) energy was calculated from the segmented contralateral ventricle and kept fixed. To prevent the segmentation from leaking into the stroke area touching the ventricles, we used the gradient map that was defined as *g*$\left(\text{x}\right)=1-e_{v}^{2}\left(\text{x}\right)$, where *e*_*v*_(**x**) = −log *P*_*Gaussian*_(*I*(**x**); *R*_*CV*_) is the energy (assuming the Gaussian intensity distribution) scaled to the [0;1] interval, and parameters of the Gaussian distribution were calculated from *R*_*CV*_ (the region occupied by the contralateral ventricle).

##### Stroke segmentation

Finally, the stroke area was segmented from the T2 map; see Figure 1A3. To initialize the level set function, we initially calculated the area where the histogram-based energy of the brain region *R*_*WB*_ with the ventricles and the periventricular area excluded (*R*_*WB*_\(*R*_*V*_ ∪ *M*_*PVZ*_)) was larger than that of the contralateral hemisphere (*R*_*CBH*_\(*R*_*V*_ ∪ *M*_*PVZ*_)):

$$\begin{array}{l} R_{s}^{init}=P\left(I\left(\text{x}\right);R_{B}\backslash \left(R_{V}\cup M_{PVZ}\right)\right)\\ >P\left(I\left(\text{x}\right);R_{CBH}\backslash \left(R_{V}\cup M_{PVZ}\right)\right). \end{array}$$

Consequently, connected components that did not intersect with dense areas on $R_{s}^{init}$ were filtered out. The dense areas were defined as binary masks composed of all voxels in $R_{s}^{init}$ for which at least 75% of their neighbors (the neighborhood was in this case defined as the circle of radius 4 around the voxel of interest) belong to $R_{s}^{init}$. In this case, evolution of the level set function was restricted to *R*_*IBH*_\(*R*_*V*_ ∪ *M*_*PVZ*_) (the ipsilateral hemisphere without the ventricles and the periventricular area). The energy of the background was assumed fixed and equal to that calculated from the contralateral hemisphere *R*_*CBH*_. The rest of the parameters are provided in Table 2.

##### Parameter selection

The parameters (α, μ, *n*_*iter*_) for the level-set-based segmentation were optimized on the *Leiden-Set*. Suitable parameter values for segmenting the whole brain region, the contralateral ventricle and both ventricles were determined by trial and error. The parameters for the stroke segmentation were determined by maximizing the Dice index (Dice, 1945) via exhaustive search within an empirically selected range of feasible values for each parameter. The final values used for validation of our method, the same for all three data sets, are reported in Table 2.

#### Implementation

All the data are publically available and published as a data report (Mulder et al., in review). The template labels were manually drawn based on the Allen Brain Atlas (Sunkin et al., 2013; http://www.brain-map.org/) using AMIRA (v5, FEI Software, Hillsboro, OR, USA). Both registration and segmentation were implemented in MATLAB R2012b (The MathWorks, Inc., Natick, MA, USA) and compiled into a toolbox that can be downloaded from the following webpage (www.lkeb.nl). The registration scheme was implemented using the open-source image registration toolbox elastix (v4.700; Klein et al., 2010). Information on the used registration parameters can be found on the elastix website (http://elastix.bigr.nl/wiki/index.php/Par0038). Average computational time for executing the entire segmentation routine on one data set on 3.60 GHz Intel(R) Xeon(R) computer with 32 GB RAM was: 4 s for segmentation and 309 s for prior registration and label propagation.

### Performance measures and statistical analysis

Ischemic lesion regions segmented by the observers and the proposed automated approach were compared using two primary measures: total volume of the ischemic lesion and Dice index (Dice, 1945) that measures overlap between two regions *R*_1_ and *R*_2_:

$$\begin{array}{l} Dice\;index\;\left(R_{1},R_{2}\right)\;=\;2\frac{\left|R_{1\;}\cap \;R_{2}\right|}{\left|R_{1}|+|R_{2}\right|}. \end{array}$$

Intraclass Correlation Coefficient (ICC) and the accompanying trend line were also calculated for each group of interest. ICC (2-way mixed with absolute agreement) was determined with SPSS (SPSS Statistics 23; IBM Corp., Armonk, NY, USA) to investigate the correlation between the mean of the two observers and the calculated automated volumes.

## Results

**Figure 2:**
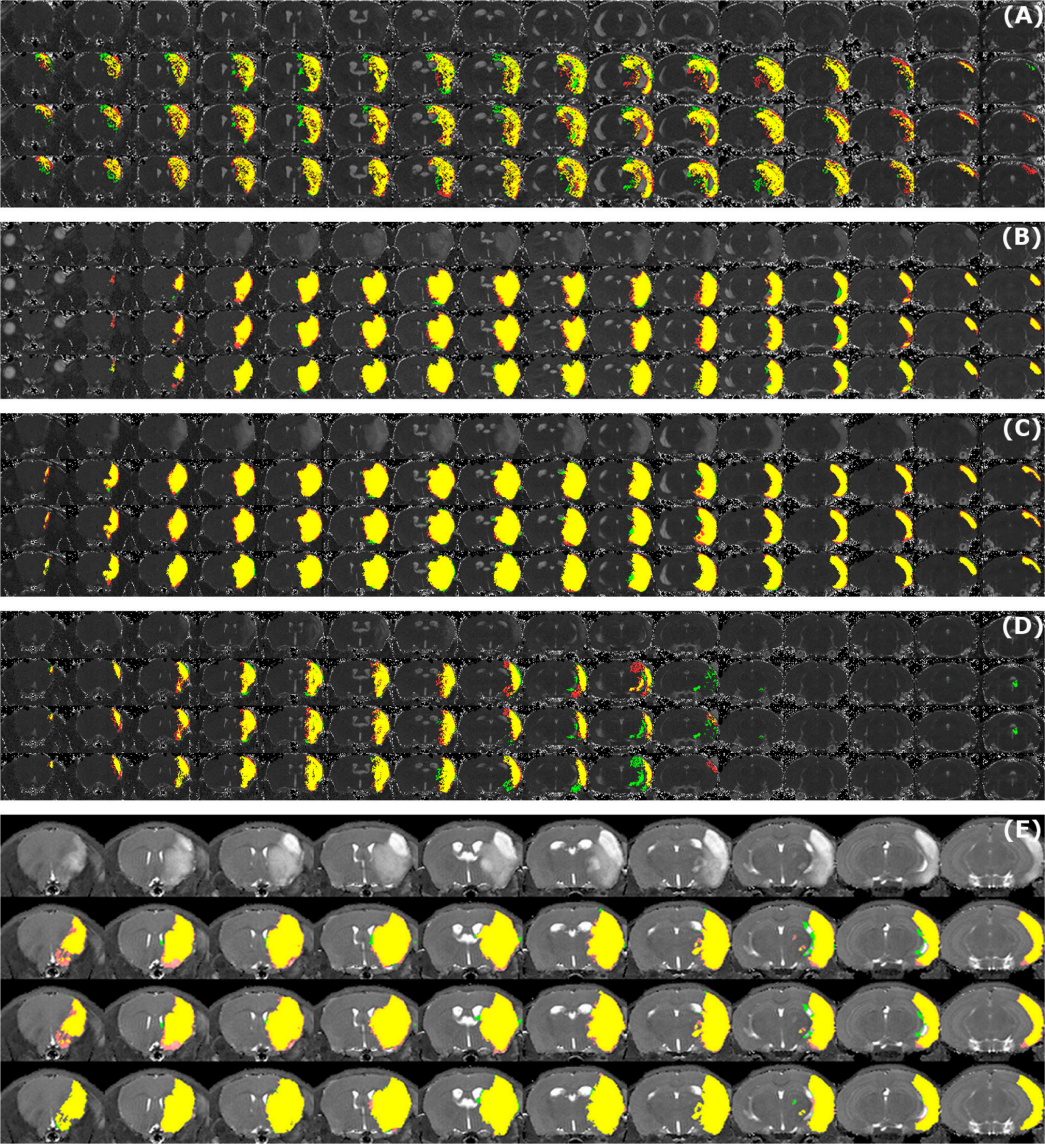
**Examples of segmentation results**. Data sets of different origin [*Leiden-Set* **(A–D)**, *Cologne-Set* **(E)**] were acquired at different time points after infarction induction [4 h **(A)**, 24 h **(B)**, 48 h **(C,E)** and 8 d **(D)**]. For each of the image panels, the first row illustrates reformatted raw image stack and the rest of the rows provide overlaid segmentation results by: automated (green) *vs*. Observer 1 (red), automated (green) *vs*. Observer 2 (red), Observer 1 (green) *vs*. Observer 2 (red), respectively. The regions where two segmentations overlap are colored in yellow. Image intensity was enhanced for visualization purposes.

**Figure 3:**
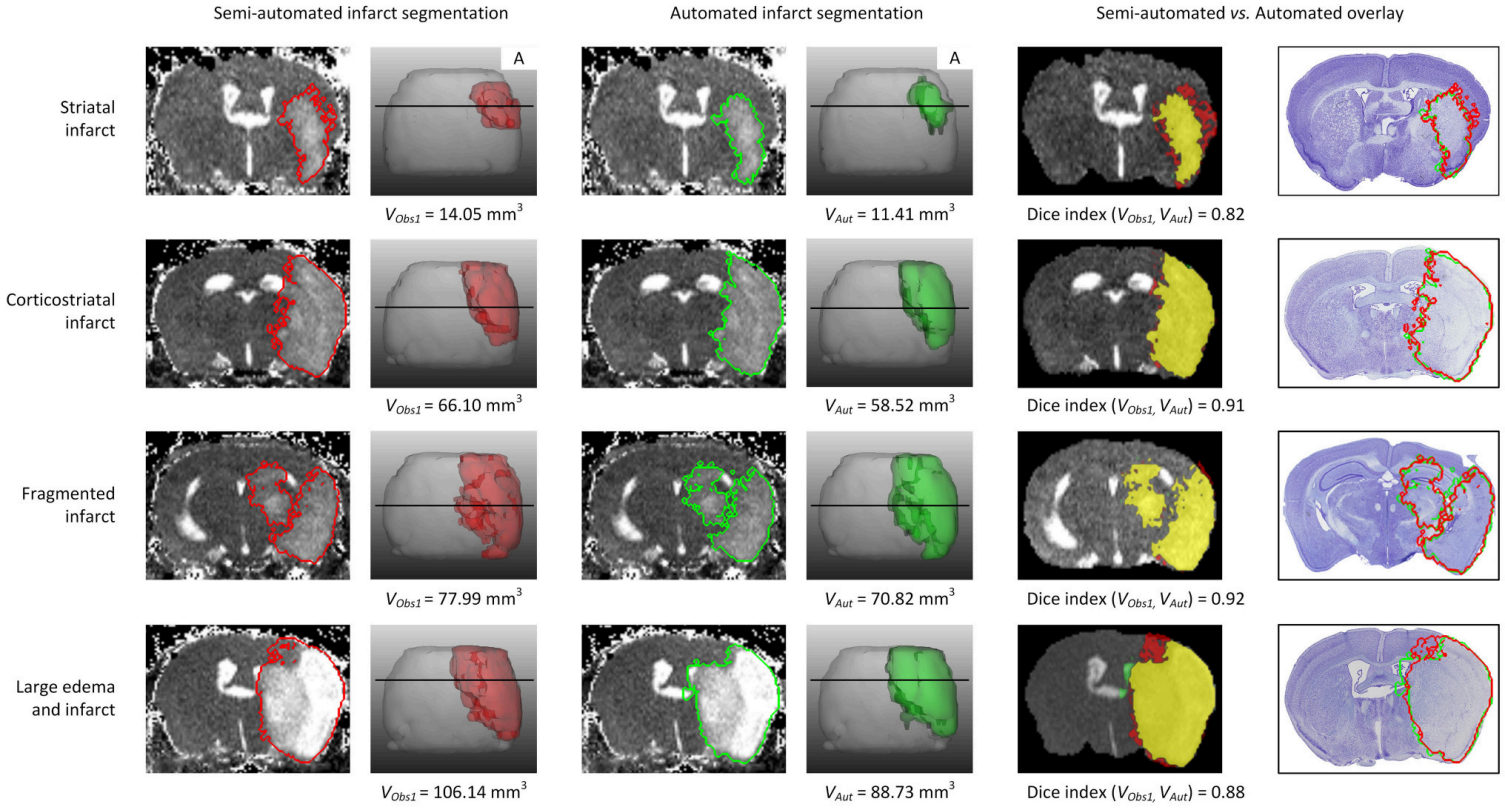
**Example traces of different infarct anatomies in a single slice and complete 3D brain**. Lesions (striatal, corticostriatal, fragmented and with large edema compressing the ventricle) were segmented using the semi-automated (red delineated area) and automated (green delineated area) approaches. The fifth column shows the overlap between both methods, in yellow. The last column shows the overlay of both approaches on the corresponding Nissl stained histological section. “A” stands for anterior. *V*_*Obs*1_ corresponds to the volume obtained by performing semi-automated stroke segmentation by Observer 1 (IM). *V*_*Aut*_ corresponds to the volume obtained by the automated method. The Dice index between Observer 1 and Observer 2 for the same data sets was: 0.82 for striatal infarct, 0.91 for corticostriatal infarct, 0.92 for fragmented infarct and 0.88 for the large edema case.

Sample segmentation results on the data sets of different origin and acquired at different time points after infarction induction are shown in Figure 2. Supplementary Material 1 provides corresponding segmentation results on all 121 data sets. Figure 3 illustrates segmented striatal (Str) and corticostriatal (Ctx+Str) lesions, as well as those with fragmented infarct areas and with large edema and morphed ventricles. The results show good agreement between both segmentation methods as well as with the accompanying histological sections.

**Figure 4:**
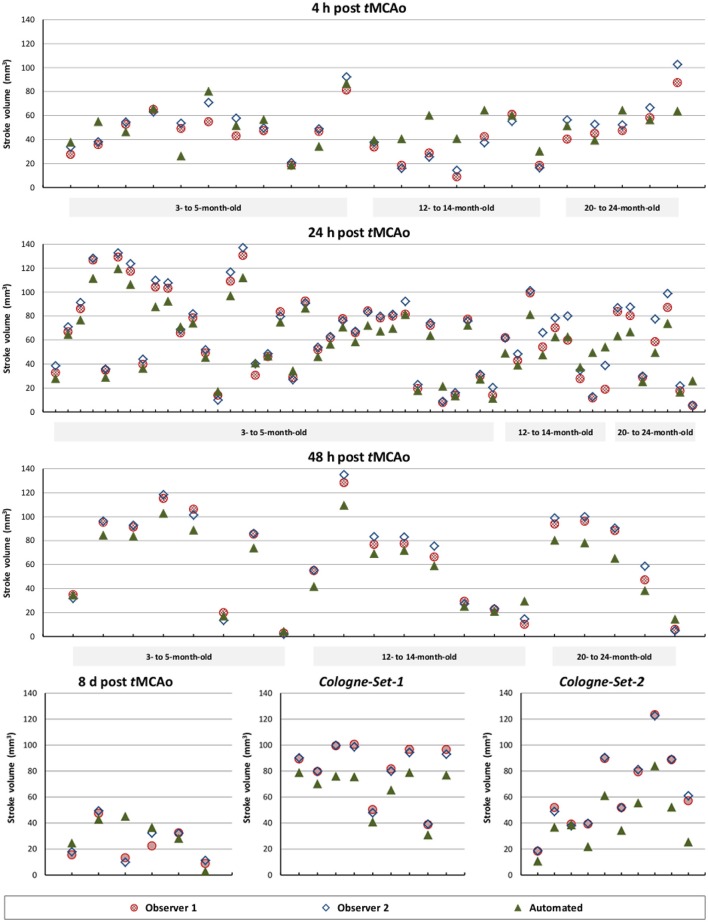
**Comparison of total stroke volume between our automated method and human observers, for all test data sets, different times after stoke induction and different mouse ages**.

The results of experiments for all age groups at the various time points after stroke induction from *Leiden-Set* and *Cologne-Sets* in terms of the absolute volume difference, ICC and Dice index are reported in Table 3. The mean per-group absolute volume difference in the *Leiden-Set* ranged from 5.9 to 18.4 mm^3^, for the *Cologne-Set-1* it was 15.3 mm^3^ (for 18 h) and 14.2 mm^3^ (for 4 d) and for the *Cologne-Set-2* it was 22.0 mm^3^. Infarct volumes of all groups, from both observers and by the automated method are presented in Figure 4.

**Table 3:** **Comparison of performance for infarct lesions for different groups of interest in terms of absolute volume difference, ICC and Dice index**.

**Age (months)**	**Infarct induction**	**Absolute volume difference (mm^3^)**	**ICC**	**Dice index**		
				**Automated *vs*. Observer 1**	**Automated *vs*. Observer 2**	**Observer 1 *vs*. Observer 2**
3–5	4 h	9.2 ± 8.0	0.806	0.78 ± 0.08	0.78 ± 0.07	0.87 ± 0.04
	24 h	8.4 ± 4.9	0.957	0.87 ± 0.08	0.87 ± 0.08	0.92 ± 0.05
	24 h (Str)	5.9 ± 3.7	0.966	0.80 ± 0.12	0.79 ± 0.12	0.88 ± 0.06
	24 h (Ctx+Str)	8.4 ± 4.9	0.944	0.88 ± 0.06	0.88 ± 0.06	0.93 ± 0.04
	48 h	8.0 ± 5.6	0.971	0.84 ± 0.13	0.84 ± 0.13	0.91 ± 0.07
	8 d	11.3 ± 10.1	0.491	0.65 ± 0.17	0.63 ± 0.21	0.79 ± 0.13
10–12	4 h	18.4 ± 11.3	0.384	0.63 ± 0.14	0.63 ± 0.13	0.79 ± 0.08
	24 h	15.4 ± 9.7	0.622	0.78 ± 0.18	0.78 ± 0.15	0.87 ± 0.09
	48 h	11.1 ± 6.3	0.934	0.81 ± 0.13	0.81 ± 0.11	0.90 ± 0.08
20–24	4 h	12.9 ± 10.0	0.445	0.80 ± 0.03	0.77 ± 0.04	0.84 ± 0.04
	24 h	15.0 ± 7.3	0.852	0.77 ± 0.19	0.72 ± 0.17	0.88 ± 0.05
	48 h	16.8 ± 5.1	0.878	0.73 ± 0.26	0.68 ± 0.29	0.84 ± 0.16
3	18 h	15.3 ± 6.4	0.793	0.88 ± 0.03	0.89 ± 0.03	0.98 ± 0.01
	4 d	14.2 ± 4.4	0.964	0.85 ± 0.01	0.87 ± 0.01	0.97 ± 0.00
2, 12	48 h	22.0 ± 12.2	0.552	0.86 ± 0.07	0.85 ± 0.07	0.97 ± 0.02

*Different row colors correspond to different data sets: Leiden-Set (white), Cologne-Set-1 (beige) and Cologne-Set-2 (blue), respectively*.

The mean per-group Dice index for Observer 1 *vs*. Observer 2 was between 0.79 and 0.98, whereas for automated *vs*. observers it was ranging from 0.63 to 0.88 (Observer 1) and from 0.63 to 0.89 (Observer 2).

**Figure 5:**
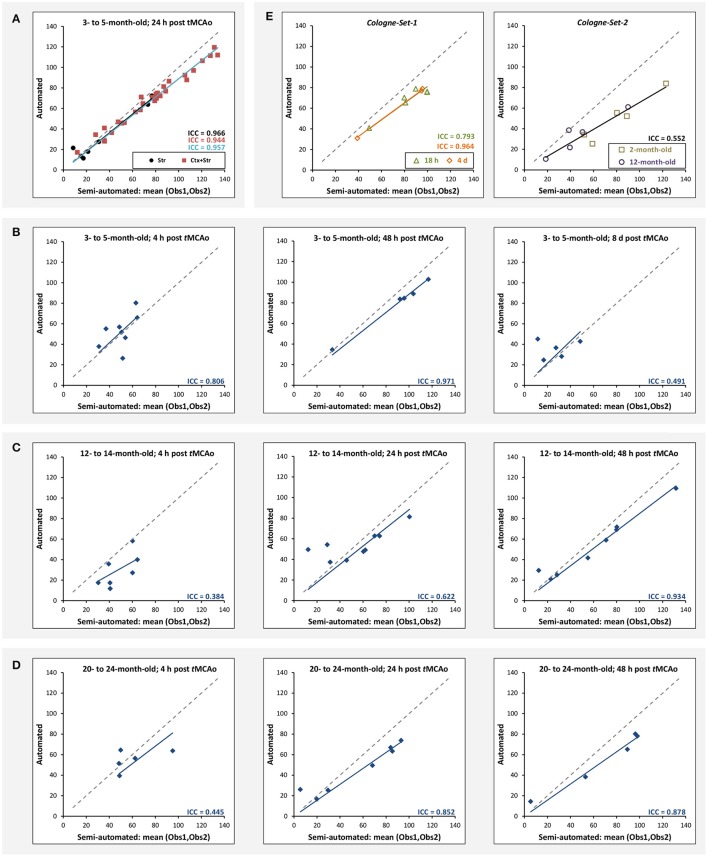
**Correlation of lesion volumes calculated in a semi-automated fashion by observers ***vs***. the automated method. (A)** 3- to 5-month-old mice, at 24 h after *t*MCAo for striatal (Str) and corticostriatal (Ctx+Str) lesions. **(B)** 3- to 5-month-old mice, MRI at 4 h, 48 h and 8 d after *t*MCAo. **(C)** 12- to 14-month-old mice, MRI at 4 h, 24 h and 48 h after *t*MCAo. **(D)** 20- to 24-month-old mice, MRI at 4 h, 24 h and 48 h after *t*MCAo. **(E)** MRI using different hardware and software at 18 h and 4 d (*Cologne-Set-1*) and 48 h (*Cologne-Set-2*) after *t*MCAo. The solid lines provide the trend of the corresponding data group and the dashed gray line corresponds to equal values on both axes and is shown as a reference.

**Figure 6:**
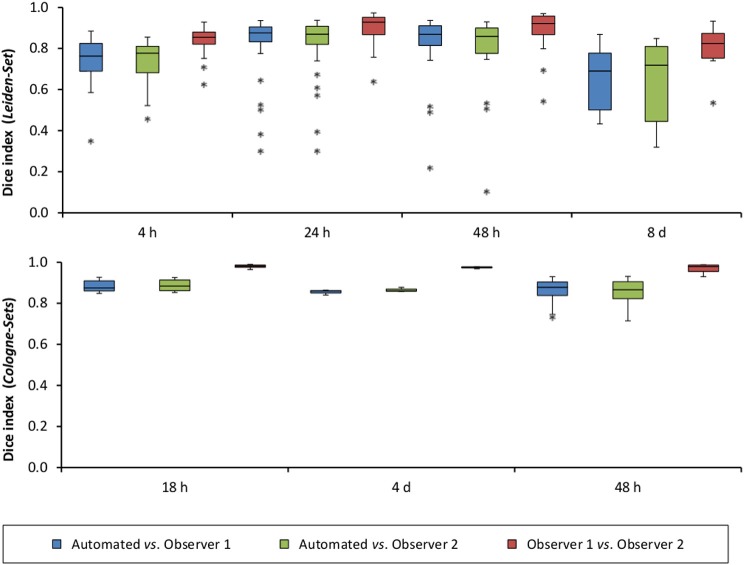
**Distribution of Dice index for each of the analyzed time points after stroke induction and for all three data sets: ***Leiden-Set***, ***Cologne-Set-1*** (18 h, 4 d) and ***Cologne-Set-2*** (48 h)**.

**Figure 7:**
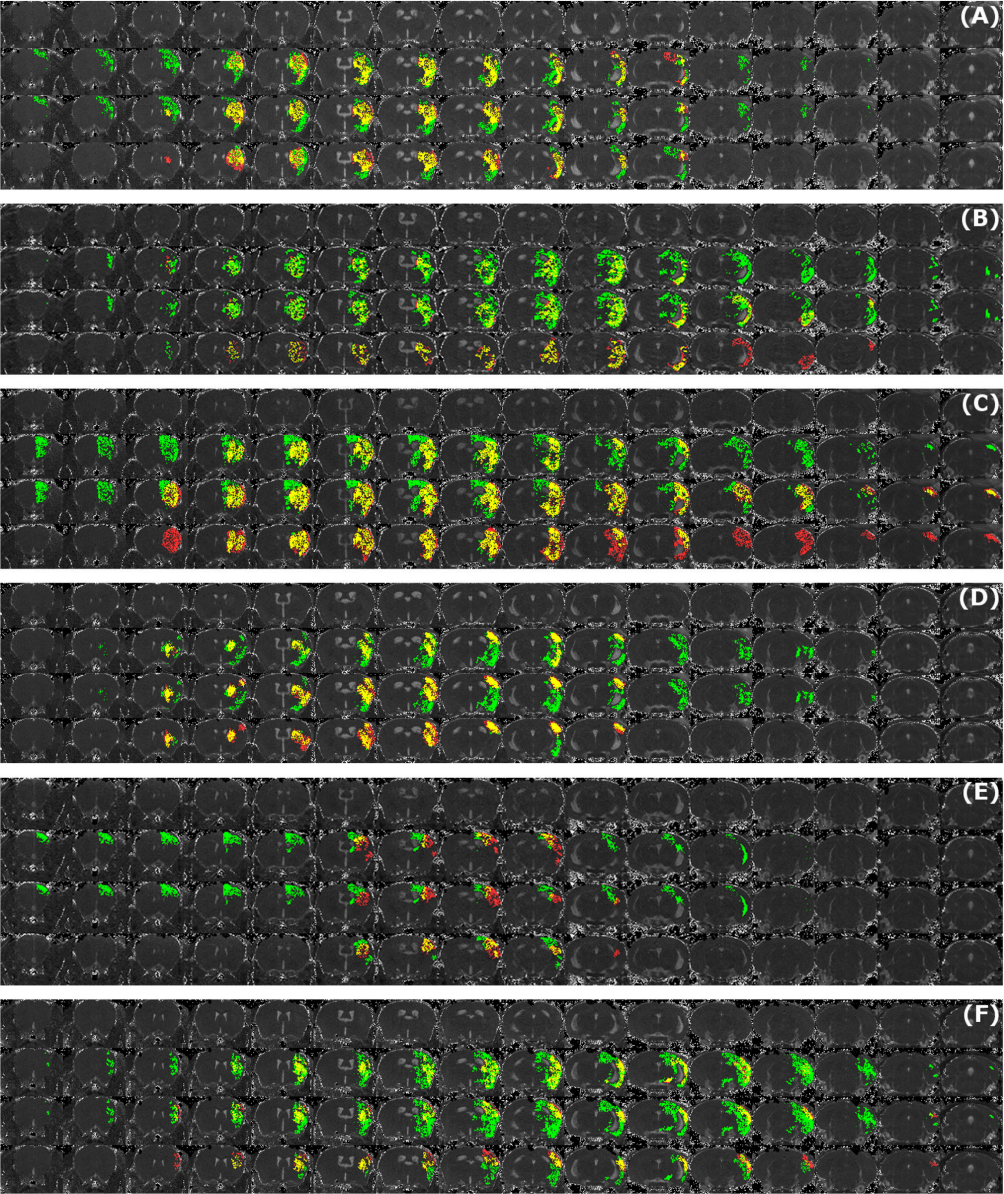
**Examples of segmentation results that exhibit large disagreement between two observers**. Data sets were acquired at different time points after infarct induction [4 h **(A,B)**, 24 h **(C)**, 48 h **(D,E)** and 8 d **(F)**]. For each of the image panels, the first row illustrates reformatted raw image stack and the rest of the rows provide overlaid segmentation results by: automated (green) *vs*. Observer 1 (red), automated (green) *vs*. Observer 2 (red), Observer 1 (green) *vs*. Observer 2 (red), respectively. The regions where two segmentations overlap are colored in yellow. Image intensity was enhanced for visualization purposes.

Figure 5A illustrates the mean infarct volume obtained by the two observers *vs*. the corresponding automated volume for our primary group, for both Str and Ctx+Str lesions. This plot shows very high degree of correlation with an ICC of 0.957 (ICC = 0.966 for Str lesions and ICC = 0.944 for Ctx+Str lesions). Figures 5B–E show the corresponding information for all the other groups. At 24 h and 48 h, the data match the primary validation group. However, at 4 h and 8 d after stroke induction the regression line is less perfect: ICC = 0.806 at 4 h and ICC = 0.491 at 8 d for 3- to 5-month-old mice, ICC = 0.384 at 4 h for 12- to 14-month-old mice and ICC = 0.445 at 4 h for 20- to 24-month-old mice. Figure 5E shows that our method is also robust with respect to data obtained from different imaging hardware using different acquisition parameters, time after stroke induction and age: ICC = 0.793 at 18 h, ICC = 0.964 at 4 days and ICC = 0.552 for 1- to 2-year-old mice scanned at 48 h after stroke induction. Figure 6 shows distribution of Dice index for different time points. Here, again, the results of the 24 h and 48 h groups are superior to those of the 4 h and 8 d groups: mean Dice index of 0.68 ÷ 0.87 at 24 h and 48 h and of 0.63 ÷ 0.80 for 4 h and 8 d for all groups, respectively. Finally, Figure 7 provides segmentation results on the data sets that exhibit large disagreement between two observers. These data sets are outliers of the corresponding box-whisker plot on Figure 6.

We have also performed a parameter sensitivity study to assess stability of our algorithm with respect to the parameters. For this, we changed, in turn, the value of each of the parameters listed in Table 2 by ± 50% and recalculated the results. Our approach turned out to be highly robust with respect to both validation metrics: maximal change for the mean absolute volume difference was less than 6%, and for the mean Dice index it was less than 1.2%.

## Discussion

The major challenges in stroke segmentation in pre-clinical MRI are (i) irregular lesion volume and shape, (ii) low resolution compared to clinical imaging data, (iii) fluctuating contrast and (iv) considerable brain deformation and asymmetry because of the induced stroke. The presented approach addresses these challenges by estimating intensity distributions per scan from the contralateral, unaffected, hemisphere and comparing these with the affected hemisphere. With our approach, an ischemic lesion in a mouse brain can be successfully segmented from T2 MR images (Milidonis et al., 2015) in a fully automated manner.

Our approach has a number of advantages over existing methods for stroke segmentation in small animal data:

1. A main asset of our automated analysis approach is that it takes only few minutes to analyze an MRI volume (containing 16 slices) and is therefore at least an order of magnitude faster than the semi-automated method (~ 60 min). It is also more robust because of its objective character excluding potential observer bias. Thus, although histology remains the gold standard for assessment of lesion formation in pre-clinical stroke research, its downsides (that one needs to sacrifice the animal for a given time point and the fact that results are confounded by morphological changes due to the processing of fragile tissue) make MRI more desirable when one investigates the development of lesions over time. It is not unexpected that MRI is establishing itself as the main diagnostic modality for such studies, as it allows automated data processing and was shown to correspond well with histology (Milidonis et al., 2015).
2. Our method is robust with respect to different infarct shapes and volumes, even for small fragmented lesions or extremely large lesions where ventricles become deformed due to edema formation in and around the lesion. It performed well for a heterogeneous group of 121 brain scans, including different MRI scanners (noise levels, resolution), different lesion anatomies (cortical, corticostriatal, with large edema, fragmented), over different time points after infarct induction (from 4 h up to 8 d). In particular, it exhibited robust performance on data acquired from different MRI hardware—with differences in slice number and thickness—without the need for modification of the model or registration/segmentation parameter settings. We expect that our method is also suitable for other MRI lesion detection sequences, such as DWI, and perhaps even for different rodent species, with only limited model and parameter adjustments required. It is important to point out that, as we have mentioned in Section “Parameter Selection,” all parameters of our algorithm were optimized with respect to the entire *Leiden-Set*, which means that our method was completely blinded to the properties of each particular data set. However, in our analysis we subdivided the *Leiden-Set* into smaller groups, based on age and time after infarct induction, and also report results on two unseen sets of images (*Cologne-Sets*). This naturally results in suboptimal performance on each particular group, confirmed, e.g., by lower ICCs reported in Figure 5 and Table 3. This effect is especially pronounced in relatively small groups as these were underrepresented at the parameter tuning stage. Optimizing the parameters for each particular group will help improving the performance, which, however, will inevitably be limited by complexity of the data.
3. Unlike numerous published methods in the field (Jacobs et al., 1999a,b, 2000, 2001a,b; Bernarding et al., 2000; Li et al., 2002; Sotak, 2002; van Dorsten et al., 2002; Soltanian-Zadeh et al., 2003; Wang et al., 2007), our algorithm operates on a single contrast (T2) and does not require a pre-scan.
4. Unlike other methods developed for small animal data (HRS by Ghosh et al., 2011, 2012, 2014 and ISODATA by Jacobs et al., 1999a,b, 2000, 2001a,b), the ischemic lesion segmentation approach presented here only makes use of the quantitative T2 maps. Contrary to T2-weighted MRI data, true quantitative MRI maps (T2 or others) are comparable across research centers, whereas parameter-weighted images (T2-weighted or others) are subjective estimates derived for better discrimination of the object of interest from the background and are not comparable across research centers due to arbitrary operator choice of TE and TR values. A combination of complementary quantitative parameters, for instance, T2 and ADC values, on the other hand, can in some cases improve discrimination not only between the different objects present in an image, but also between the different subcategories of the object (Hoehn-Berlage et al., 1995a, 1997). This improvement, however, comes at the expense of slightly increased scan times and additional data analysis.
5. Our approach is generic as we do not make any specific assumptions or create a general model that would describe all of our data. Instead, the stroke area is segmented on each mouse brain MR volume by using the intensity distributions of the background and the ventricles calculated from the very same image. In all validation experiments, our method was completely blinded to the properties (origin, age, time point) of each particular brain volume, meaning that the same parameter settings were used for all data sets (including those acquired with different hardware and software compared to the training set).

The main pitfall of our approach is its multi-step nature. This type of complex algorithm, and our method in particular, is sensitive to propagation of errors made at earlier stages. Our experiments show that success or failure of the registration with label propagation, the first step in our algorithm, has significant impact on subsequent segmentation steps. More precisely, the largest segmentation errors were caused by inability to accurately segment the ventricles, e.g., when they were virtually invisible due to a large stroke area. In particular, inability to achieve high-quality registration due to large difference between the atlas scan and the rest of the data, also explains suboptimal performance of our method on *Cologne-Sets*.

In the absence of histology, semi-automated segmentation by experts has always been used as the reference standard in stroke quantification, which was also the case for this work. Thus, during development of any automated stroke segmentation method the main goal is to achieve performance comparable to that by experts. This would allow bypassing the manual observer bias and, hence, lead to more objective quantification of the stroke region. In this work, we evaluated the performance of our method by comparing it to two observers (separately and combined) and by analyzing the inter-observer variability.

Our results indicate that quantification of the infarct in its acute phase (4 h after induction) remains challenging, both for our automated approach and for the human observers. During this early phase of lesion development, T2 enhancement is still very weak as edema is only slowly evolving, so that in some cases it may barely reach above the normal contralateral value. It should be stated that also histological lesion demarcation at this very early time point is unreliable, which admittedly complicates infarct detection during the first hours after stroke induction in experimental animal models.

## Author contributions

Conceived and designed the study: IM, AK, OD, MH, BL, AvdM. Performed animal experiments, *t*MCAo surgery and acquired MRI data: IM. Performed histopathology: IM, NR. Performed semi-automated ischemic lesion segmentation: IM, SdJ. Developed the *automated ischemic lesion segmentation in MRI mouse brain data after tMCAo occlusion* algorithm: AK, OD. Analyzed the data: IM, AK, OD, MH, BL, AvdM. Wrote the manuscript: IM, AK, OD. Discussed the results and commented on the manuscript: IM, AK, OD, MW, MH, BL, AvdM.

## Funding

The authors acknowledge funding from the: Dutch Heart Foundation (2011T055; MW), ZonMW Veni grant (MW), Dutch Brain Foundation (F2014(1)-22; MW), Centre for Medical Systems Biology (CMSB) in the framework of the Netherlands Genomics Initiative (NGI) (AvdM), FP7 EUROHEADPAIN (no. 602633; AvdM), Marie Curie IAPP Program BRAINPATH (no. 612360; AK, AvdM, MH), FP7/2007-2013 under grant agreement no. 604102—Human Brain Project (AK, AvdM, BL), H2020-Marie Skłodowska-Curie Action Research and Innovation Staff Exchange (RISE) Grant 644373-PRISAR (BL), and Dutch Technology Foundation STW (as part of the STW project 12721: “Genes in Space” under the IMAGENE perspective program; OD).

### Conflict of interest statement

The authors declare that the research was conducted in the absence of any commercial or financial relationships that could be construed as a potential conflict of interest.

## Supplementary material

Supplementary Material 1 **Segmentation results on the entire validation set**.

## References

[B1] Bernarding J. Braun J. Hohmann J. Mansmann U. Hoehn-Berlage M. Stapf C.. (2000). Histogram-based characterization of healthy and ischemic brain tissues using multiparametric MR imaging including apparent diffusion coefficient maps and relaxometry. Magn. Reson. Med. 43, 52–61. 10.1002/(SICI)1522-2594(200001)43:1<52::AID-MRM7>3.0.CO;2-5 10642731

[B2] Chan T. F. Vese L. A. (2001). Active contours without edges. IEEE Trans. Image Process. 10, 266–277. 10.1109/83.902291 18249617

[B3] Couillard-Despres S. Finkl R. Winner B. Plotz S. Wiedermann D. Aigner R.. (2008). *In vivo* optical imaging of neurogenesis: watching new neurons in the intact brain. Mol. Imaging 7, 28–34. 10.2310/7290.2008.0004 18384721

[B4] Dice L. R. (1945). Measures of the amount of ecologic association between species. Ecology 26, 297–302. 10.2307/1932409

[B5] Dijkhuizen R. M. Nicolay K. (2003). Magnetic resonance imaging in experimental models of brain disorders. J. Cereb. Blood Flow Metab. 23, 1383–1402. 10.1097/01.WCB.0000100341.78607.EB 14663334

[B6] Ding G. Jiang Q. Li L. Zhang L. Zhang Z. G. Soltanian-Zadeh H.. (2006). Characterization of cerebral tissue by MRI map ISODATA in embolic stroke in rat. Brain Res. 1084, 202–209. 10.1016/j.brainres.2006.02.054 16566903

[B7] Ding G. Jiang Q. Zhang L. Zhang Z. Knight R. A. Soltanian-Zadeh H.. (2004). Multiparametric ISODATA analysis of embolic stroke and rt-PA intervention in rat. J. Neurol. Sci. 223, 135–143. 10.1016/j.jns.2004.05.017 15337614

[B8] Dirnagl U. (2016). Thomas Willis lecture: is translational stroke research broken, and if so, how can we fix it? Stroke 47, 2148–2153. 10.1161/strokeaha.116.013244 27354221

[B9] Donath S. An J. Lee S. L. Gertz K. Datwyler A. L. Harms U.. (2016). Interaction of ARC and Daxx: a novel endogenous target to preserve motor function and cell loss after focal brain ischemia in mice. J. Neurosci. 36, 8132–8148. 10.1523/jneurosci.4428-15.2016 27488634 PMC4971361

[B10] Dufour A. Shinin V. Tajbakhsh S. Guillén-Aghion N. Olivo-Marin J. C. Zimmer C. (2005). Segmenting and tracking fluorescent cells in dynamic 3-D microscopy with coupled active surfaces. IEEE Trans. Image Process. 14, 1396–1410. 10.1109/tip.2005.852790 16190474

[B11] Ghosh N. Recker R. Shah A. Bhanu B. Ashwal S. Obenaus A. (2011). Automated ischemic lesion detection in a neonatal model of hypoxic ischemic injury. J. Magn. Reson. Imaging 33, 772–781. 10.1002/jmri.22488 21448940

[B12] Ghosh N. Sun Y. Bhanu B. Ashwal S. Obenaus A. (2014). Automated detection of brain abnormalities in neonatal hypoxia ischemic injury from MR images. Med. Image Anal. 18, 1059–1069. 10.1016/j.media.2014.05.002 25000294 PMC4145020

[B13] Ghosh N. Yuan X. Turenius C. I. Tone B. Ambadipudi K. Snyder E. Y.. (2012). Automated core–penumbra quantification in neonatal ischemic brain injury. J. Cereb. Blood Flow Metab. 32, 2161–2170. 10.1038/jcbfm.2012.121 22929436 PMC3520032

[B14] Hoehn-Berlage M. Eis M. Back T. Kohno K. Yamashita K. (1995a). Changes of relaxation times (T1, T2) and apparent diffusion coefficient after permanent middle cerebral artery occlusion in the rat: temporal evolution, regional extent, and comparison with histology. Magn. Reson. Med. 34, 824–834. 10.1002/mrm.1910340607 8598809

[B15] Hoehn-Berlage M. Hossmann K. A. Busch E. Eis M. Schmitz B. Gyngell M. L. (1997). Inhibition of nonselective cation channels reduces focal ischemic injury of rat brain. J. Cereb. Blood Flow Metab. 17, 534–542. 10.1097/00004647-199705000-00007 9183291

[B16] Hoehn-Berlage M. Norris D. G. Kohno K. Mies G. Leibfritz D. Hossmann K. A. (1995b). Evolution of regional changes in apparent diffusion coefficient during focal ischemia of rat brain: the relationship of quantitative diffusion NMR imaging to reduction in cerebral blood flow and metabolic disturbances. J. Cereb. Blood Flow Metab. 15, 1002–1011. 10.1038/jcbfm.1995.126 7593332

[B17] Jacobs M. A. Knight R. A. Soltanian-Zadeh H. Zheng Z. G. Goussev A. V. Peck D. J.. (2000). Unsupervised segmentation of multiparameter MRI in experimental cerebral ischemia with comparison to T2, diffusion, and ADC MRI parameters and histopathological validation. J. Magn. Reson. Imaging 11, 425–437. 10.1002/(SICI)1522-2586(200004)11:4<425::AID-JMRI11>3.0.CO;2-0 10767072

[B18] Jacobs M. A. Knight R. A. Windham J. P. Zhang Z. G. Soltanian-Zadeh H. Goussev A. V.. (1999b). Identification of cerebral ischemic lesions in rat using Eigenimage filtered magnetic resonance imaging. Brain Res. 837, 83–94. 10.1016/S0006-8993(99)01582-6 10433991

[B19] Jacobs M. A. Mitsias P. Soltanian-Zadeh H. Santhakumar S. Ghanei A. Hammond R.. (2001b). Multiparametric MRI tissue characterization in clinical stroke with correlation to clinical outcome: part 2. Stroke 32, 950–957. 10.1161/01.STR.32.4.950 11283396

[B20] Jacobs M. A. Windham J. P. Soltanian-Zadeh H. Peck D. J. Knight R. A. (1999a). Registration and warping of magnetic resonance images to histological sections. Med. Phys. 26, 1568–1578. 10.1118/1.598671 10501057

[B21] Jacobs M. A. Zhang Z. G. Knight R. A. Soltanian-Zadeh H. Goussev A. V. Peck D. J.. (2001a). A model for multiparametric MRI tissue characterization in experimental cerebral ischemia with histological validation in rat: part 1. Stroke 32, 943–949. 10.1161/01.STR.32.4.943 11283395

[B22] Kilkenny C. Browne W. J. Cuthill I. C. Emerson M. Altman D. G. (2010). Improving bioscience research reporting: the ARRIVE guidelines for reporting animal research. PLoS Biol. 8:e1000412. 10.1371/journal.pbio.1000412 20613859 PMC2893951

[B23] Klein S. Staring M. Murphy K. Viergever M. A. Pluim J. (2010). elastix: a toolbox for intensity-based medical image registration. IEEE Trans. Med. Imaging 29, 196–205. 10.1109/TMI.2009.2035616 19923044

[B24] Leithner C. Füchtemeier M. Jorks D. Mueller S. Dirnagl U. Royl G. (2015). Infarct volume prediction by early magnetic resonance imaging in a murine stroke model depends on ischemia duration and time of imaging. Stroke 46, 3249–3259. 10.1161/strokeaha.114.007832 26451016

[B25] Li F. Liu K. F. Silva M. D. Meng X. Gerriets T. Helmer K. G.. (2002). Acute postischemic renormalization of the apparent diffusion coefficient of water is not associated with reversal of astrocytic swelling and neuronal shrinkage in rats. ANJR Am. J. Neuroradiol. 23, 180–188. 11847039 PMC7975252

[B26] Llovera G. Liesz A. (2016). The next step in translational research: lessons learned from the first preclinical randomized controlled trial. J. Neurochem. 10.1111/jnc.13516 26968835

[B27] Longa E. Z. Weinstein P. R. Carlson S. Cummins R. (1989). Reversible middle cerebral artery occlusion without craniectomy in rats. Stroke 20, 84–91. 10.1161/01.STR.20.1.84 2643202

[B28] Milidonis X. Marshall I. Macleod M. R. Sena E. S. (2015). Magnetic resonance imaging in experimental stroke and comparison with histology: systematic review and meta-analysis. Stroke 46, 843–851. 10.1161/strokeaha.114.007560 25657177

[B29] Moraga A. Gómez-Vallejo V. Cuartero M. I. Szczupak B. San Sebastián E. Markuerkiaga I.. (2016). Imaging the role of toll-like receptor 4 on cell proliferation and inflammation after cerebral ischemia by positron emission tomography. J. Cereb. Blood Flow Metab. 36, 702–708. 10.1177/0271678X15627657 26787106 PMC4821030

[B30] Niimi T. Imai K. Maeda H. Ikeda M. (2007). Information loss in visual assessments of medical images. Eur. J. Radiol. 61, 362–366. 10.1016/j.ejrad.2006.09.009 17067772

[B31] Rekik I. Allassonnière S. Carpenter T. K. Wardlaw J. M. (2012). Medical image analysis methods in MR/CT-imaged acute-subacute ischemic stroke lesion: Segmentation, prediction and insights into dynamic evolution simulation models. A critical appraisal. NeuroImage Clin. 1, 164–178. 10.1016/j.nicl.2012.10.003 24179749 PMC3757728

[B32] Rousson M. Deriche R. (2002). A variational framework for active and adaptative segmentation of vector valued images, in MOTION'02: Proceedings Workshop on Motion and Video Computing (Washington, DC: IEEE Computer Society), 56.

[B33] Soltanian-Zadeh H. Pasnoor M. Hammoud R. Jacobs M. A. Patel S. C. Mitsias P. D.. (2003). MRI tissue characterization of experimental cerebral ischemia in rat. J. Magn. Reson. Imaging 17, 398–409. 10.1002/jmri.10256 12655578

[B34] Sotak C. H. (2002). The role of diffusion tensor imaging in the evaluation of ischemic brain injury – a review. NMR Biomed. 15, 561–569. 10.1002/nbm.786 12489102

[B35] Sunkin S. M. Ng L. Lau C. Dolbeare T. Gilbert T. L. Thompson C. L.. (2013). Allen brain atlas: an integrated spatio-temporal portal for exploring the central nervous system. Nucl. Acids Res. 41, D996–D1008. 10.1093/nar/gks1042 23193282 PMC3531093

[B36] van Dorsten F. A. Olàh L. Schwindt W. Grüne M. Uhlenküken U. Pillekamp F.. (2002). Dynamic changes of ADC, perfusion, and NMR relaxation parameters in transient focal ischemia of rat brain. Magn Reson. Med. 47, 97–104. 10.1002/mrm.10021 11754448

[B37] Wang Y. Cheung P. T. Shen G. X. Bhatia I. Wu E. X. Qiu D.. (2007). Comparing diffusion-weighted and T2-weighted MR imaging for the quantification of infarct size in a neonatal rat hypoxic-ischemic model at 24 h post-injury. Int. J. Dev. Neurosci. 25, 1–5. 10.1016/j.ijdevneu.2006.12.003 17229540

[B38] Weber R. Ramos-Cabrer P. Hoehn M. (2006). Present status of magnetic resonance imaging and spectroscopy in animal stroke models. J. Cereb. Blood Flow Metab. 26, 591–604. 10.1038/sj.jcbfm.9600241 16292254

[B39] Yao X. Derugin N. Manley G. T. Verkman A. S. (2015). Reduced brain edema and infarct volume in aquaporin-4 deficient mice after transient focal cerebral ischemia. Neurosci. Let. 584, 368–372. 10.1016/j.neulet.2014.10.040 25449874 PMC4737527

[B40] Yin J. Han P. Tang Z. Liu Q. Shi J. (2015). Sirtuin 3 mediates neuroprotection of ketones against ischemic stroke. J. Cereb. Blood Flow Metab. 35, 1783–1789. 10.1038/jcbfm.2015.123 26058697 PMC4635233

[B41] Yip H. K. Yuen C. M. Chen K. H. Chai H. T. Chung S. Y. Tong M. S.. (2016). Tissue plasminogen activator deficiency preserves neurological function and protects against murine acute ischemic stroke. Int. J. Cardiol. 205, 133–141. 10.1016/j.ijcard.2015.11.168 26736088

[B42] Zheng S. Bai Y. Y. Liu Y. Gao X. Li Y. Changyi Y.. (2015). Salvaging brain ischemia by increasing neuroprotectant uptake via nanoagonist mediated blood brain barrier permeability enhancement. Biomaterials 66, 9–20. 10.1016/j.biomaterials.2015.07.006 26188608

