# Supplementary figures and images for: Automated Ischemic Lesion Segmentation in MRI Mouse Brain Data after Transient Middle Cerebral Artery Occlusion (part 2 of 2)
Source: Front Neuroinform. 2017 Jan 31;11:3. doi: 10.3389/fninf.2017.00003 (PMC5281583; doi:10.3389/fninf.2017.00003)

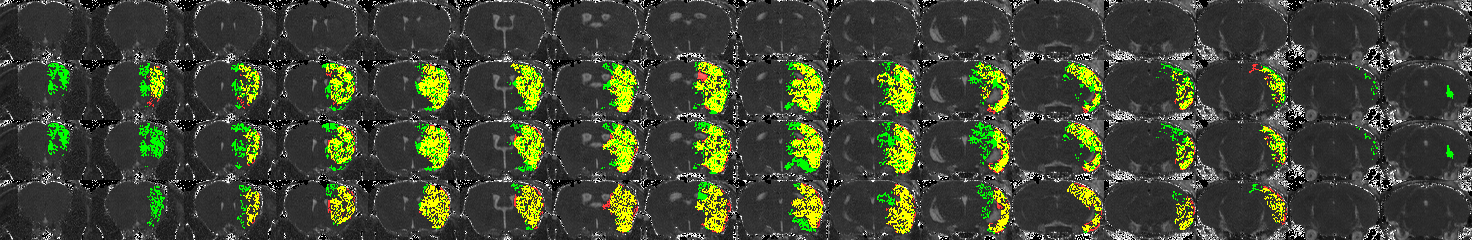

Supplement: Supplementary Material 1 — Segmentation results on the entire validation set. [file DataSheet1.zip › LS_m49_4h_segmentation_results.tif]

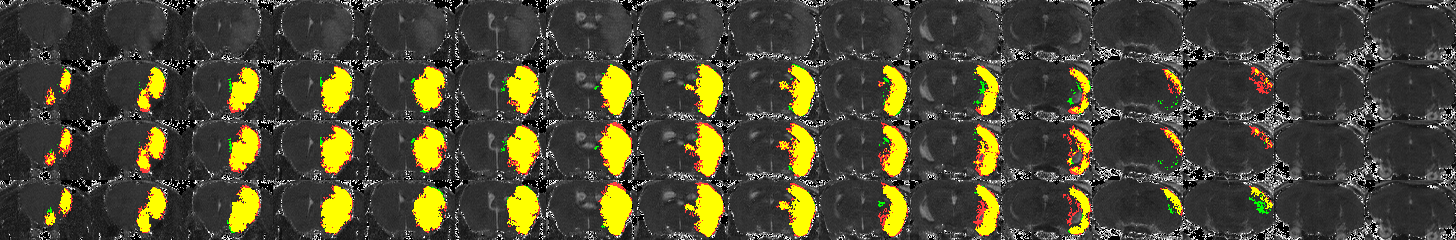

Supplement: Supplementary Material 1 — Segmentation results on the entire validation set. [file DataSheet1.zip › LS_m49_24h_segmentation_results.tif]

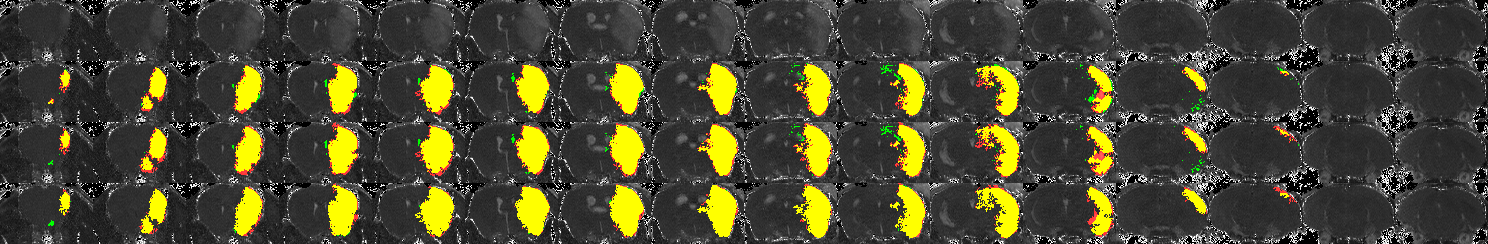

Supplement: Supplementary Material 1 — Segmentation results on the entire validation set. [file DataSheet1.zip › LS_m49_48h_segmentation_results.tif]

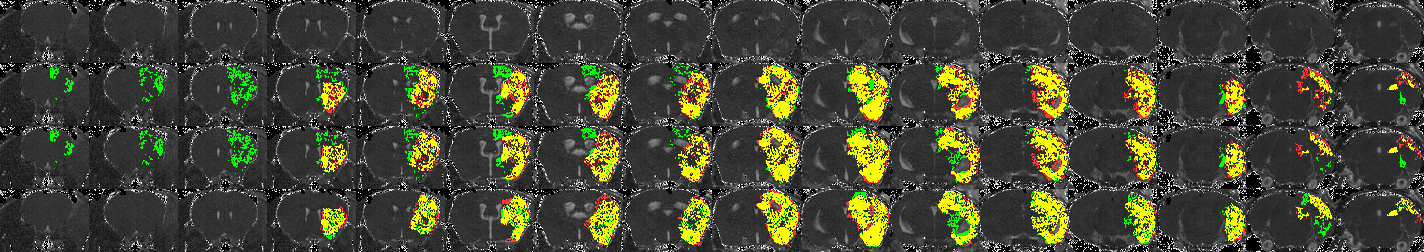

Supplement: Supplementary Material 1 — Segmentation results on the entire validation set. [file DataSheet1.zip › LS_m50_4h_segmentation_results.tif]

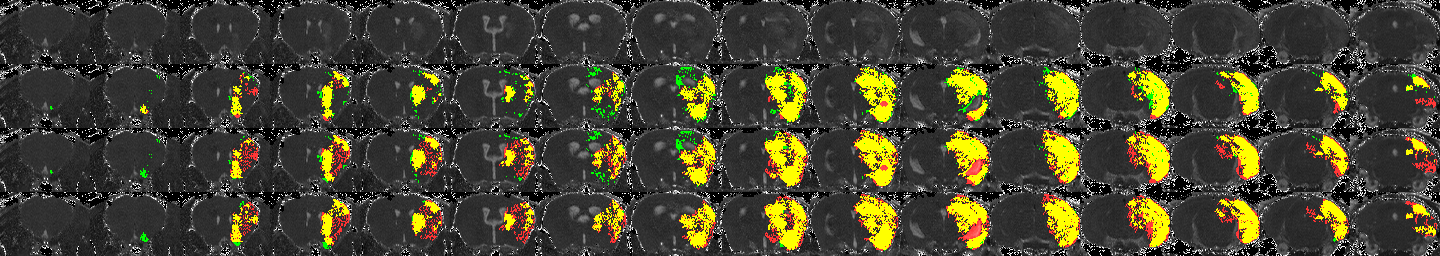

Supplement: Supplementary Material 1 — Segmentation results on the entire validation set. [file DataSheet1.zip › LS_m50_24h_segmentation_results.tif]

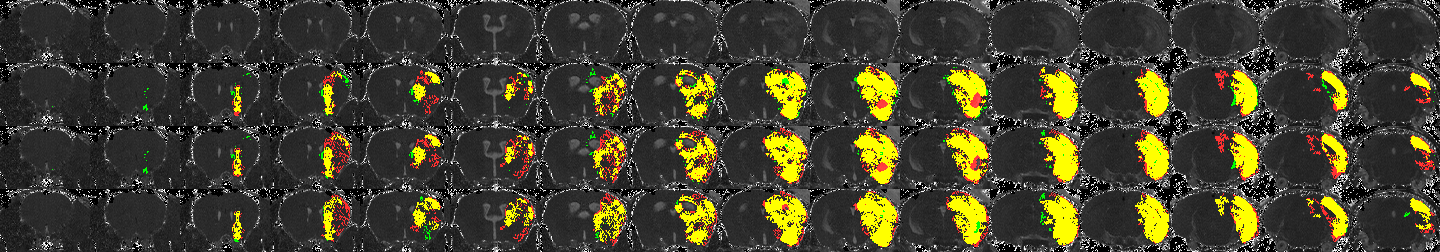

Supplement: Supplementary Material 1 — Segmentation results on the entire validation set. [file DataSheet1.zip › LS_m50_48h_segmentation_results.tif]

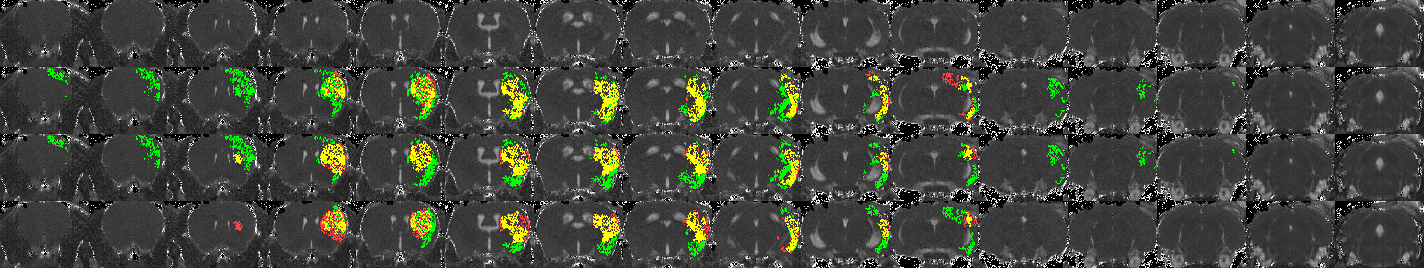

Supplement: Supplementary Material 1 — Segmentation results on the entire validation set. [file DataSheet1.zip › LS_m51_4h_segmentation_results.tif]

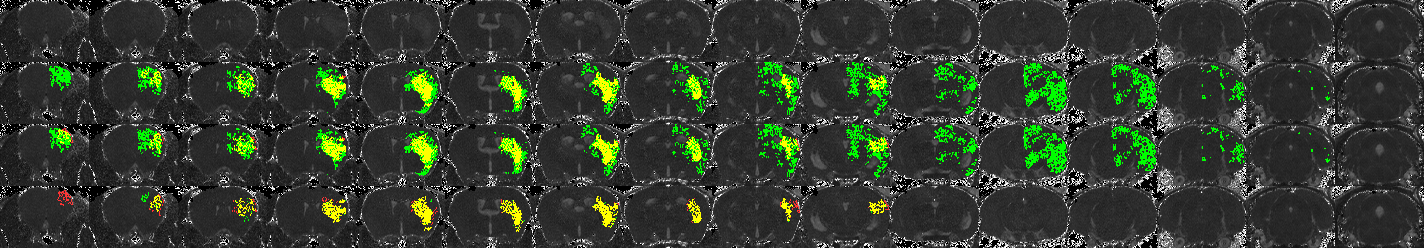

Supplement: Supplementary Material 1 — Segmentation results on the entire validation set. [file DataSheet1.zip › LS_m51_24h_segmentation_results.tif]

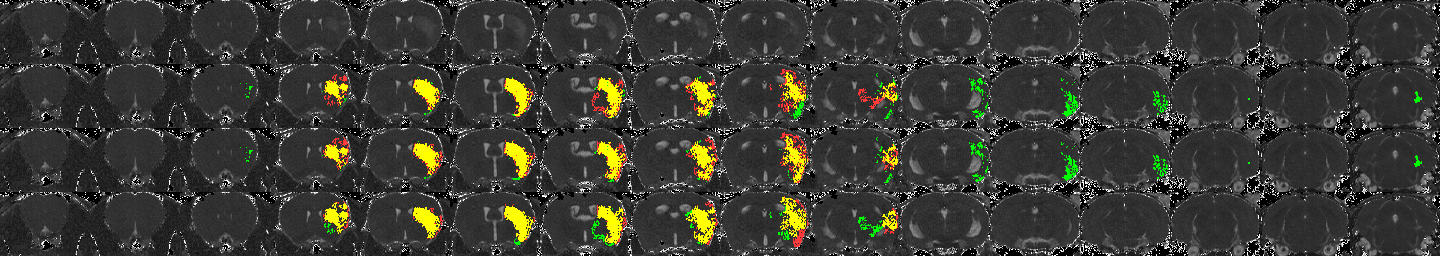

Supplement: Supplementary Material 1 — Segmentation results on the entire validation set. [file DataSheet1.zip › LS_m51_48h_segmentation_results.tif]

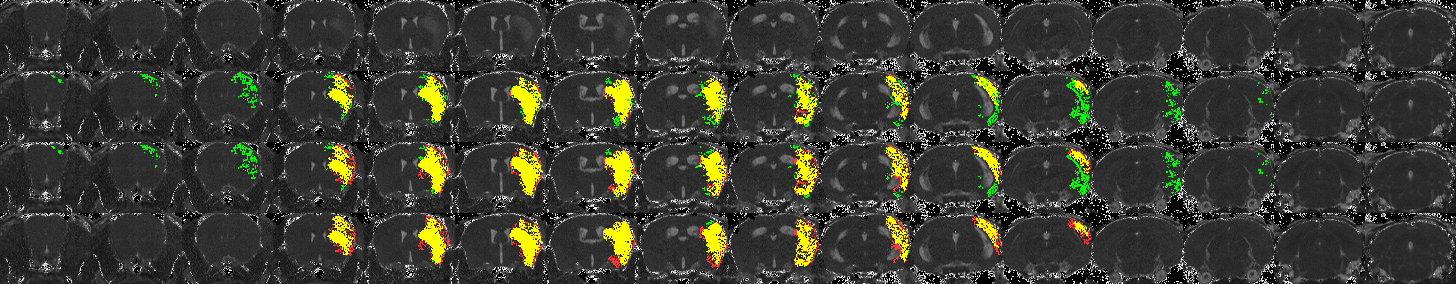

Supplement: Supplementary Material 1 — Segmentation results on the entire validation set. [file DataSheet1.zip › LS_m52_24h_segmentation_results.tif]

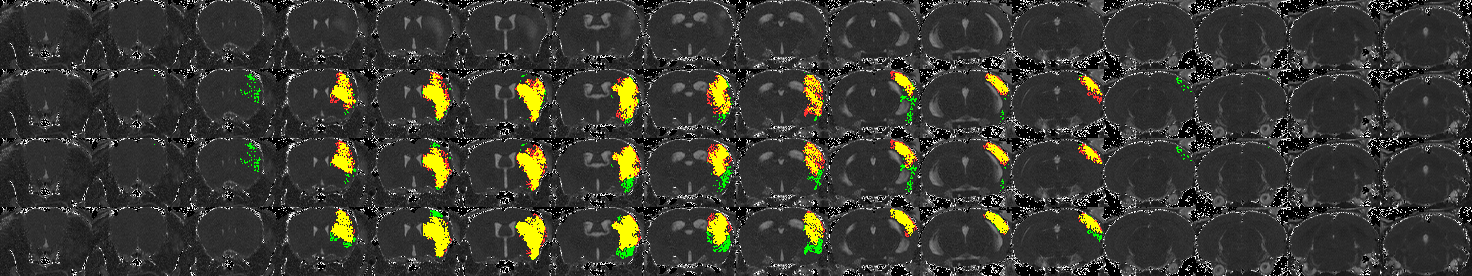

Supplement: Supplementary Material 1 — Segmentation results on the entire validation set. [file DataSheet1.zip › LS_m52_48h_segmentation_results.tif]

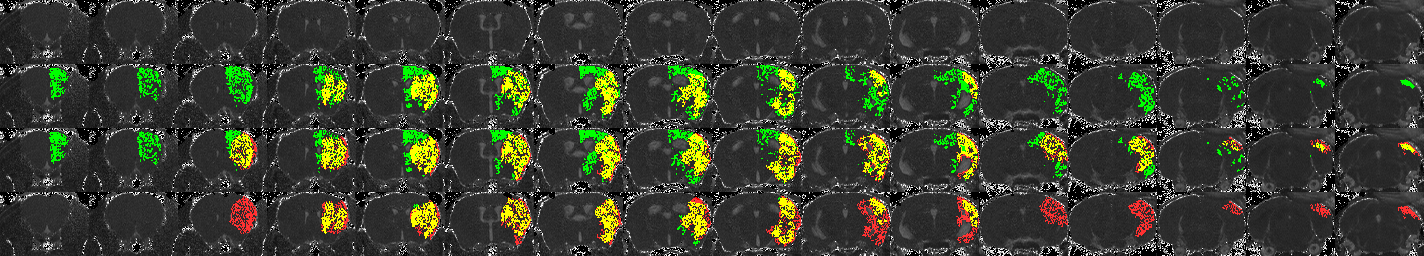

Supplement: Supplementary Material 1 — Segmentation results on the entire validation set. [file DataSheet1.zip › LS_m53_24h_segmentation_results.tif]

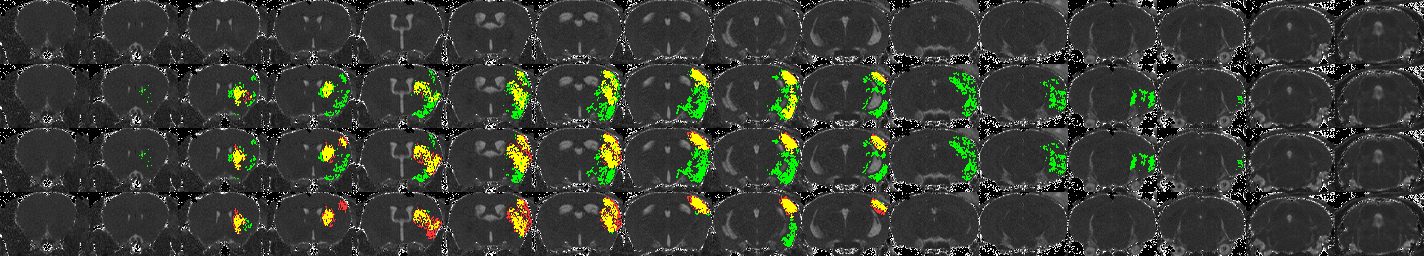

Supplement: Supplementary Material 1 — Segmentation results on the entire validation set. [file DataSheet1.zip › LS_m53_48h_segmentation_results.tif]

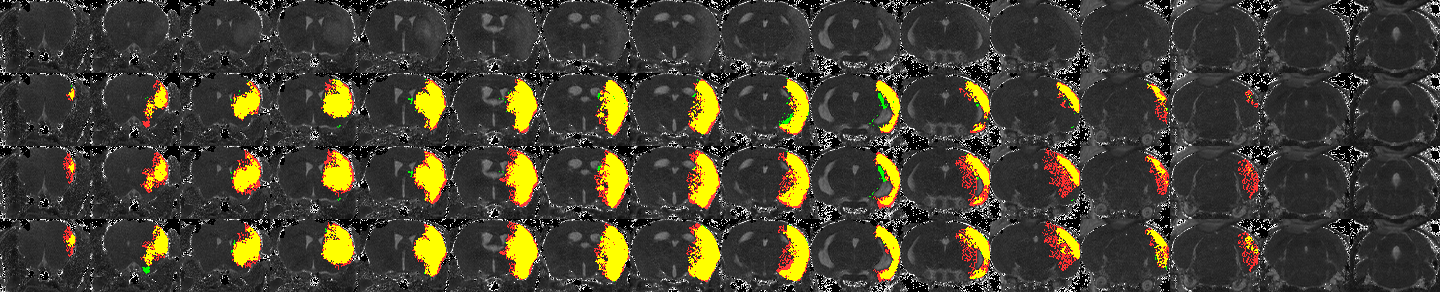

Supplement: Supplementary Material 1 — Segmentation results on the entire validation set. [file DataSheet1.zip › LS_m54_24h_segmentation_results.tif]

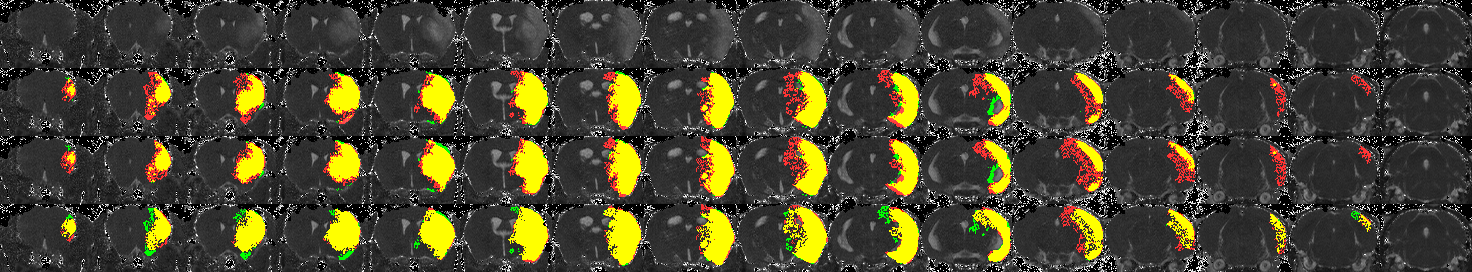

Supplement: Supplementary Material 1 — Segmentation results on the entire validation set. [file DataSheet1.zip › LS_m54_48h_segmentation_results.tif]

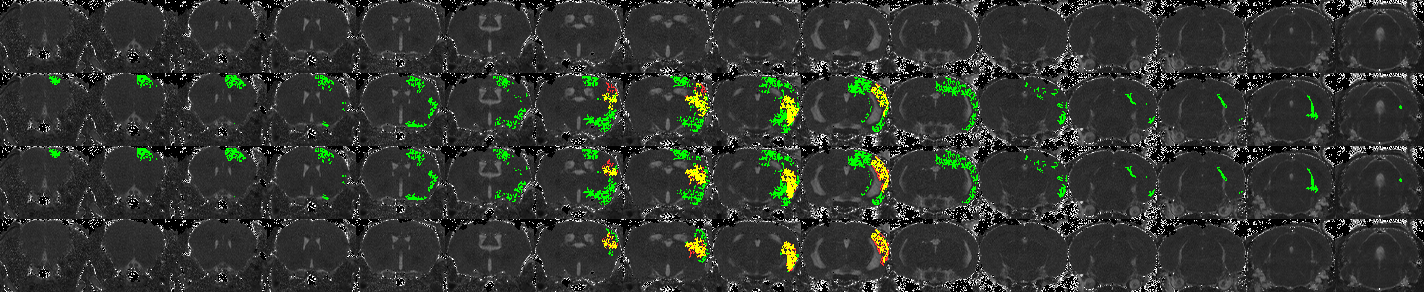

Supplement: Supplementary Material 1 — Segmentation results on the entire validation set. [file DataSheet1.zip › LS_m55_24h_segmentation_results.tif]

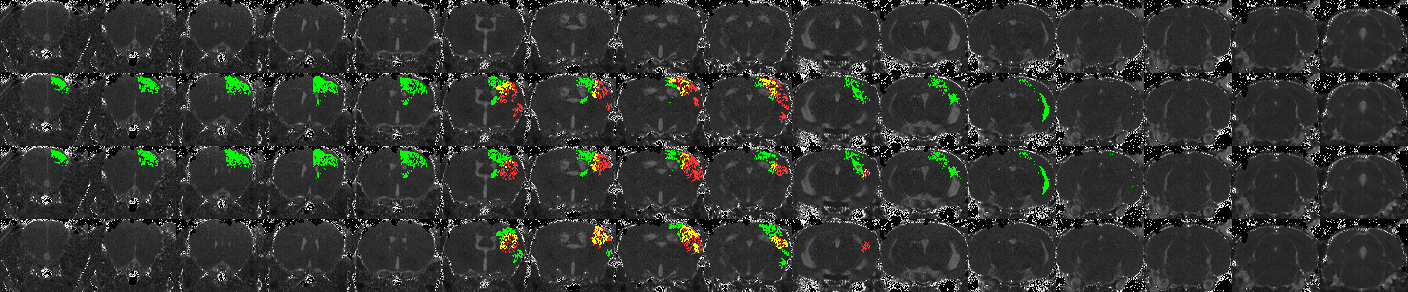

Supplement: Supplementary Material 1 — Segmentation results on the entire validation set. [file DataSheet1.zip › LS_m55_48h_segmentation_results.tif]

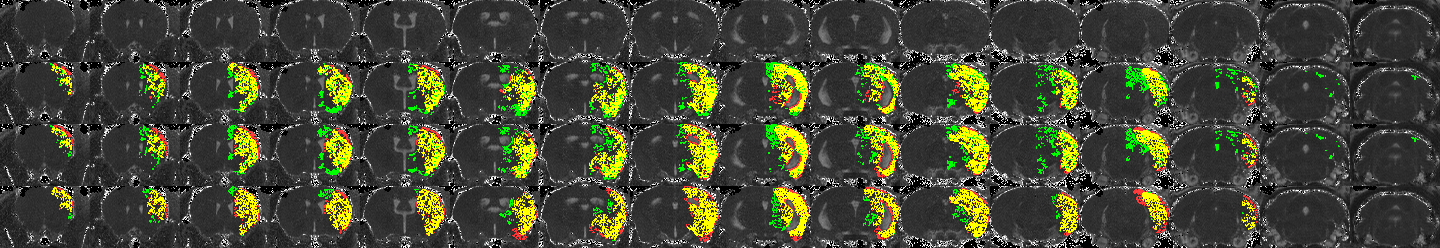

Supplement: Supplementary Material 1 — Segmentation results on the entire validation set. [file DataSheet1.zip › LS_m56_4h_segmentation_results.tif]

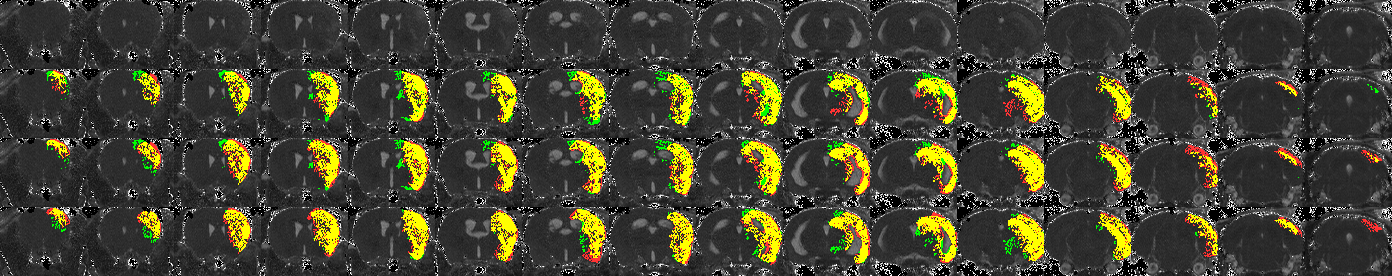

Supplement: Supplementary Material 1 — Segmentation results on the entire validation set. [file DataSheet1.zip › LS_m57_4h_segmentation_results.tif]

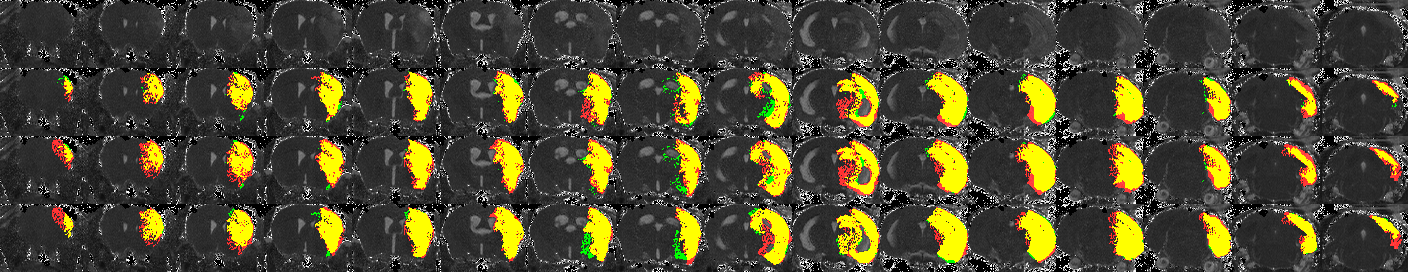

Supplement: Supplementary Material 1 — Segmentation results on the entire validation set. [file DataSheet1.zip › LS_m57_24h_segmentation_results.tif]

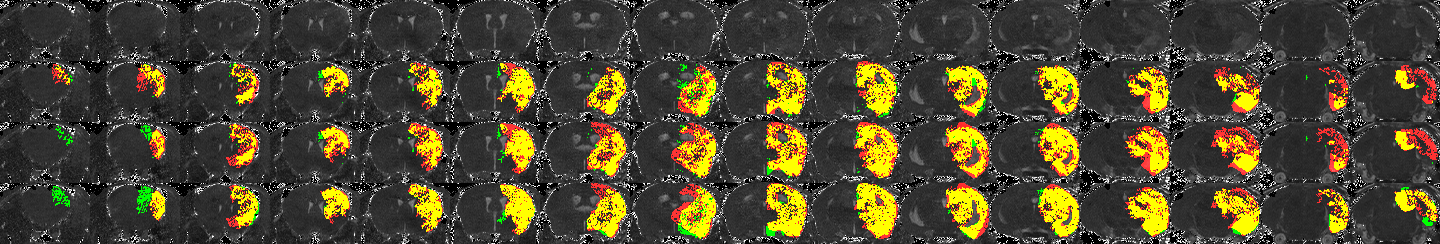

Supplement: Supplementary Material 1 — Segmentation results on the entire validation set. [file DataSheet1.zip › LS_m58_4h_segmentation_results.tif]

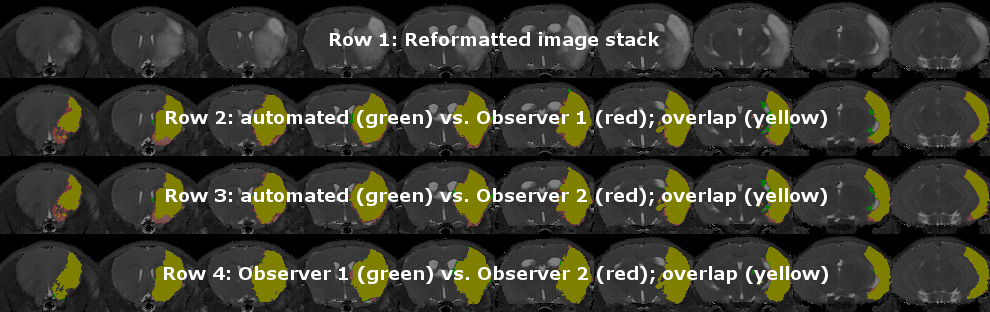

Supplement: Supplementary Material 1 — Segmentation results on the entire validation set. [file DataSheet1.zip › _Legend.tif]
