# Supplementary figures and images for: Automated Ischemic Lesion Segmentation in MRI Mouse Brain Data after Transient Middle Cerebral Artery Occlusion (part 1 of 2)
Source: Front Neuroinform. 2017 Jan 31;11:3. doi: 10.3389/fninf.2017.00003 (PMC5281583; doi:10.3389/fninf.2017.00003)

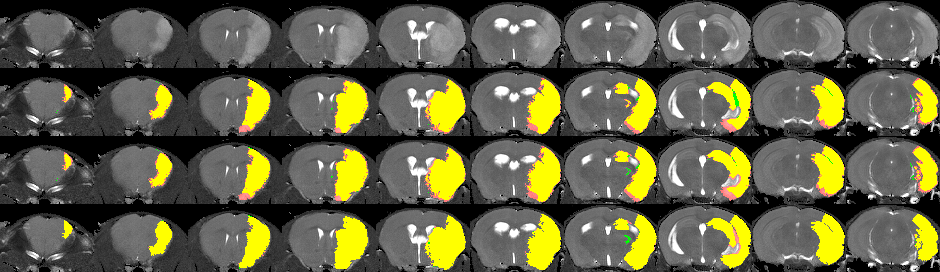

Supplement: Supplementary Material 1 — Segmentation results on the entire validation set. [file DataSheet1.zip › CS1_m1_17h_segmentation_results.tif]

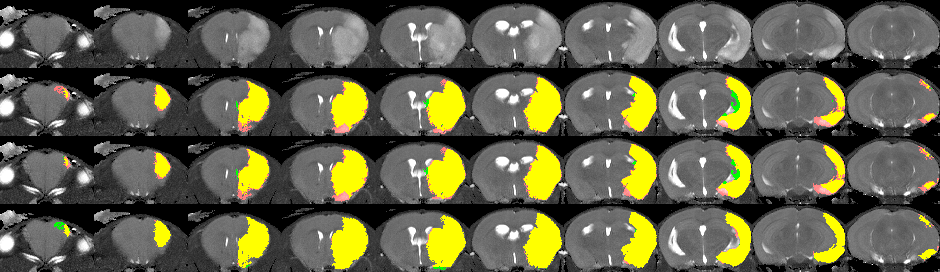

Supplement: Supplementary Material 1 — Segmentation results on the entire validation set. [file DataSheet1.zip › CS1_m2_17h_segmentation_results.tif]

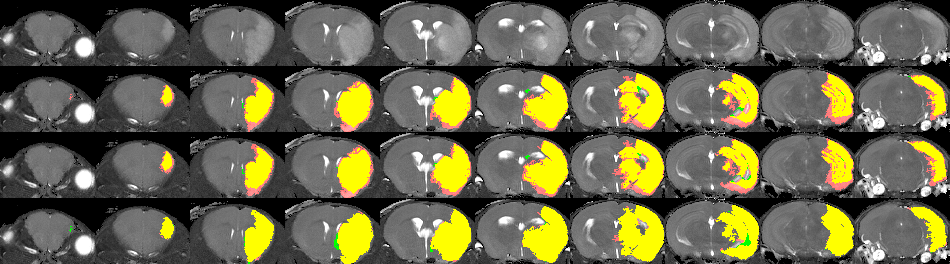

Supplement: Supplementary Material 1 — Segmentation results on the entire validation set. [file DataSheet1.zip › CS1_m3_17h_segmentation_results.tif]

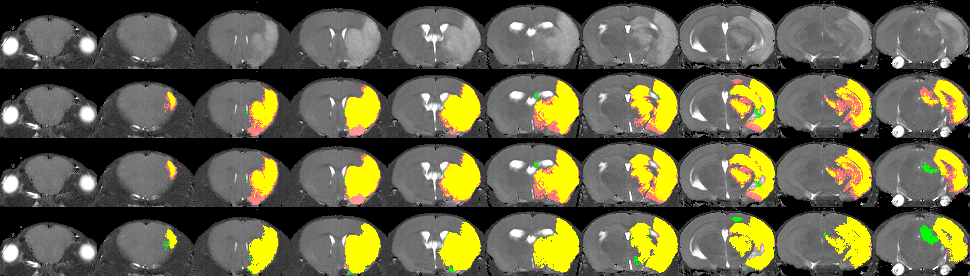

Supplement: Supplementary Material 1 — Segmentation results on the entire validation set. [file DataSheet1.zip › CS1_m4_19h_segmentation_results.tif]

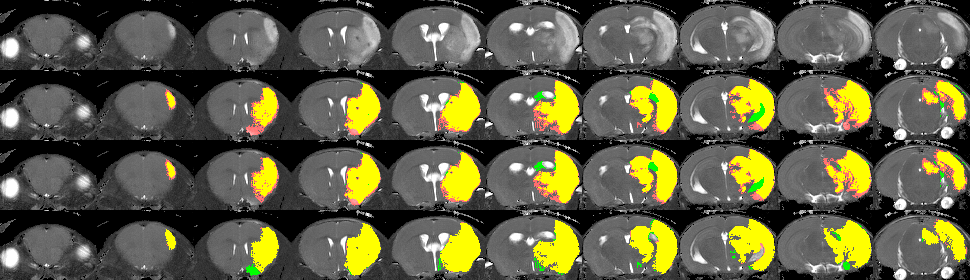

Supplement: Supplementary Material 1 — Segmentation results on the entire validation set. [file DataSheet1.zip › CS1_m4_91h_segmentation_results.tif]

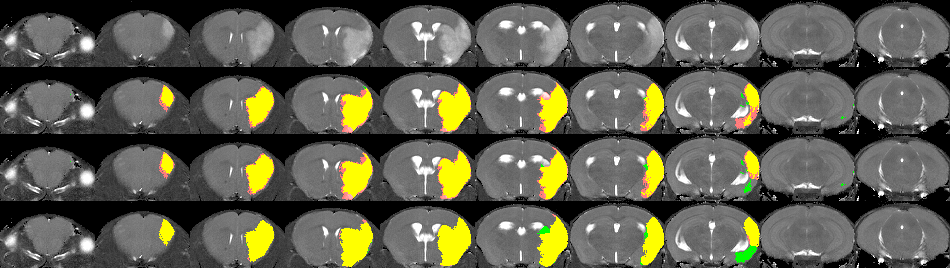

Supplement: Supplementary Material 1 — Segmentation results on the entire validation set. [file DataSheet1.zip › CS1_m5_19h_segmentation_results.tif]

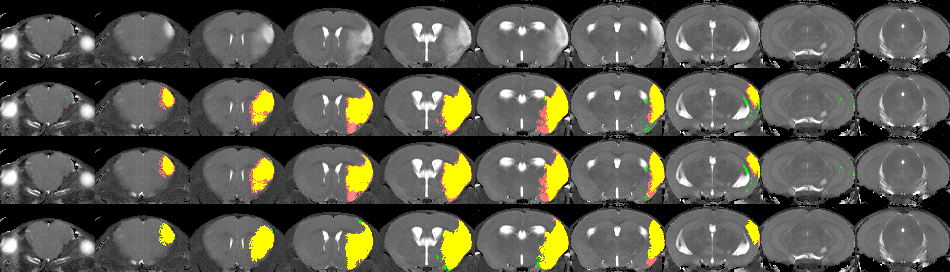

Supplement: Supplementary Material 1 — Segmentation results on the entire validation set. [file DataSheet1.zip › CS1_m5_91h_segmentation_results.tif]

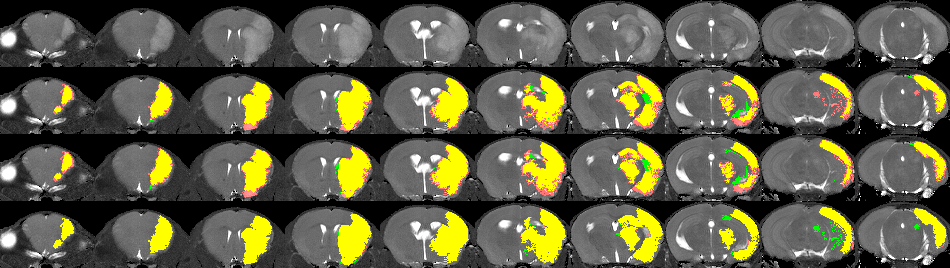

Supplement: Supplementary Material 1 — Segmentation results on the entire validation set. [file DataSheet1.zip › CS1_m6_19h_segmentation_results.tif]

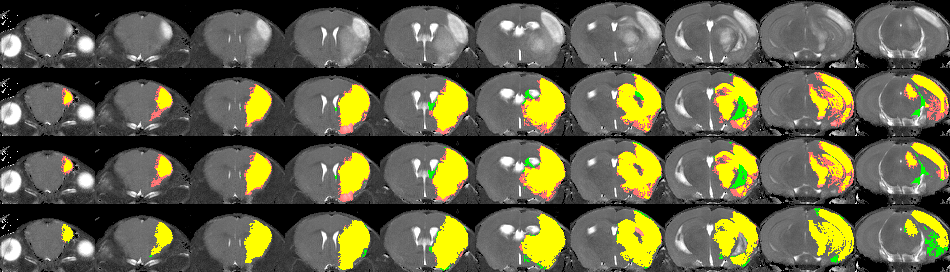

Supplement: Supplementary Material 1 — Segmentation results on the entire validation set. [file DataSheet1.zip › CS1_m6_91h_segmentation_results.tif]

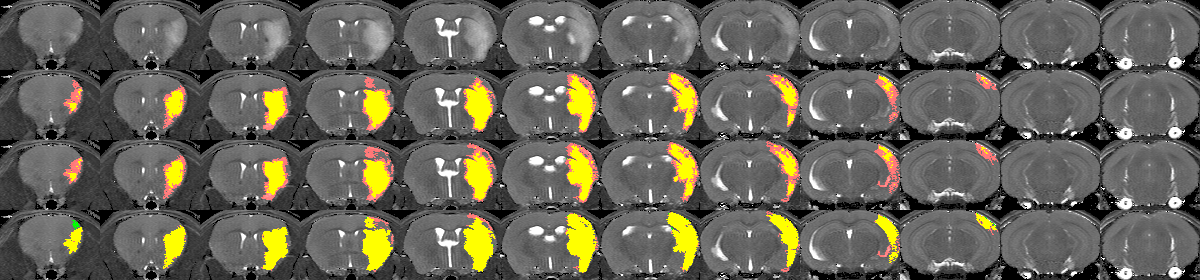

Supplement: Supplementary Material 1 — Segmentation results on the entire validation set. [file DataSheet1.zip › CS2_m1_2d_segmentation_results.tif]

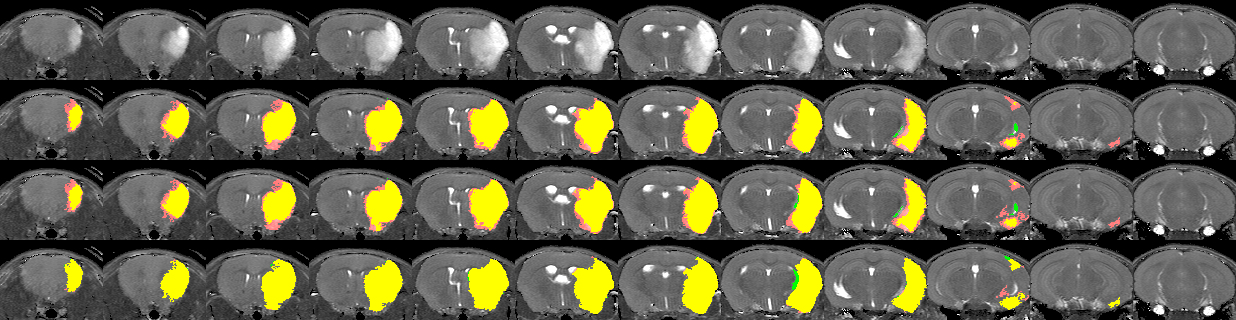

Supplement: Supplementary Material 1 — Segmentation results on the entire validation set. [file DataSheet1.zip › CS2_m2_2d_segmentation_results.tif]

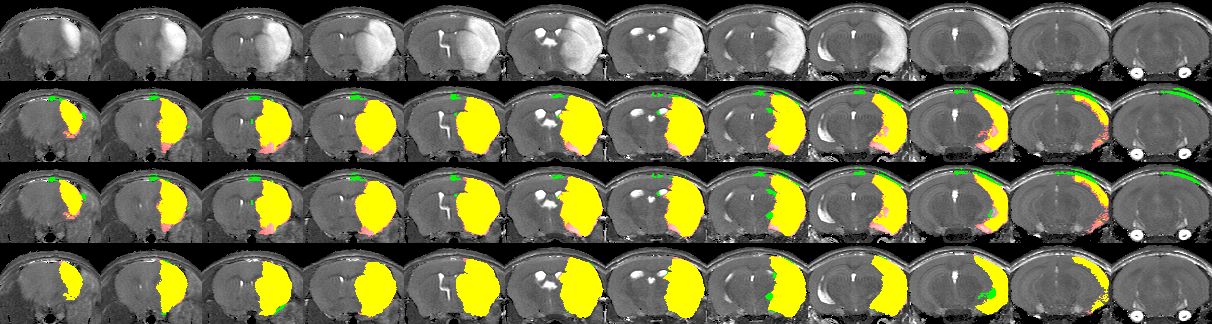

Supplement: Supplementary Material 1 — Segmentation results on the entire validation set. [file DataSheet1.zip › CS2_m3_2d_segmentation_results.tif]

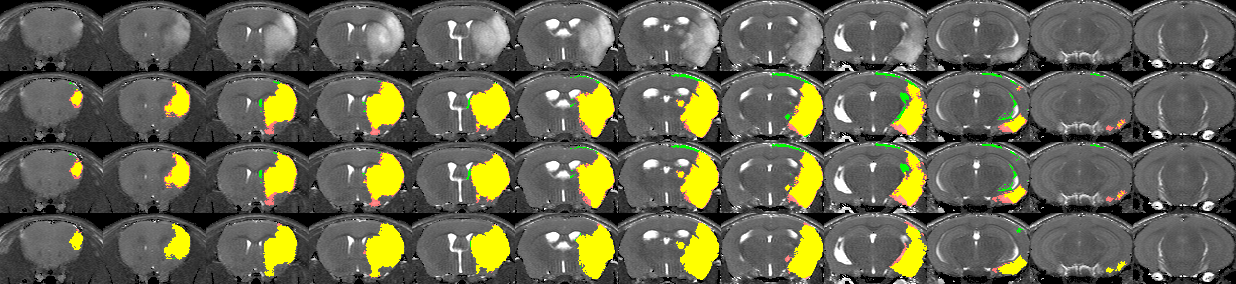

Supplement: Supplementary Material 1 — Segmentation results on the entire validation set. [file DataSheet1.zip › CS2_m4_2d_segmentation_results.tif]

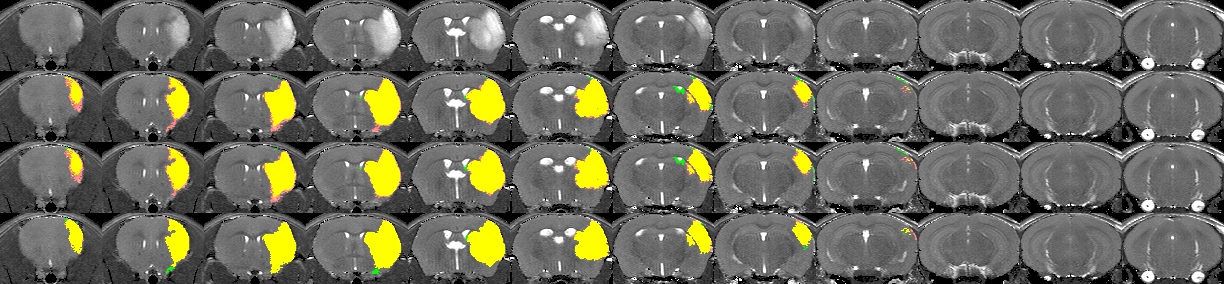

Supplement: Supplementary Material 1 — Segmentation results on the entire validation set. [file DataSheet1.zip › CS2_m5_2d_segmentation_results.tif]

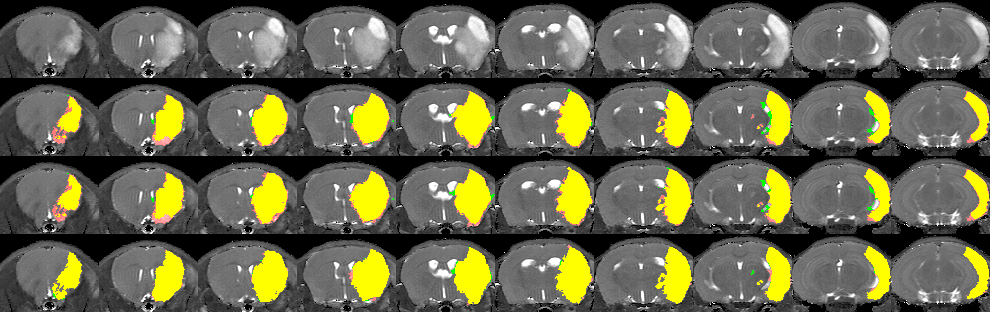

Supplement: Supplementary Material 1 — Segmentation results on the entire validation set. [file DataSheet1.zip › CS2_m6_2d_segmentation_results.tif]

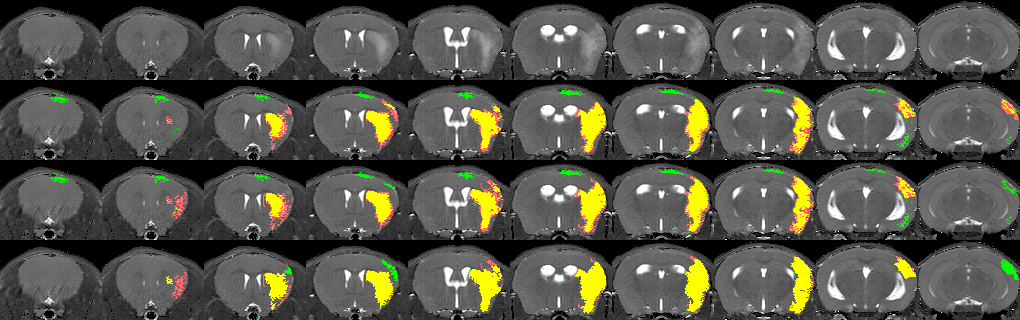

Supplement: Supplementary Material 1 — Segmentation results on the entire validation set. [file DataSheet1.zip › CS2_m7_2d_segmentation_results.tif]

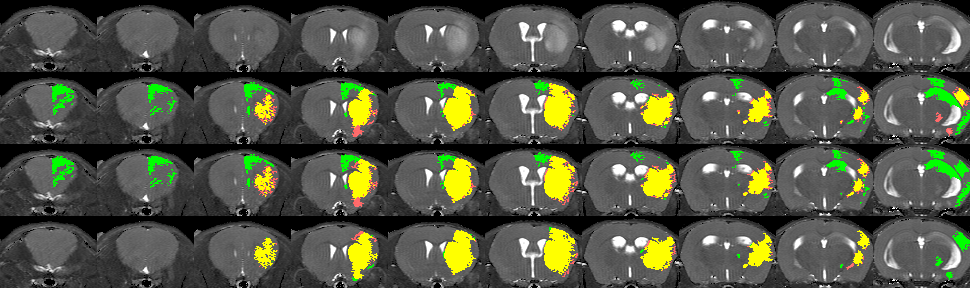

Supplement: Supplementary Material 1 — Segmentation results on the entire validation set. [file DataSheet1.zip › CS2_m8_2d_segmentation_results.tif]

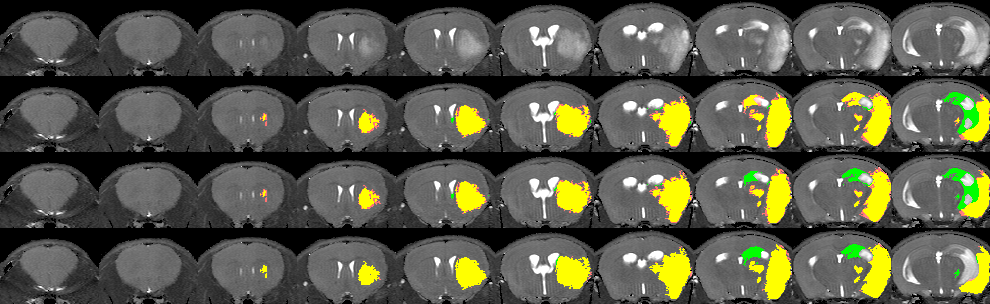

Supplement: Supplementary Material 1 — Segmentation results on the entire validation set. [file DataSheet1.zip › CS2_m9_2d_segmentation_results.tif]

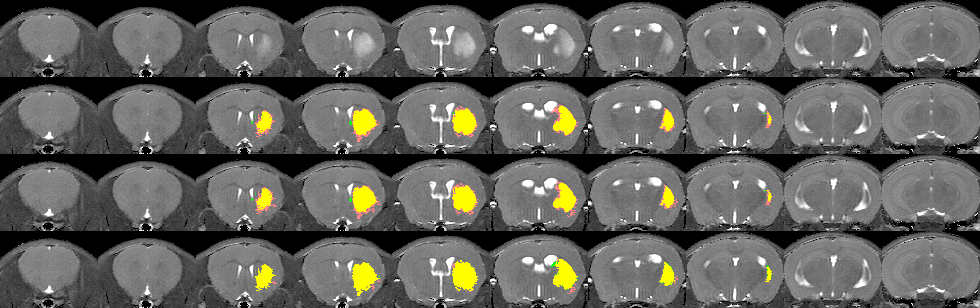

Supplement: Supplementary Material 1 — Segmentation results on the entire validation set. [file DataSheet1.zip › CS2_m10_2d_segmentation_results.tif]

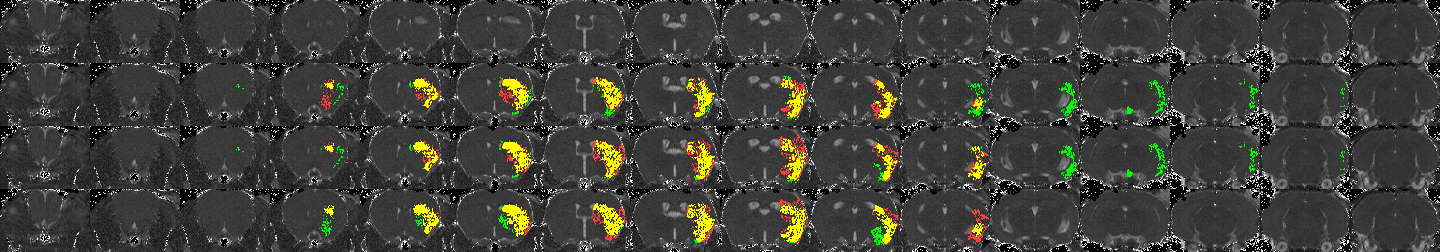

Supplement: Supplementary Material 1 — Segmentation results on the entire validation set. [file DataSheet1.zip › LS_m1_4h_segmentation_results.tif]

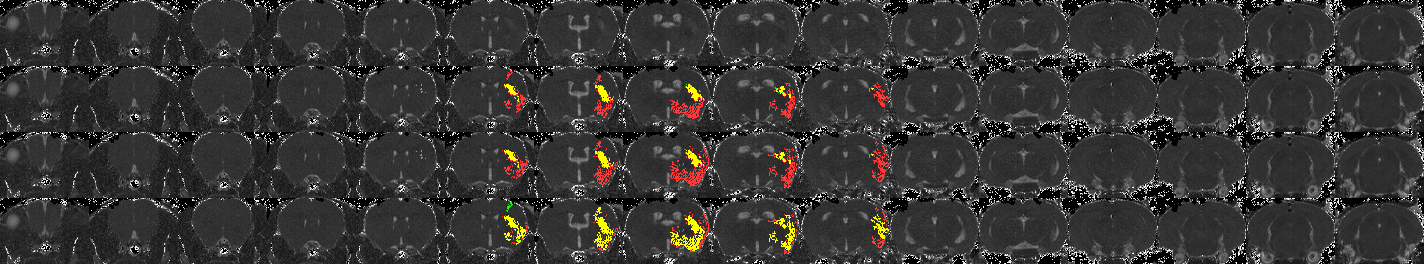

Supplement: Supplementary Material 1 — Segmentation results on the entire validation set. [file DataSheet1.zip › LS_m1_8d_segmentation_results.tif]

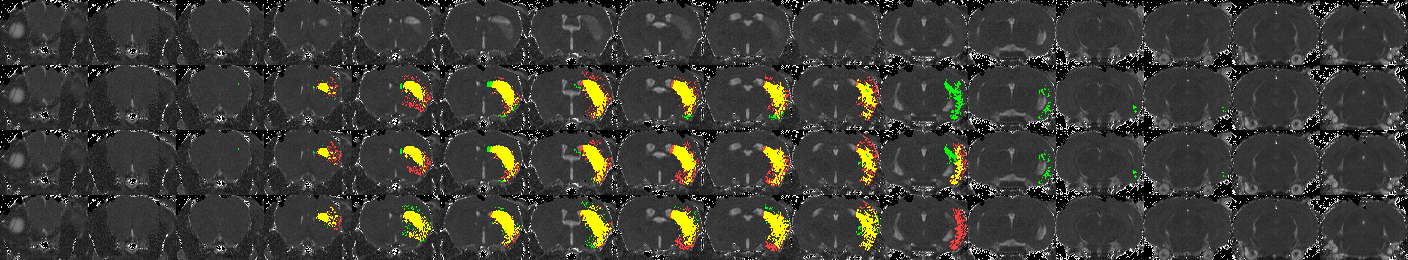

Supplement: Supplementary Material 1 — Segmentation results on the entire validation set. [file DataSheet1.zip › LS_m1_24h_segmentation_results.tif]

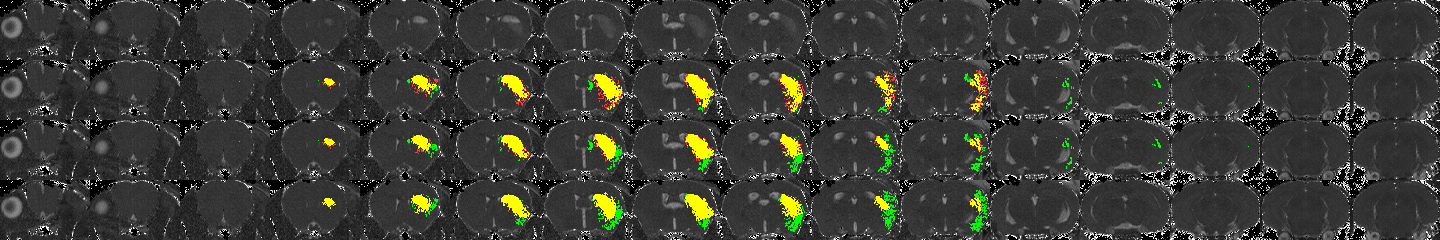

Supplement: Supplementary Material 1 — Segmentation results on the entire validation set. [file DataSheet1.zip › LS_m1_48h_segmentation_results.tif]

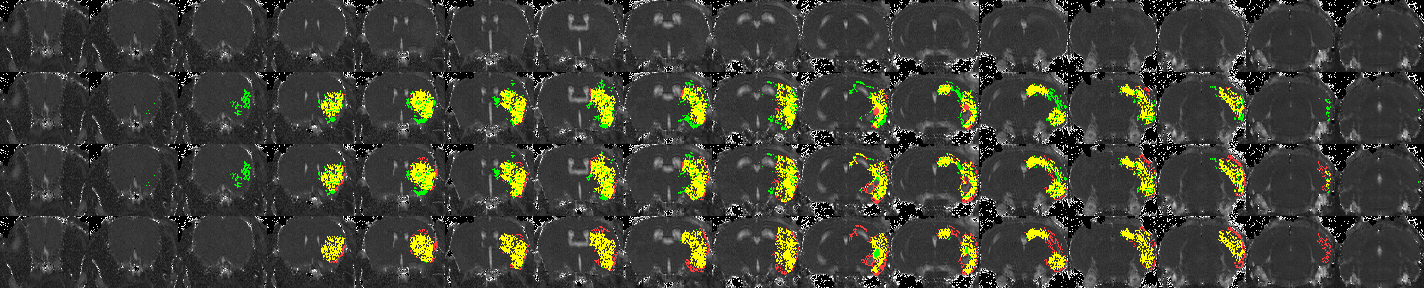

Supplement: Supplementary Material 1 — Segmentation results on the entire validation set. [file DataSheet1.zip › LS_m2_4h_segmentation_results.tif]

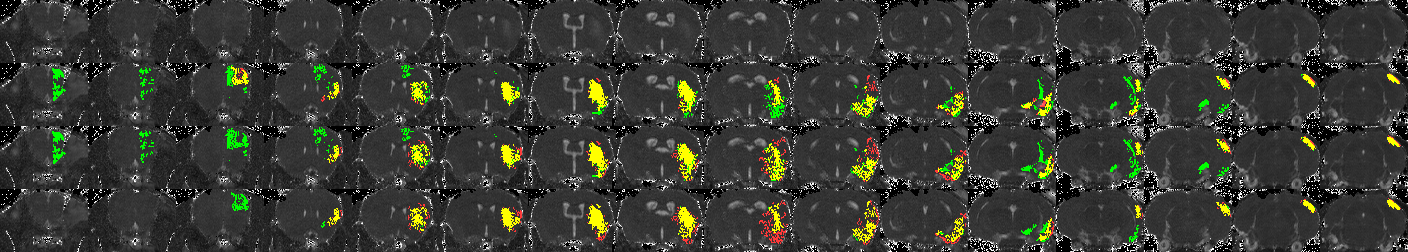

Supplement: Supplementary Material 1 — Segmentation results on the entire validation set. [file DataSheet1.zip › LS_m2_8d_segmentation_results.tif]

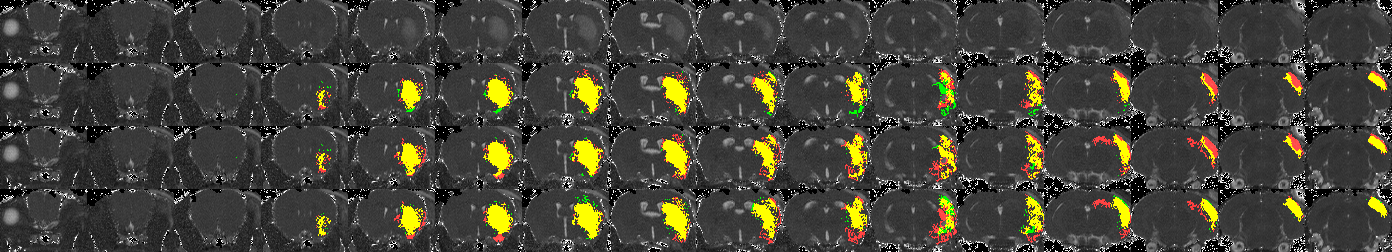

Supplement: Supplementary Material 1 — Segmentation results on the entire validation set. [file DataSheet1.zip › LS_m2_24h_segmentation_results.tif]

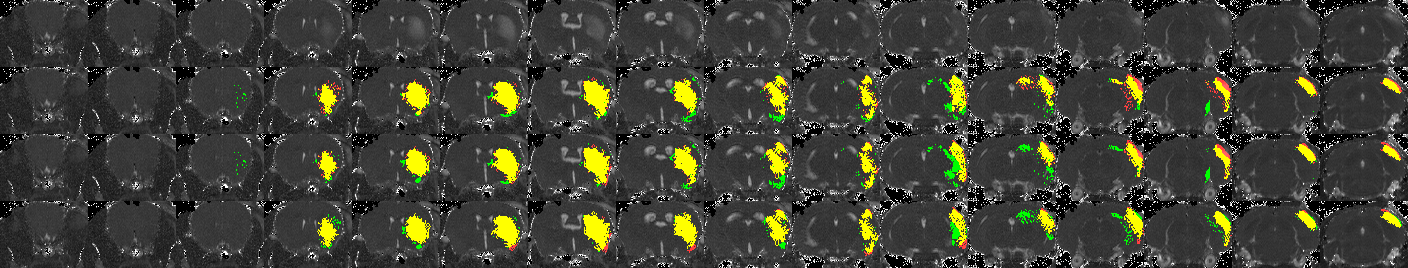

Supplement: Supplementary Material 1 — Segmentation results on the entire validation set. [file DataSheet1.zip › LS_m2_48h_segmentation_results.tif]

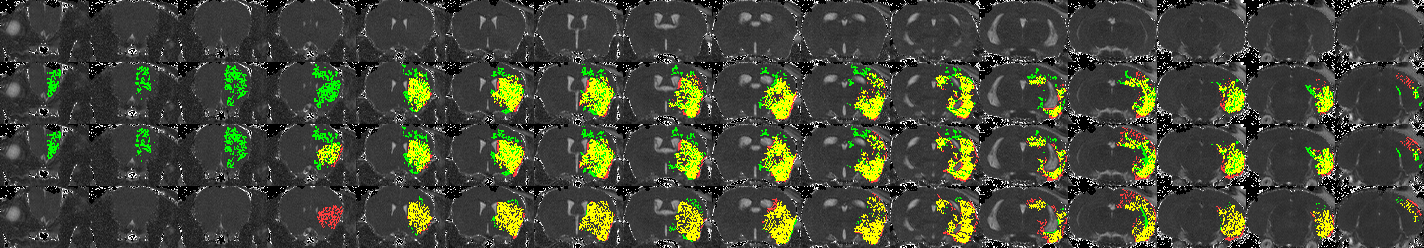

Supplement: Supplementary Material 1 — Segmentation results on the entire validation set. [file DataSheet1.zip › LS_m3_4h_segmentation_results.tif]

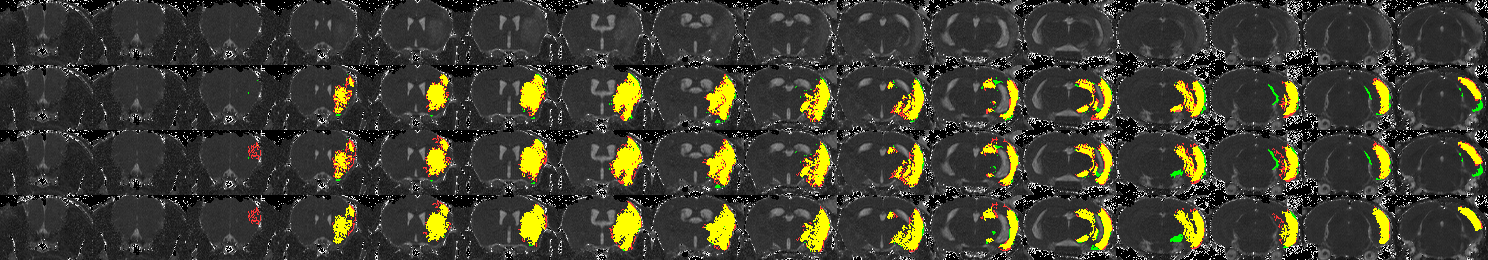

Supplement: Supplementary Material 1 — Segmentation results on the entire validation set. [file DataSheet1.zip › LS_m3_8d_segmentation_results.tif]

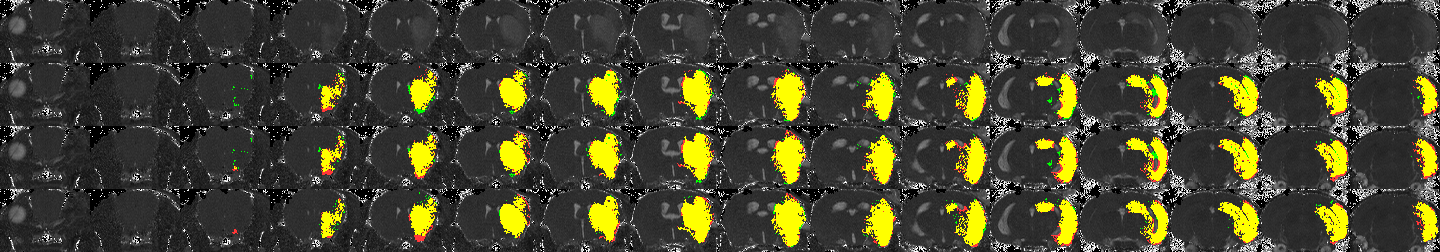

Supplement: Supplementary Material 1 — Segmentation results on the entire validation set. [file DataSheet1.zip › LS_m3_24h_segmentation_results.tif]

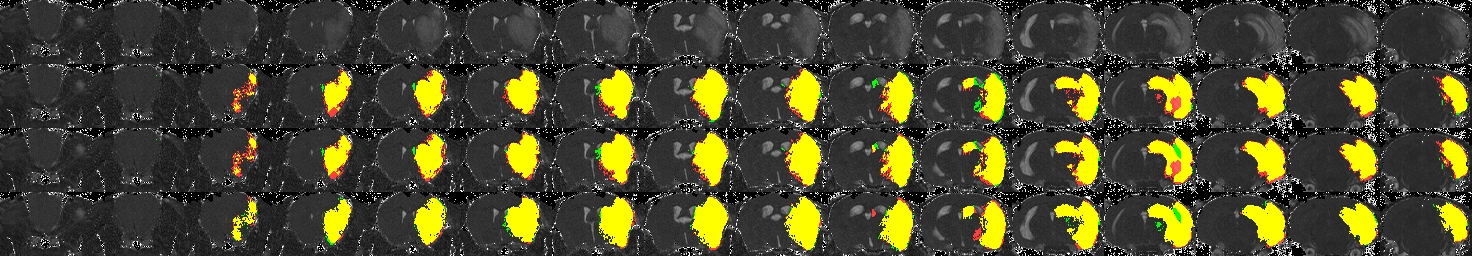

Supplement: Supplementary Material 1 — Segmentation results on the entire validation set. [file DataSheet1.zip › LS_m3_48h_segmentation_results.tif]

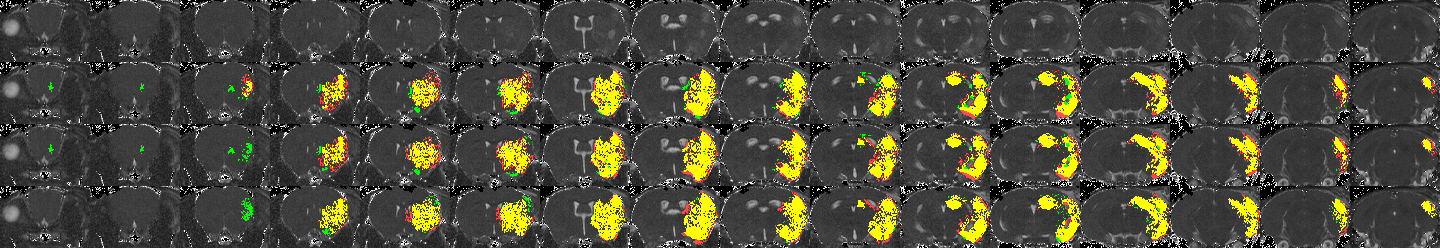

Supplement: Supplementary Material 1 — Segmentation results on the entire validation set. [file DataSheet1.zip › LS_m4_4h_segmentation_results.tif]

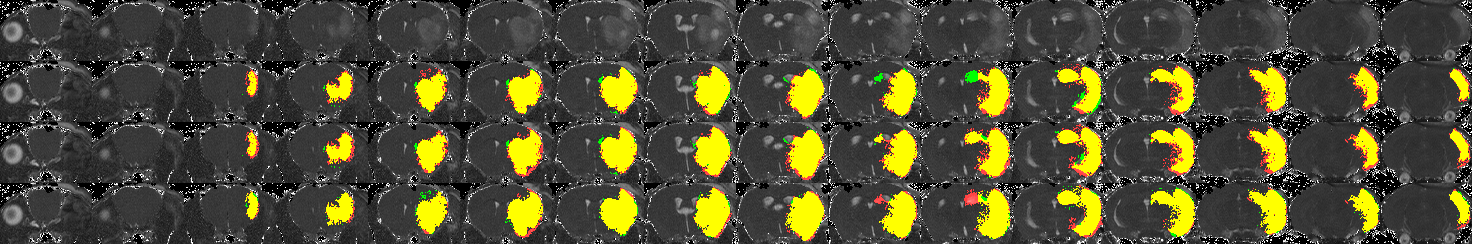

Supplement: Supplementary Material 1 — Segmentation results on the entire validation set. [file DataSheet1.zip › LS_m4_24h_segmentation_results.tif]

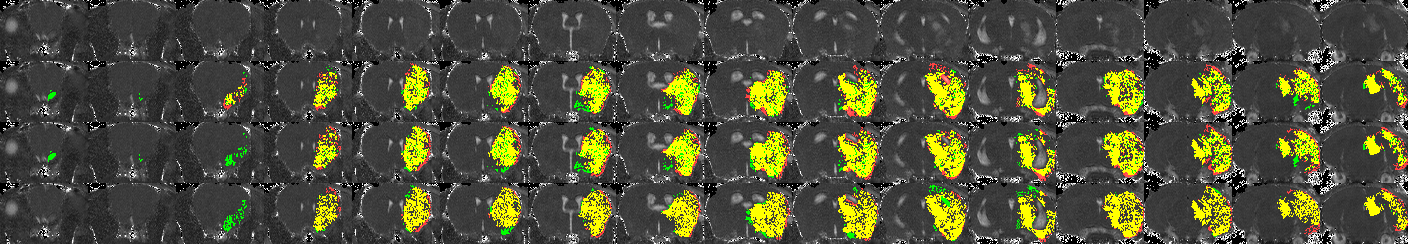

Supplement: Supplementary Material 1 — Segmentation results on the entire validation set. [file DataSheet1.zip › LS_m5_4h_segmentation_results.tif]

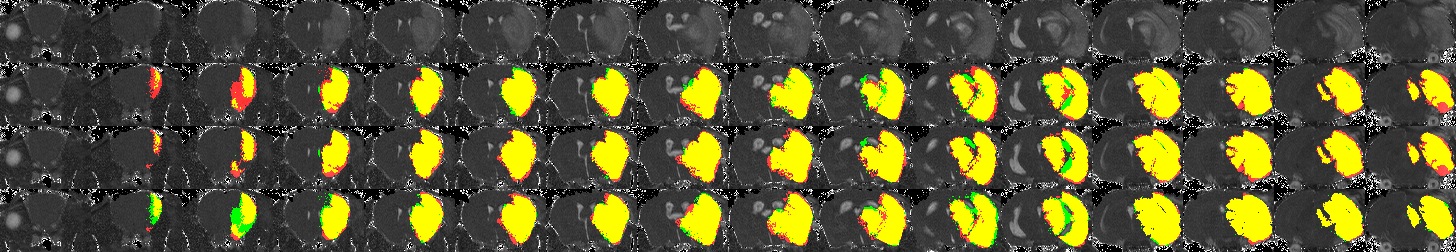

Supplement: Supplementary Material 1 — Segmentation results on the entire validation set. [file DataSheet1.zip › LS_m5_24h_segmentation_results.tif]

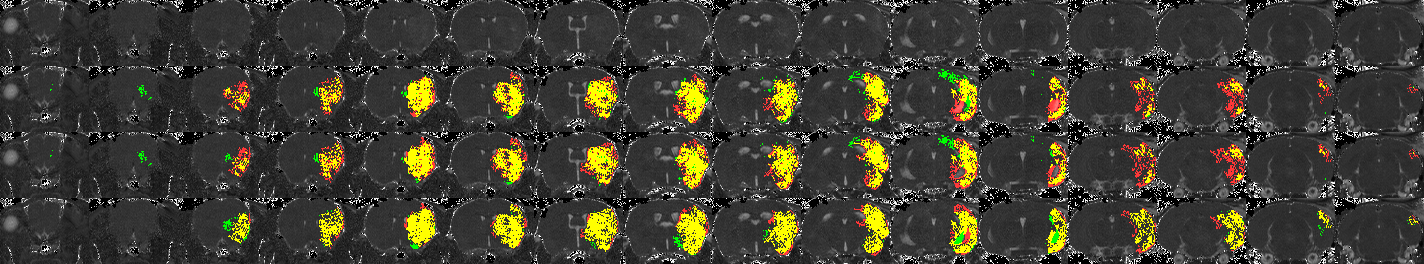

Supplement: Supplementary Material 1 — Segmentation results on the entire validation set. [file DataSheet1.zip › LS_m6_4h_segmentation_results.tif]

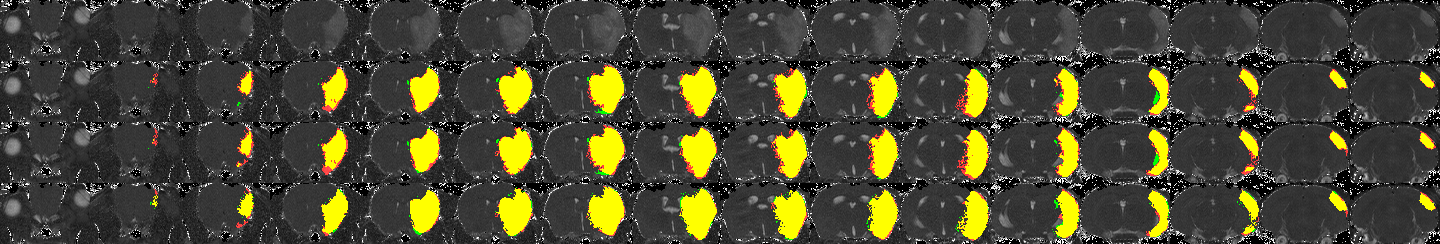

Supplement: Supplementary Material 1 — Segmentation results on the entire validation set. [file DataSheet1.zip › LS_m6_24h_segmentation_results.tif]

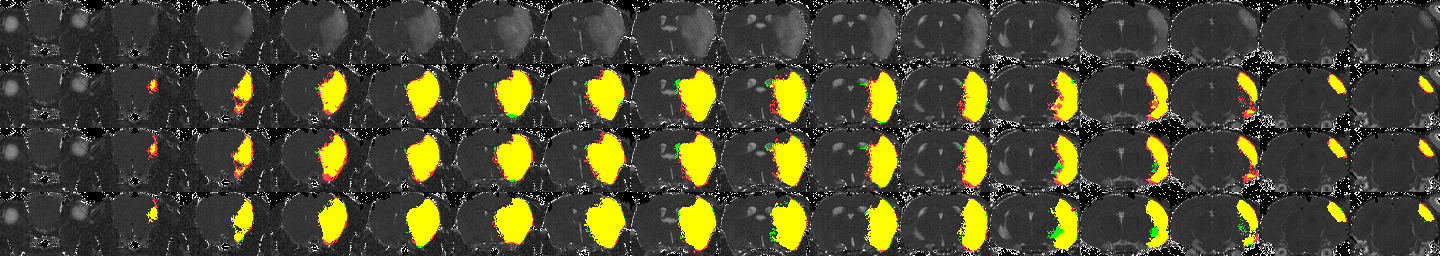

Supplement: Supplementary Material 1 — Segmentation results on the entire validation set. [file DataSheet1.zip › LS_m6_48h_segmentation_results.tif]

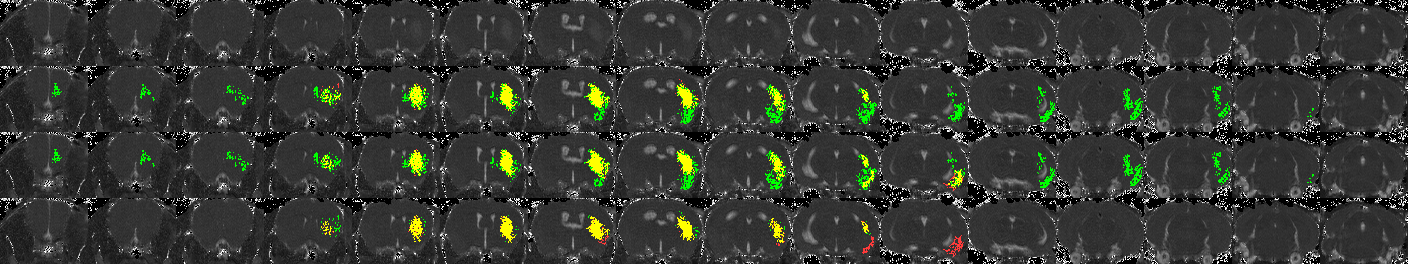

Supplement: Supplementary Material 1 — Segmentation results on the entire validation set. [file DataSheet1.zip › LS_m7_24h_segmentation_results.tif]

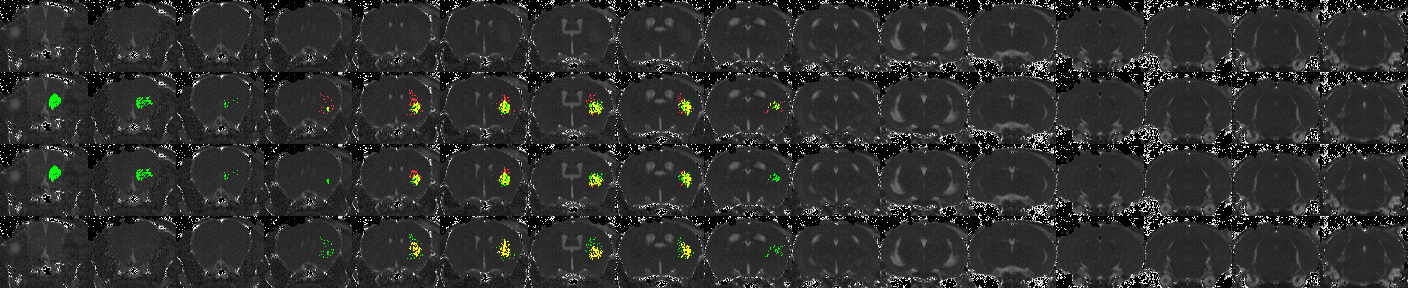

Supplement: Supplementary Material 1 — Segmentation results on the entire validation set. [file DataSheet1.zip › LS_m7_48h_segmentation_results.tif]

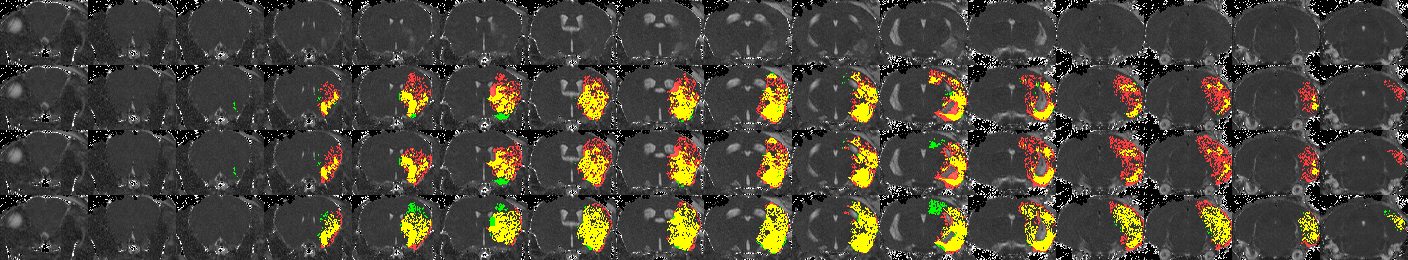

Supplement: Supplementary Material 1 — Segmentation results on the entire validation set. [file DataSheet1.zip › LS_m8_4h_segmentation_results.tif]

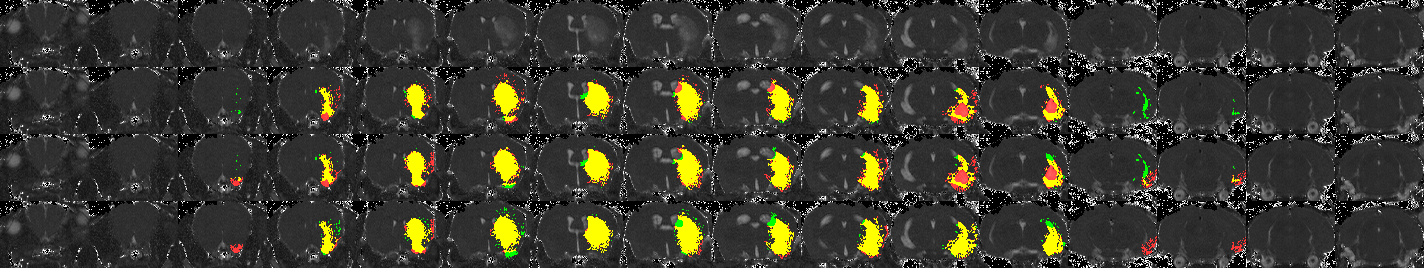

Supplement: Supplementary Material 1 — Segmentation results on the entire validation set. [file DataSheet1.zip › LS_m8_24h_segmentation_results.tif]

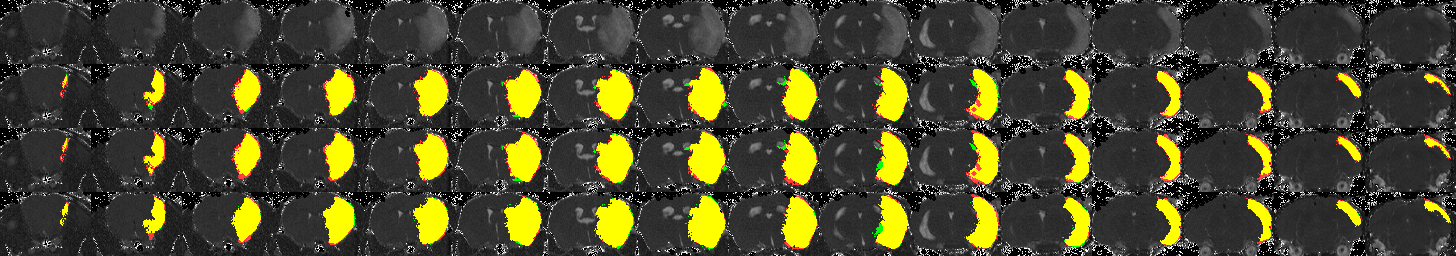

Supplement: Supplementary Material 1 — Segmentation results on the entire validation set. [file DataSheet1.zip › LS_m9_48h_segmentation_results.tif]

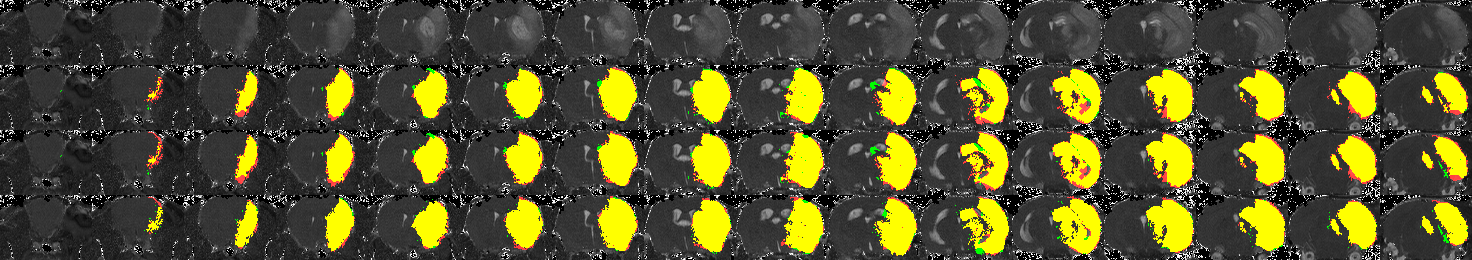

Supplement: Supplementary Material 1 — Segmentation results on the entire validation set. [file DataSheet1.zip › LS_m10_24h_segmentation_results.tif]

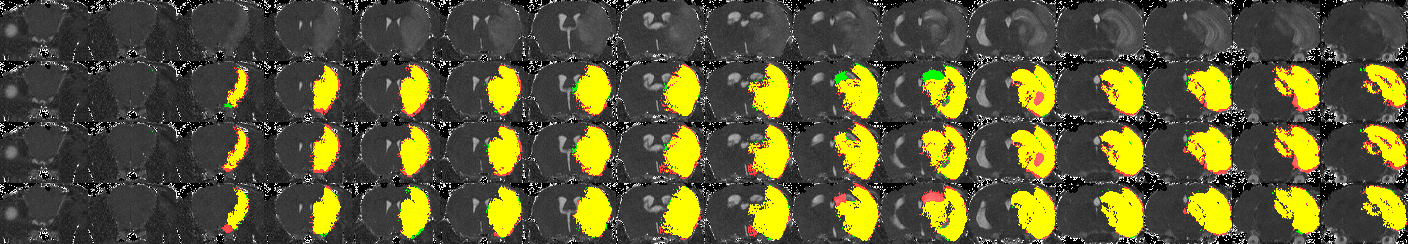

Supplement: Supplementary Material 1 — Segmentation results on the entire validation set. [file DataSheet1.zip › LS_m11_24h_segmentation_results.tif]

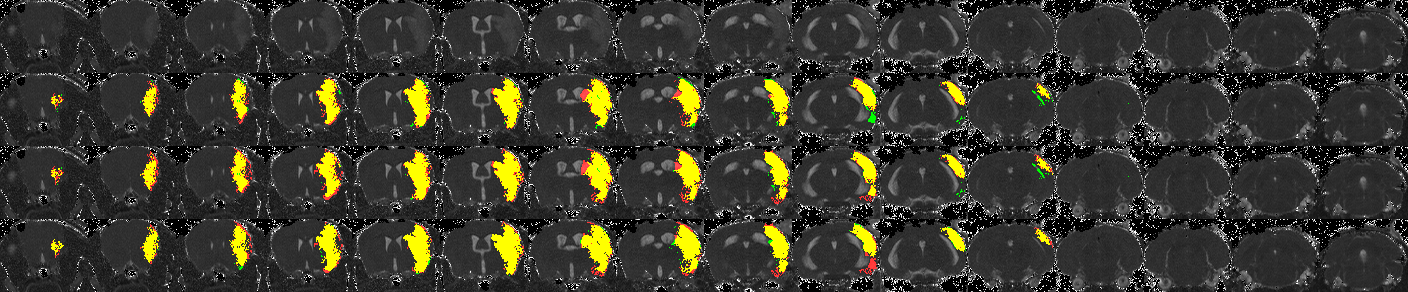

Supplement: Supplementary Material 1 — Segmentation results on the entire validation set. [file DataSheet1.zip › LS_m12_24h_segmentation_results.tif]

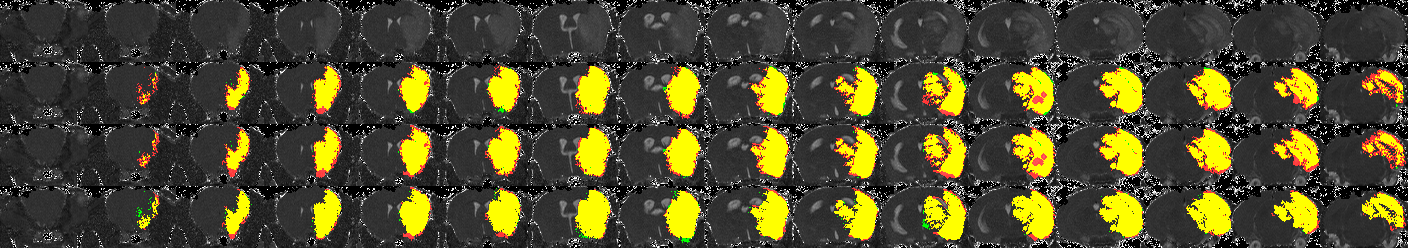

Supplement: Supplementary Material 1 — Segmentation results on the entire validation set. [file DataSheet1.zip › LS_m13_24h_segmentation_results.tif]

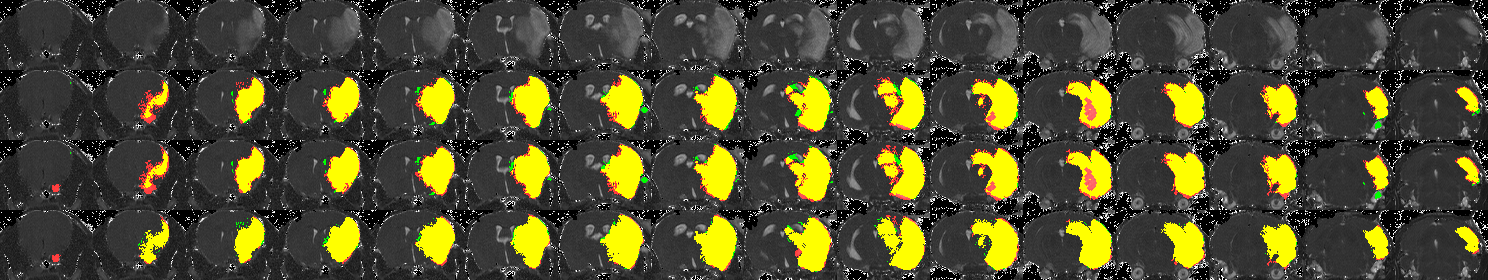

Supplement: Supplementary Material 1 — Segmentation results on the entire validation set. [file DataSheet1.zip › LS_m14_48h_segmentation_results.tif]

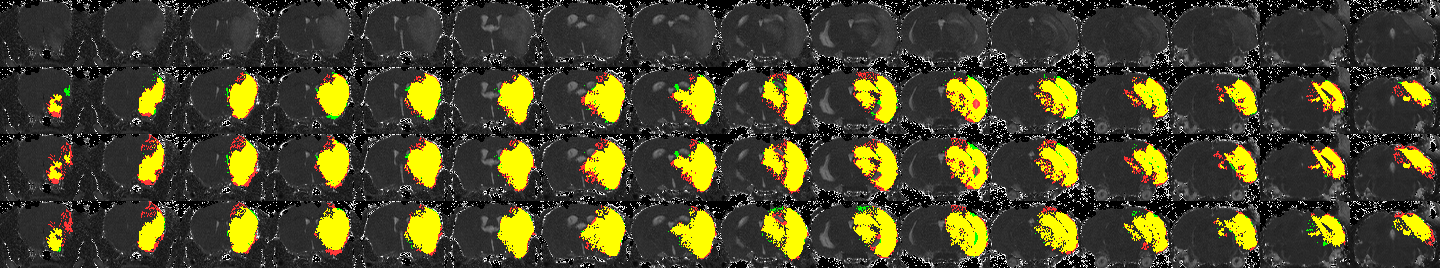

Supplement: Supplementary Material 1 — Segmentation results on the entire validation set. [file DataSheet1.zip › LS_m15_24h_segmentation_results.tif]

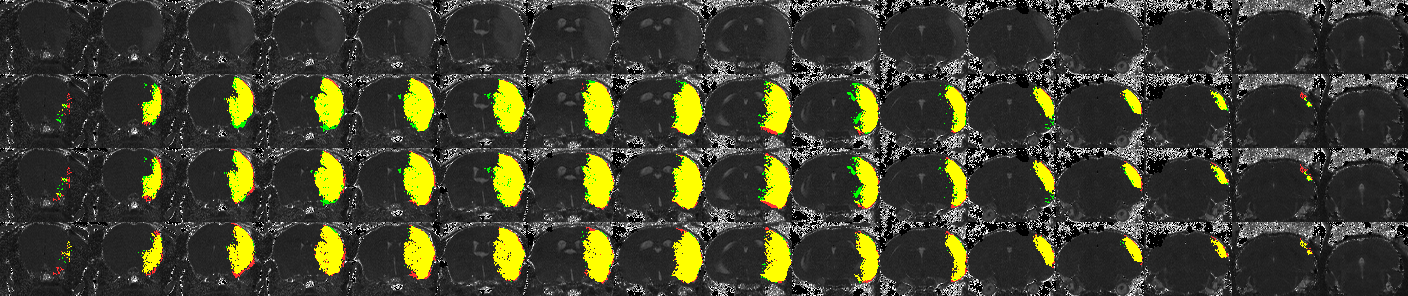

Supplement: Supplementary Material 1 — Segmentation results on the entire validation set. [file DataSheet1.zip › LS_m16_24h_segmentation_results.tif]

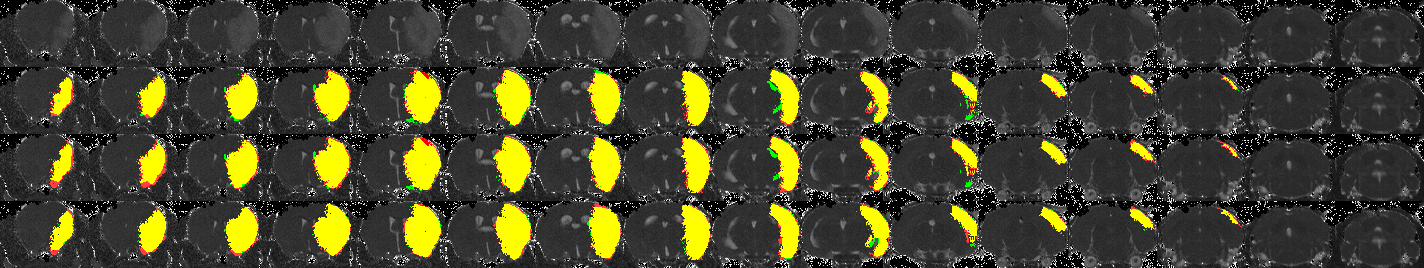

Supplement: Supplementary Material 1 — Segmentation results on the entire validation set. [file DataSheet1.zip › LS_m17_24h_segmentation_results.tif]

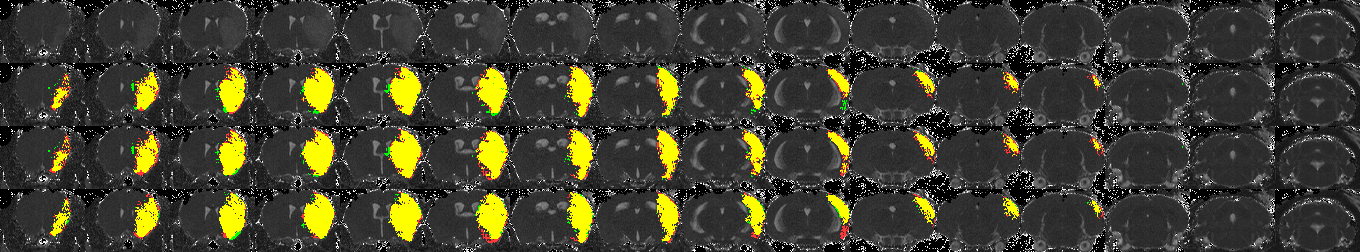

Supplement: Supplementary Material 1 — Segmentation results on the entire validation set. [file DataSheet1.zip › LS_m18_24h_segmentation_results.tif]

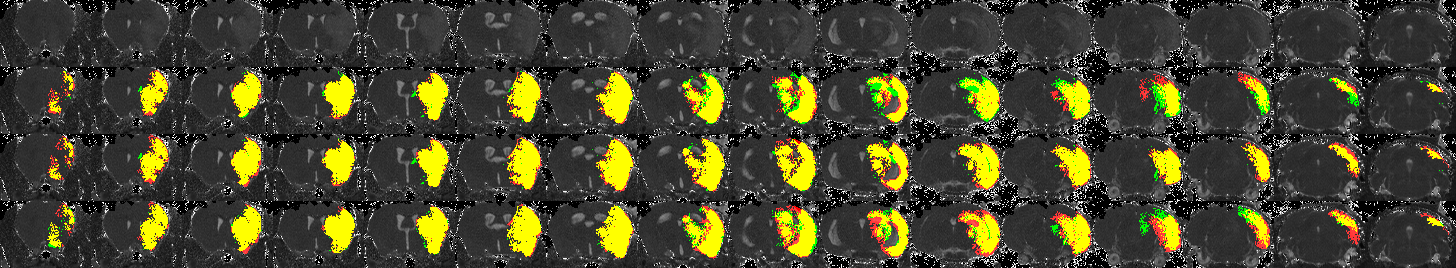

Supplement: Supplementary Material 1 — Segmentation results on the entire validation set. [file DataSheet1.zip › LS_m19_24h_segmentation_results.tif]

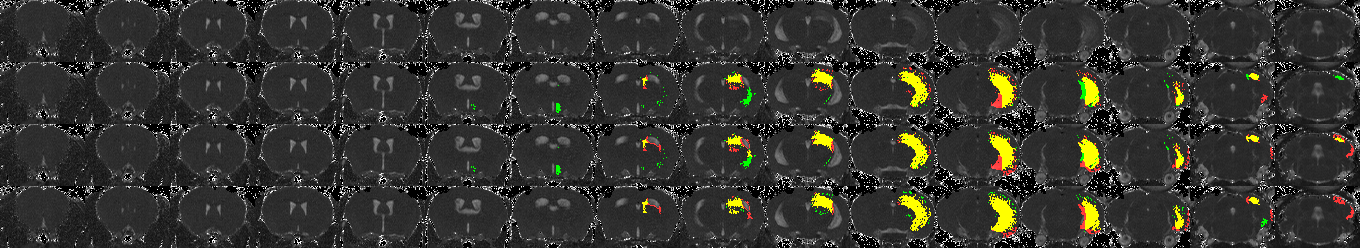

Supplement: Supplementary Material 1 — Segmentation results on the entire validation set. [file DataSheet1.zip › LS_m20_24h_segmentation_results.tif]

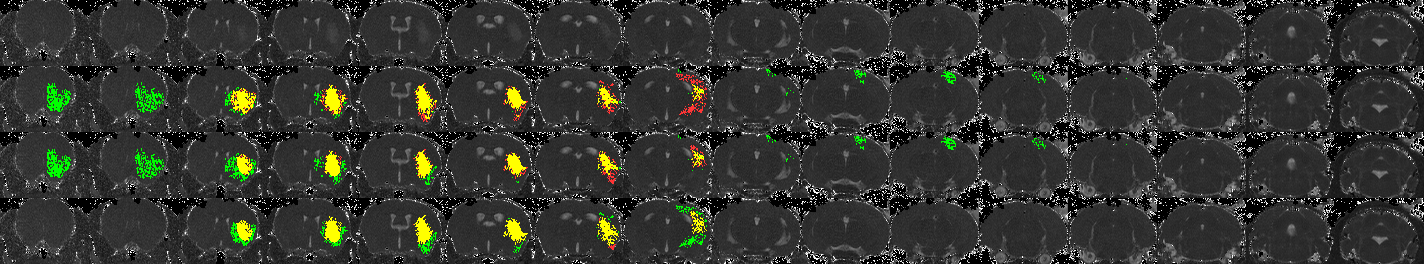

Supplement: Supplementary Material 1 — Segmentation results on the entire validation set. [file DataSheet1.zip › LS_m21_24h_segmentation_results.tif]

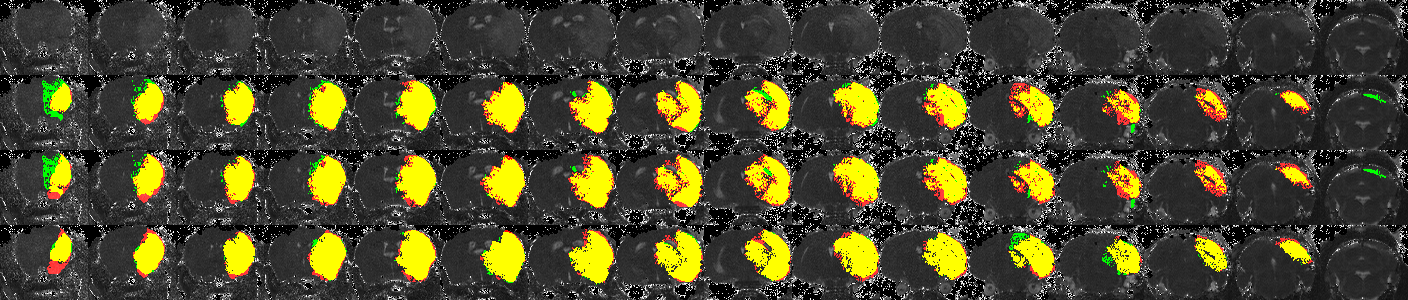

Supplement: Supplementary Material 1 — Segmentation results on the entire validation set. [file DataSheet1.zip › LS_m22_24h_segmentation_results.tif]

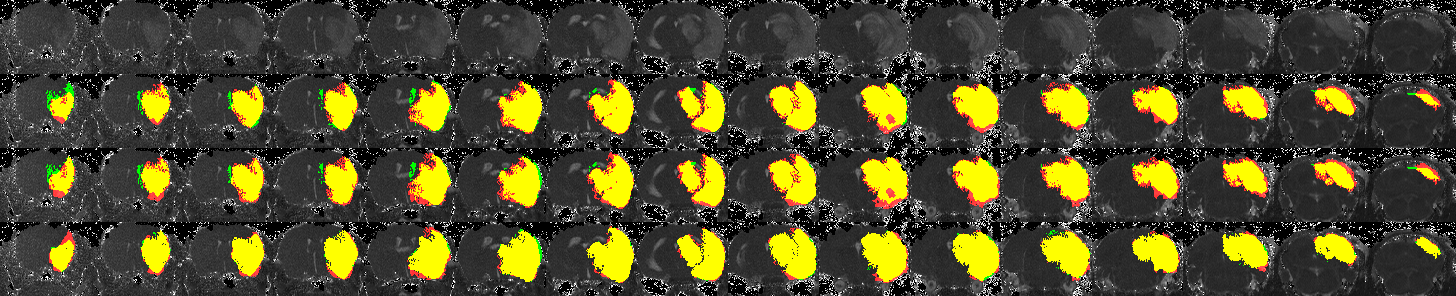

Supplement: Supplementary Material 1 — Segmentation results on the entire validation set. [file DataSheet1.zip › LS_m23_24h_segmentation_results.tif]

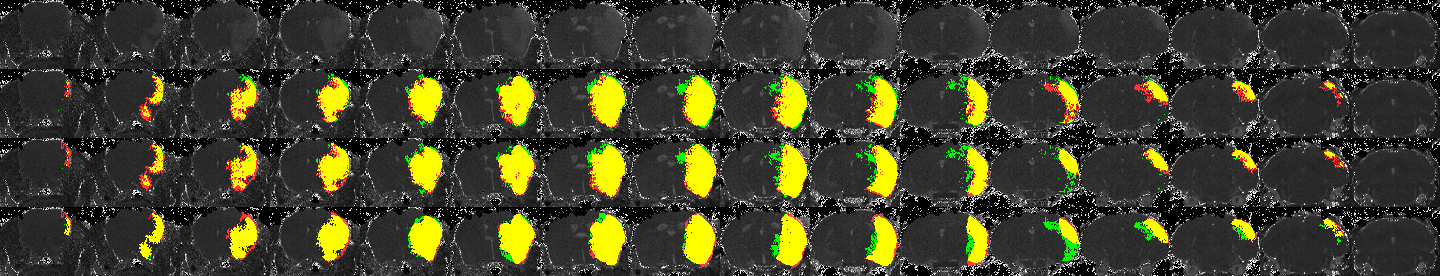

Supplement: Supplementary Material 1 — Segmentation results on the entire validation set. [file DataSheet1.zip › LS_m24_24h_segmentation_results.tif]

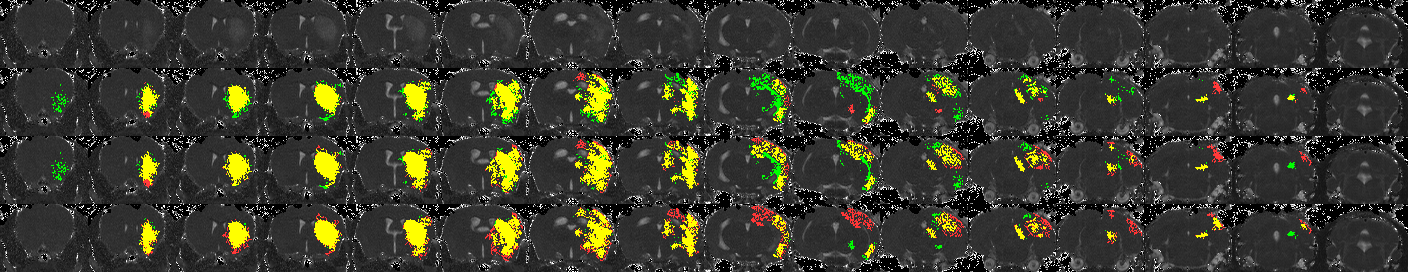

Supplement: Supplementary Material 1 — Segmentation results on the entire validation set. [file DataSheet1.zip › LS_m25_24h_segmentation_results.tif]

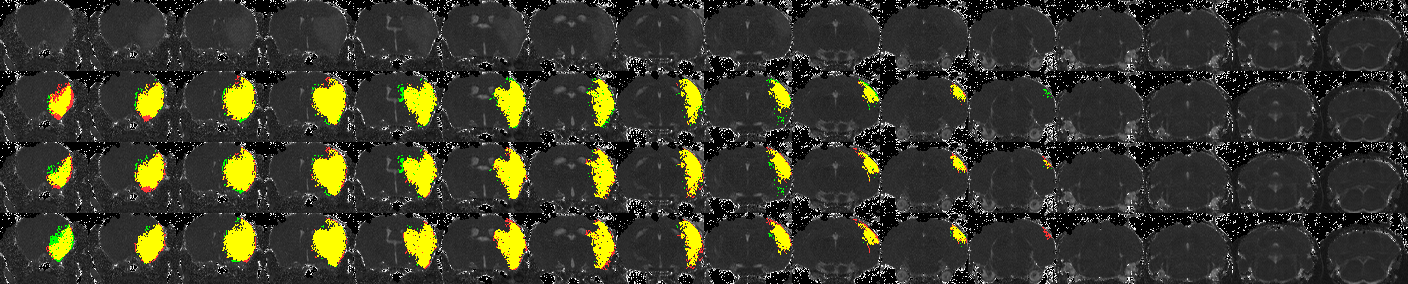

Supplement: Supplementary Material 1 — Segmentation results on the entire validation set. [file DataSheet1.zip › LS_m26_24h_segmentation_results.tif]

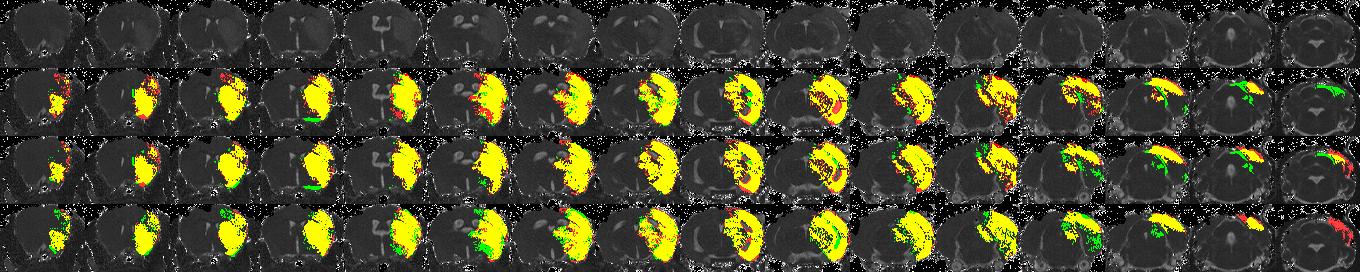

Supplement: Supplementary Material 1 — Segmentation results on the entire validation set. [file DataSheet1.zip › LS_m27_24h_segmentation_results.tif]

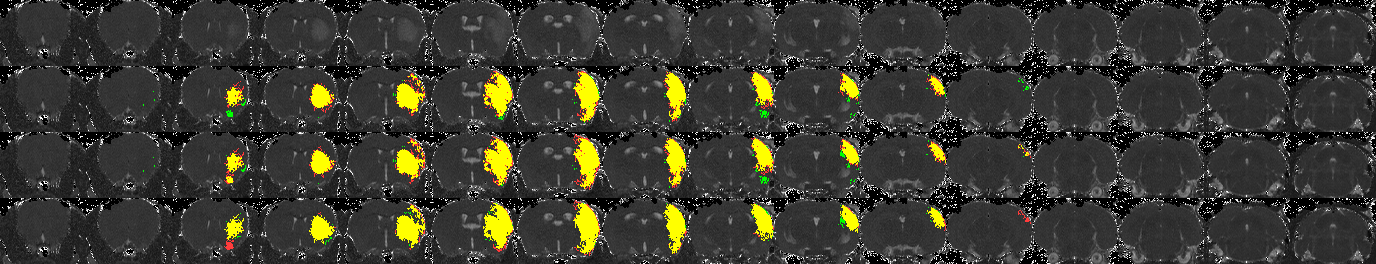

Supplement: Supplementary Material 1 — Segmentation results on the entire validation set. [file DataSheet1.zip › LS_m28_24h_segmentation_results.tif]

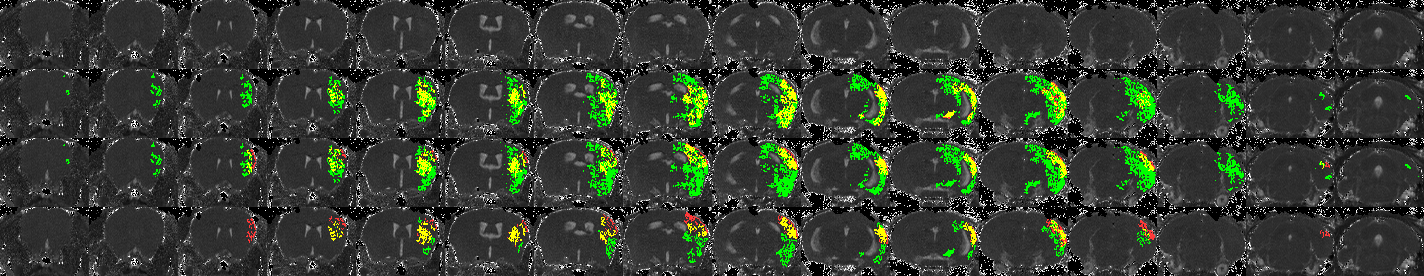

Supplement: Supplementary Material 1 — Segmentation results on the entire validation set. [file DataSheet1.zip › LS_m29_8d_segmentation_results.tif]

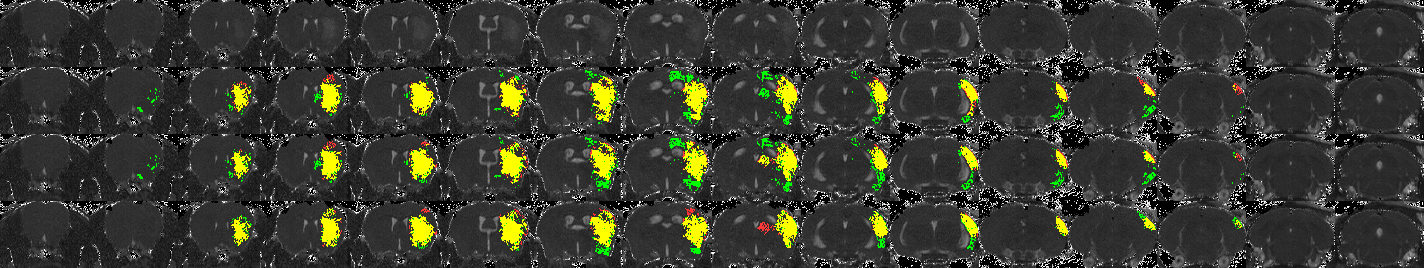

Supplement: Supplementary Material 1 — Segmentation results on the entire validation set. [file DataSheet1.zip › LS_m29_24h_segmentation_results.tif]

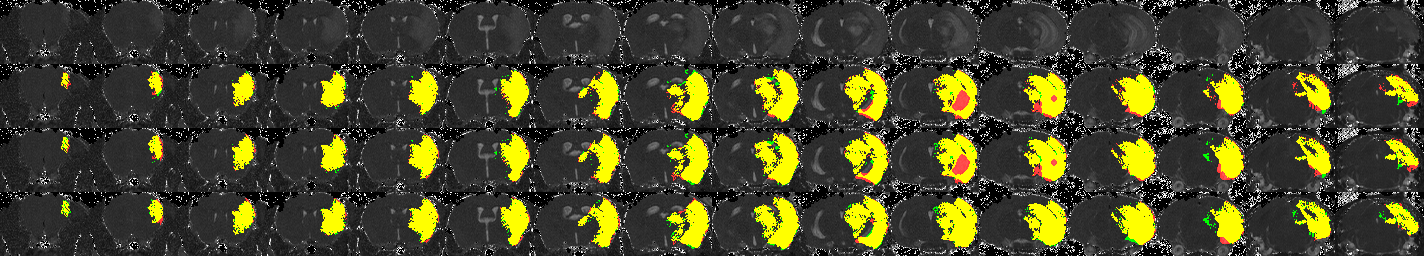

Supplement: Supplementary Material 1 — Segmentation results on the entire validation set. [file DataSheet1.zip › LS_m30_24h_segmentation_results.tif]

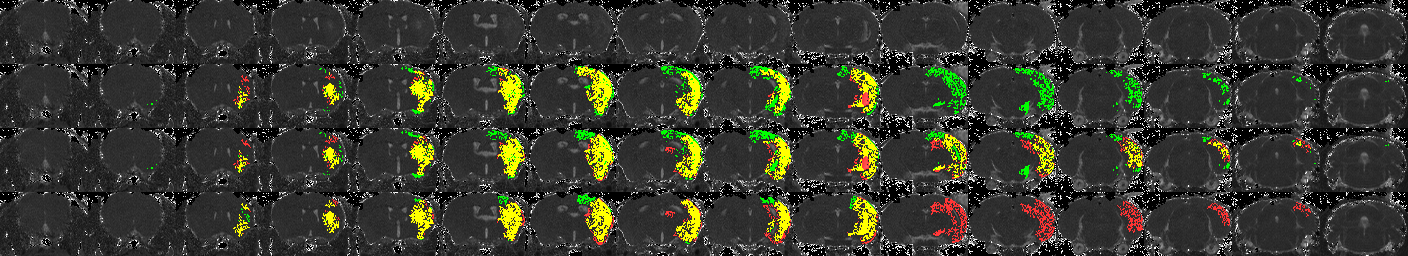

Supplement: Supplementary Material 1 — Segmentation results on the entire validation set. [file DataSheet1.zip › LS_m31_8d_segmentation_results.tif]

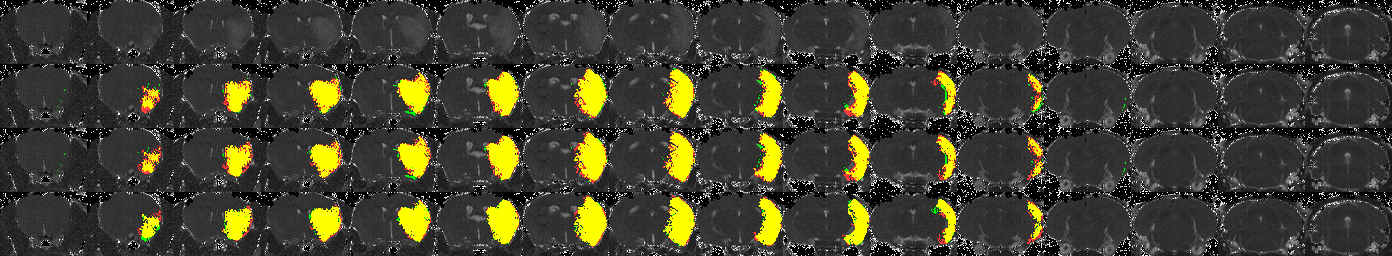

Supplement: Supplementary Material 1 — Segmentation results on the entire validation set. [file DataSheet1.zip › LS_m31_24h_segmentation_results.tif]

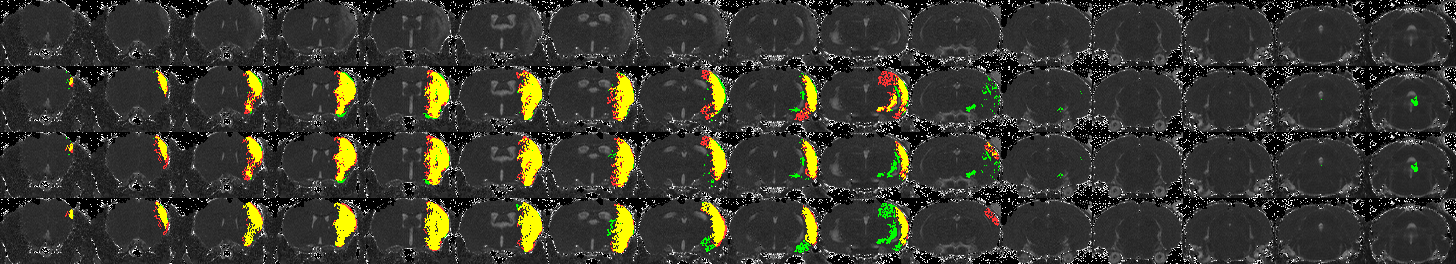

Supplement: Supplementary Material 1 — Segmentation results on the entire validation set. [file DataSheet1.zip › LS_m32_8d_segmentation_results.tif]

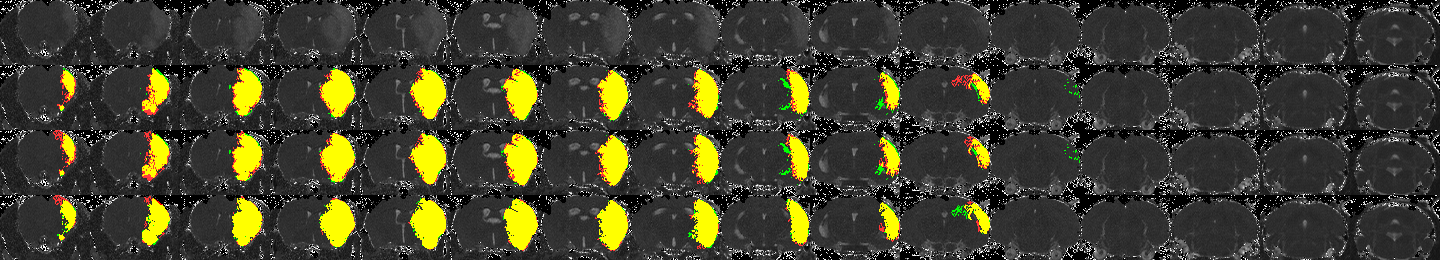

Supplement: Supplementary Material 1 — Segmentation results on the entire validation set. [file DataSheet1.zip › LS_m32_24h_segmentation_results.tif]

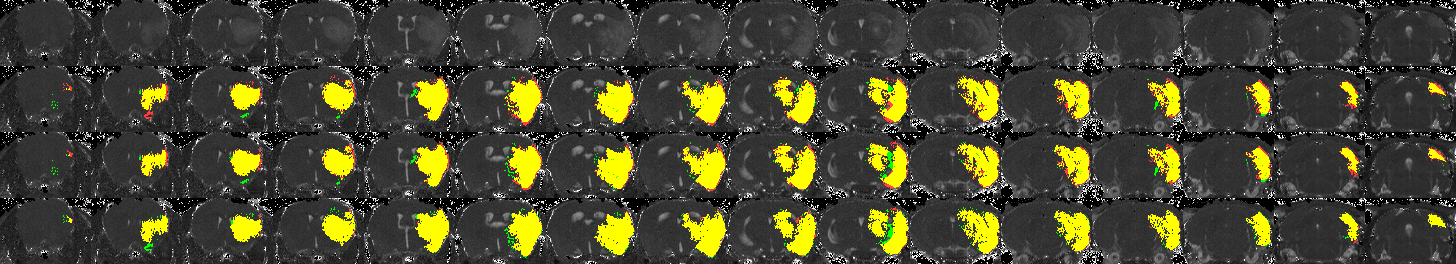

Supplement: Supplementary Material 1 — Segmentation results on the entire validation set. [file DataSheet1.zip › LS_m33_24h_segmentation_results.tif]

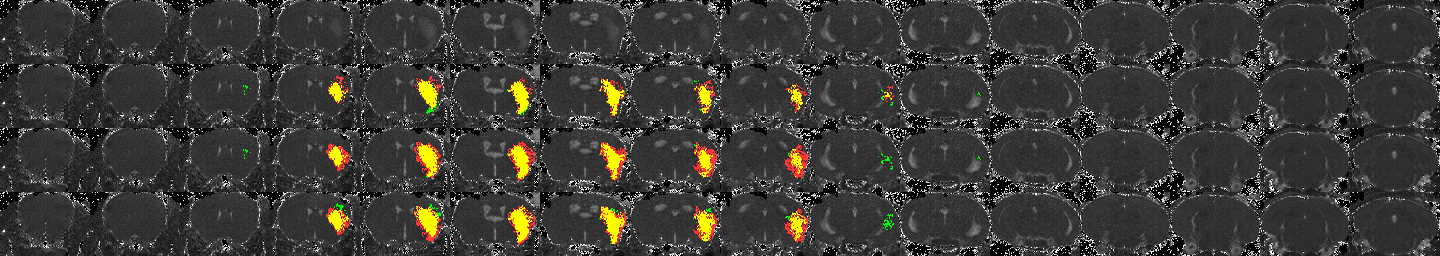

Supplement: Supplementary Material 1 — Segmentation results on the entire validation set. [file DataSheet1.zip › LS_m34_24h_segmentation_results.tif]

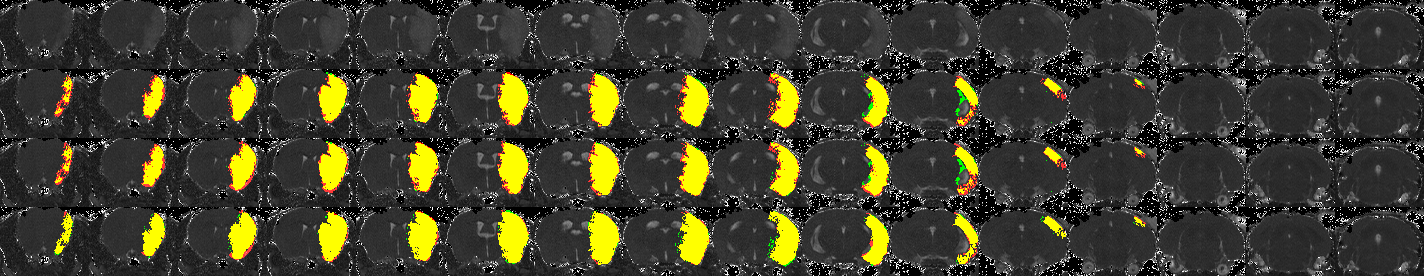

Supplement: Supplementary Material 1 — Segmentation results on the entire validation set. [file DataSheet1.zip › LS_m35_24h_segmentation_results.tif]

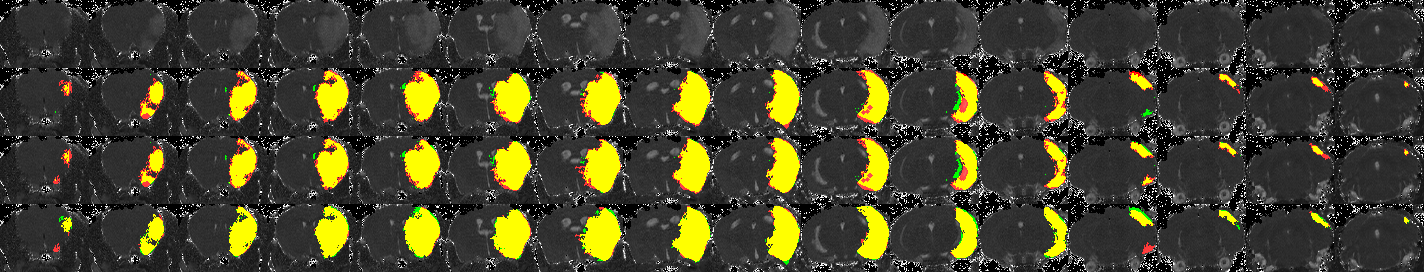

Supplement: Supplementary Material 1 — Segmentation results on the entire validation set. [file DataSheet1.zip › LS_m36_24h_segmentation_results.tif]

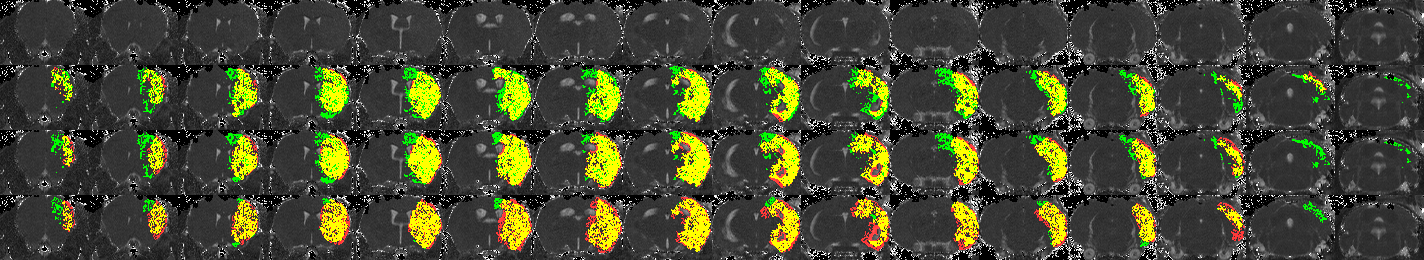

Supplement: Supplementary Material 1 — Segmentation results on the entire validation set. [file DataSheet1.zip › LS_m37_4h_segmentation_results.tif]

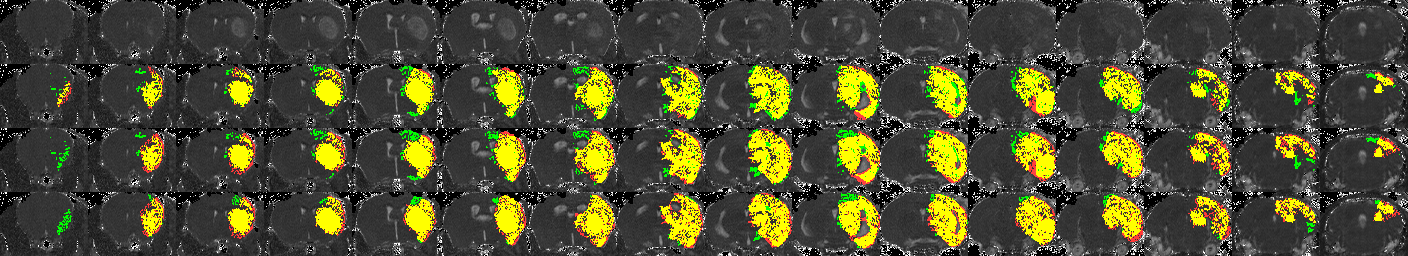

Supplement: Supplementary Material 1 — Segmentation results on the entire validation set. [file DataSheet1.zip › LS_m38_4h_segmentation_results.tif]

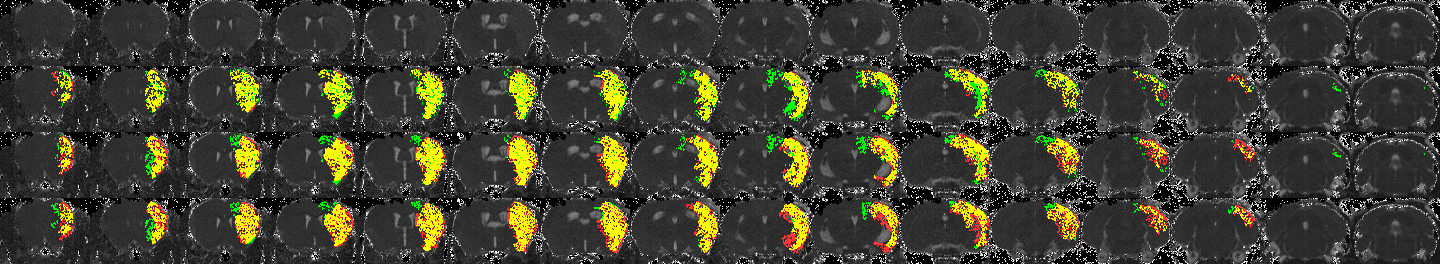

Supplement: Supplementary Material 1 — Segmentation results on the entire validation set. [file DataSheet1.zip › LS_m39_4h_segmentation_results.tif]

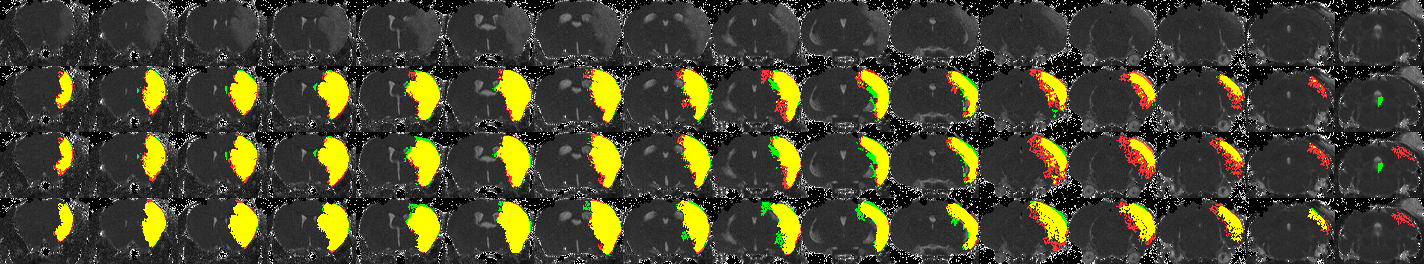

Supplement: Supplementary Material 1 — Segmentation results on the entire validation set. [file DataSheet1.zip › LS_m39_24h_segmentation_results.tif]

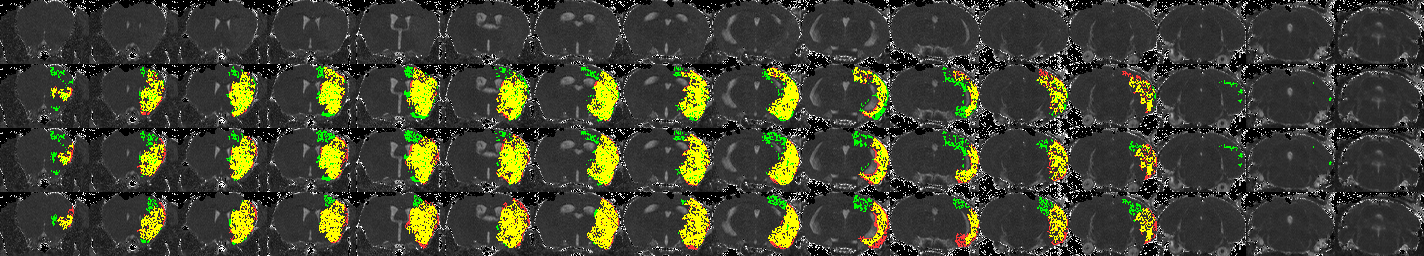

Supplement: Supplementary Material 1 — Segmentation results on the entire validation set. [file DataSheet1.zip › LS_m40_4h_segmentation_results.tif]

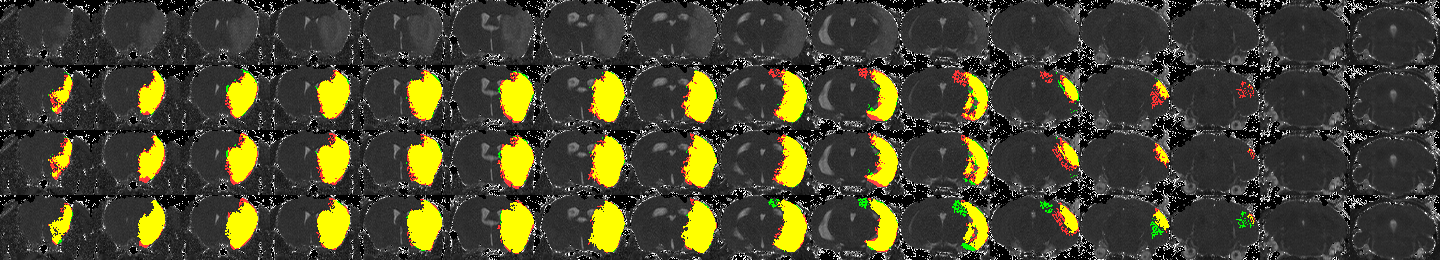

Supplement: Supplementary Material 1 — Segmentation results on the entire validation set. [file DataSheet1.zip › LS_m40_24h_segmentation_results.tif]

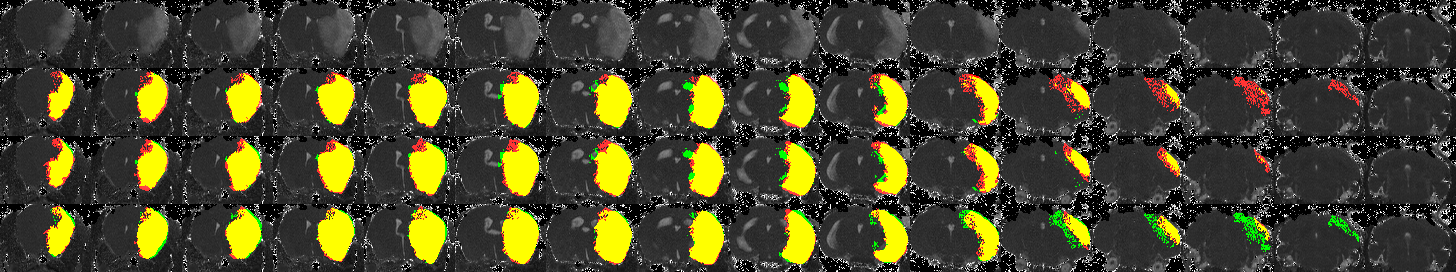

Supplement: Supplementary Material 1 — Segmentation results on the entire validation set. [file DataSheet1.zip › LS_m40_48h_segmentation_results.tif]

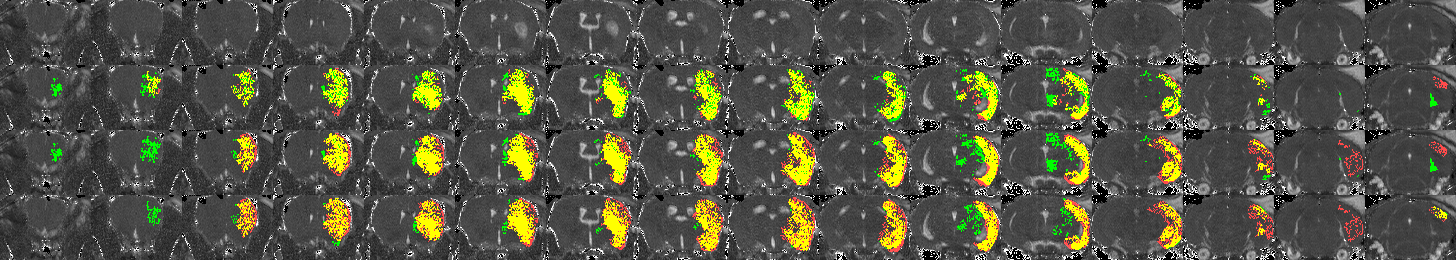

Supplement: Supplementary Material 1 — Segmentation results on the entire validation set. [file DataSheet1.zip › LS_m41_4h_segmentation_results.tif]

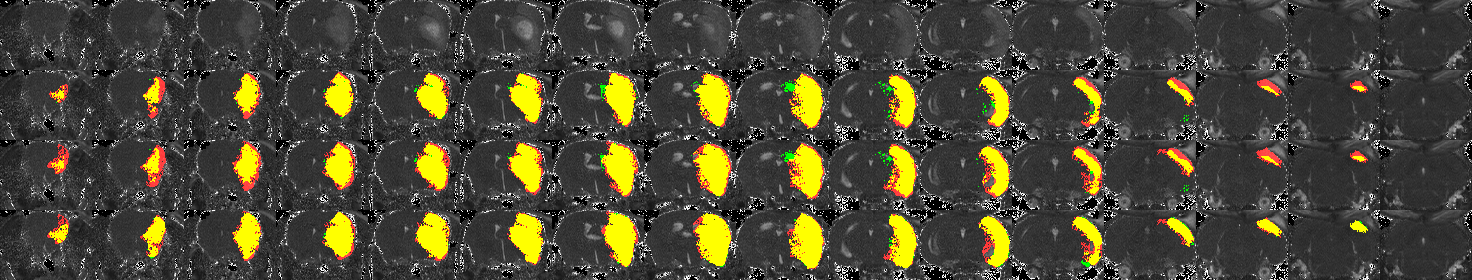

Supplement: Supplementary Material 1 — Segmentation results on the entire validation set. [file DataSheet1.zip › LS_m41_24h_segmentation_results.tif]

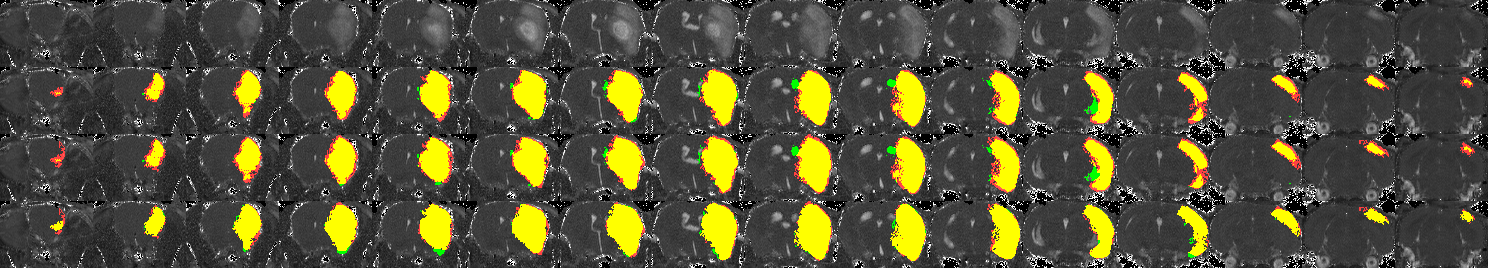

Supplement: Supplementary Material 1 — Segmentation results on the entire validation set. [file DataSheet1.zip › LS_m41_48h_segmentation_results.tif]

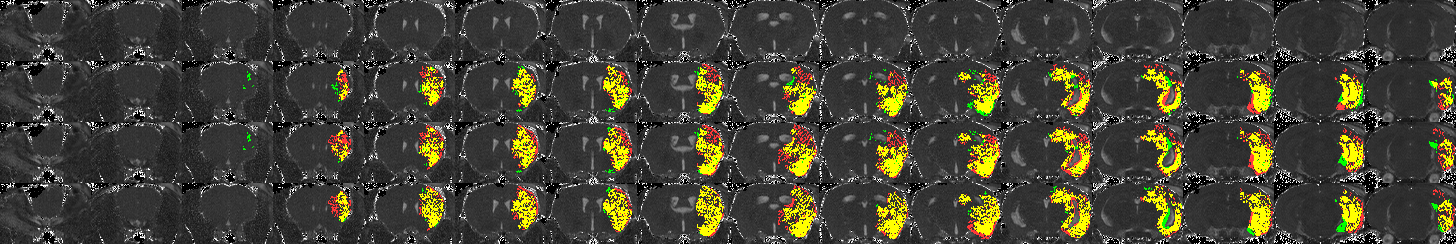

Supplement: Supplementary Material 1 — Segmentation results on the entire validation set. [file DataSheet1.zip › LS_m42_4h_segmentation_results.tif]

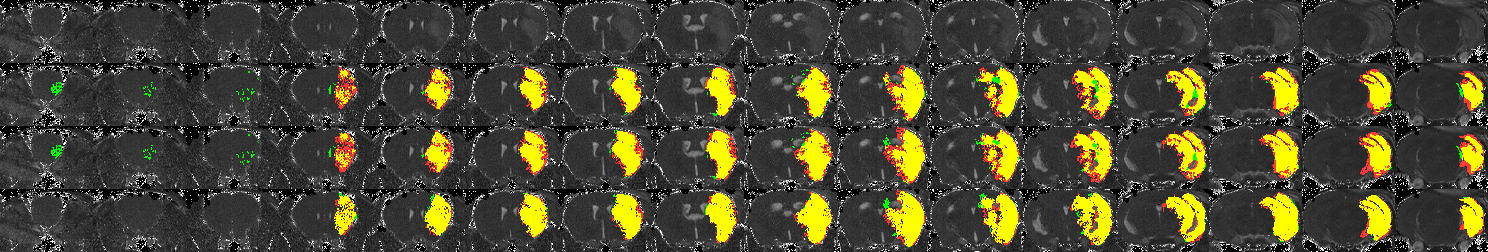

Supplement: Supplementary Material 1 — Segmentation results on the entire validation set. [file DataSheet1.zip › LS_m42_24h_segmentation_results.tif]

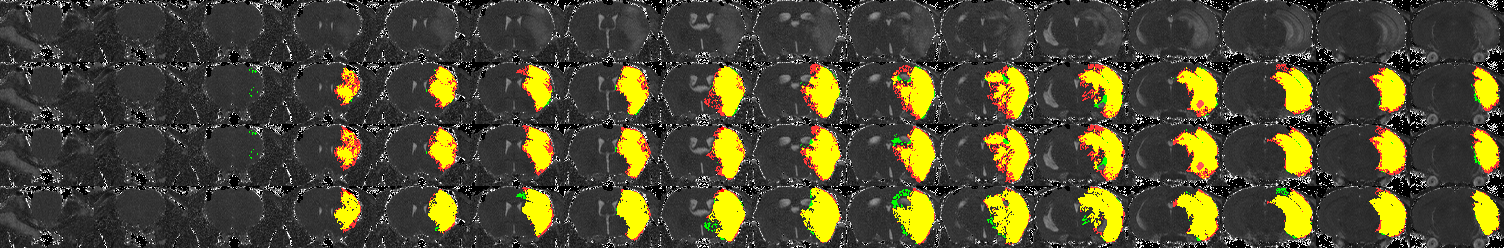

Supplement: Supplementary Material 1 — Segmentation results on the entire validation set. [file DataSheet1.zip › LS_m42_48h_segmentation_results.tif]

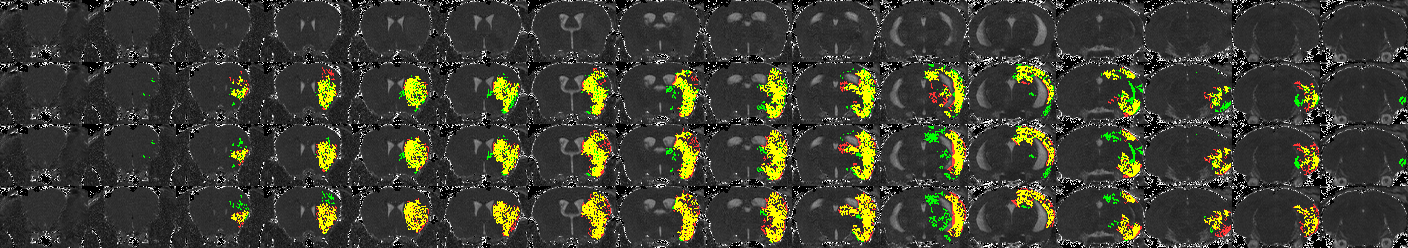

Supplement: Supplementary Material 1 — Segmentation results on the entire validation set. [file DataSheet1.zip › LS_m43_4h_segmentation_results.tif]

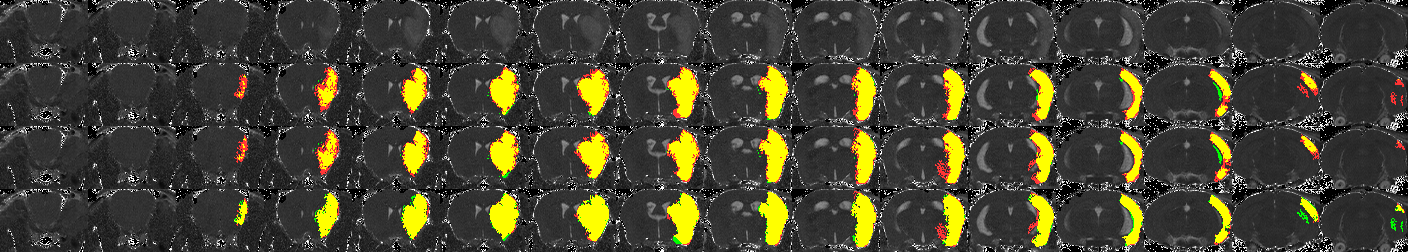

Supplement: Supplementary Material 1 — Segmentation results on the entire validation set. [file DataSheet1.zip › LS_m43_24h_segmentation_results.tif]

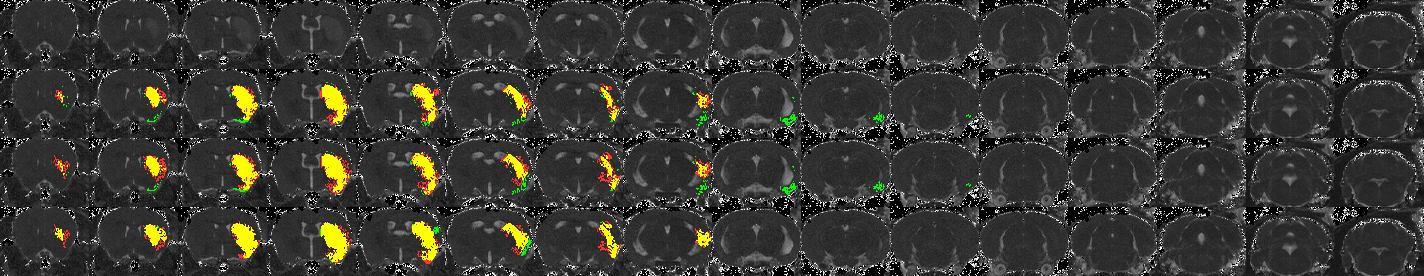

Supplement: Supplementary Material 1 — Segmentation results on the entire validation set. [file DataSheet1.zip › LS_m44_24h_segmentation_results.tif]

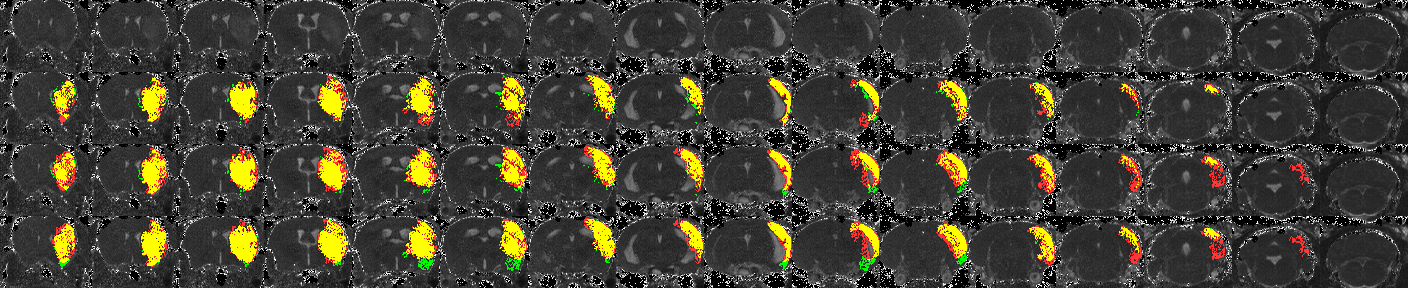

Supplement: Supplementary Material 1 — Segmentation results on the entire validation set. [file DataSheet1.zip › LS_m44_48h_segmentation_results.tif]

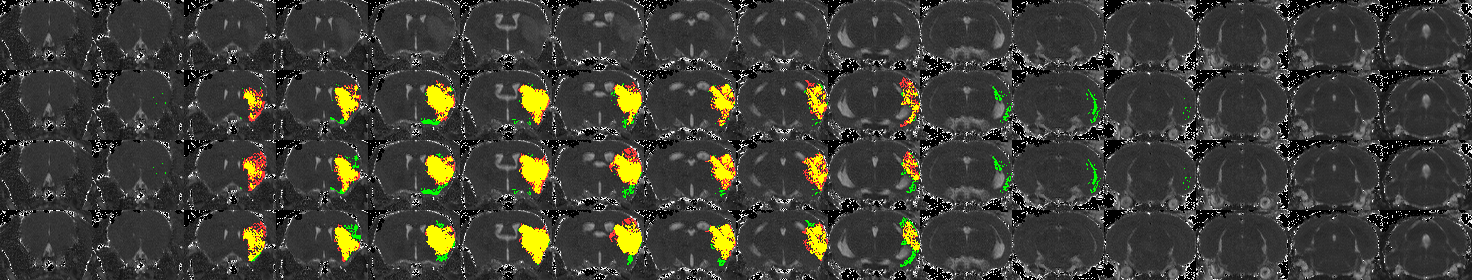

Supplement: Supplementary Material 1 — Segmentation results on the entire validation set. [file DataSheet1.zip › LS_m45_24h_segmentation_results.tif]

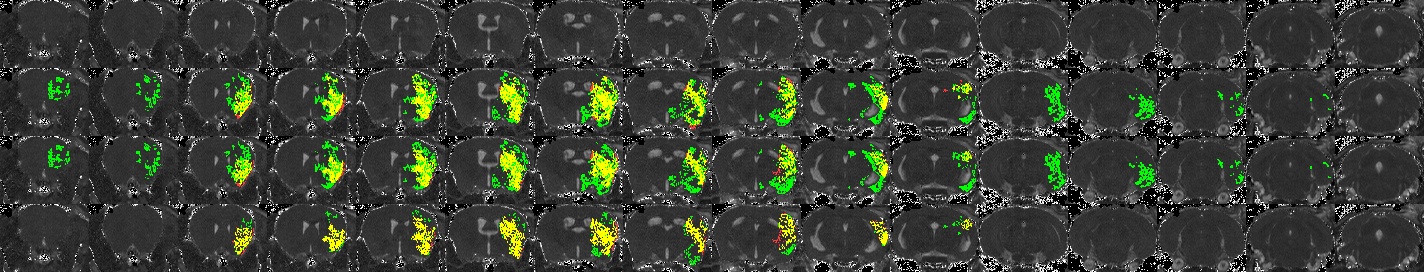

Supplement: Supplementary Material 1 — Segmentation results on the entire validation set. [file DataSheet1.zip › LS_m46_4h_segmentation_results.tif]

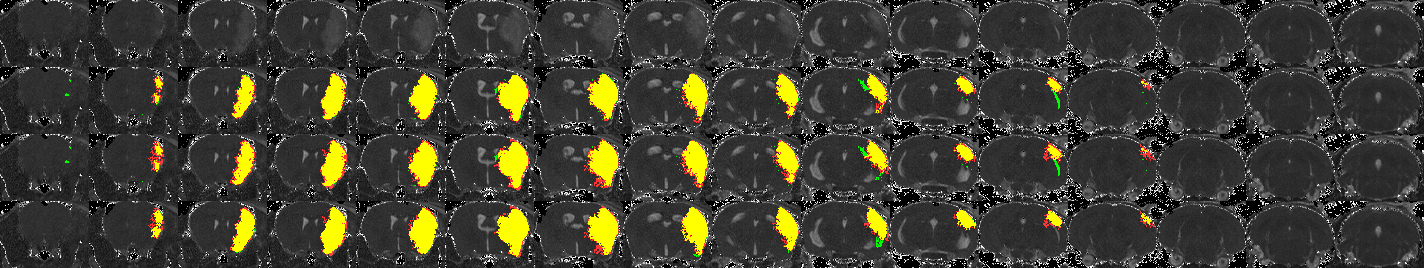

Supplement: Supplementary Material 1 — Segmentation results on the entire validation set. [file DataSheet1.zip › LS_m46_24h_segmentation_results.tif]

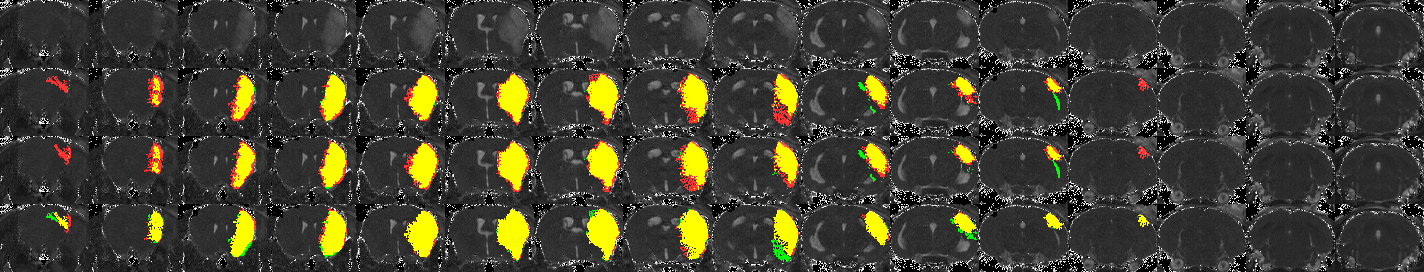

Supplement: Supplementary Material 1 — Segmentation results on the entire validation set. [file DataSheet1.zip › LS_m46_48h_segmentation_results.tif]

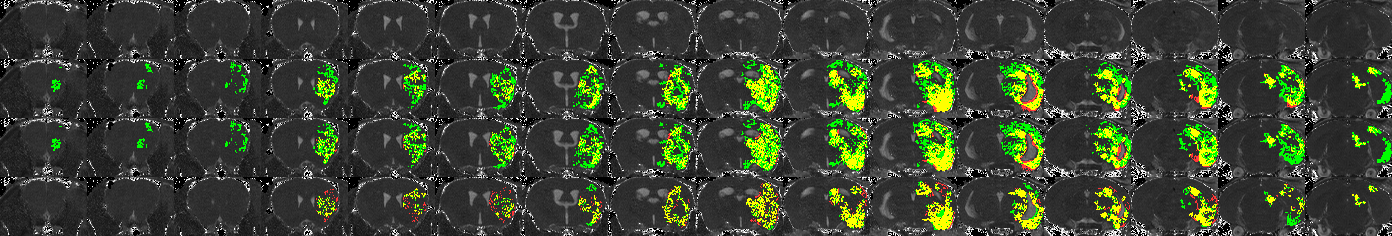

Supplement: Supplementary Material 1 — Segmentation results on the entire validation set. [file DataSheet1.zip › LS_m47_4h_segmentation_results.tif]

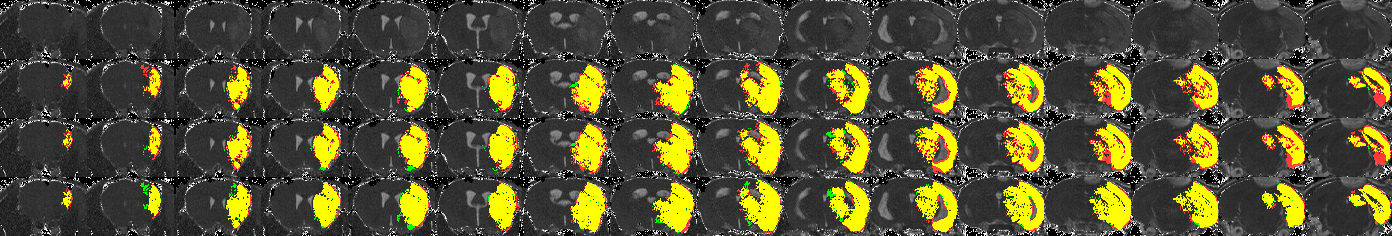

Supplement: Supplementary Material 1 — Segmentation results on the entire validation set. [file DataSheet1.zip › LS_m47_24h_segmentation_results.tif]

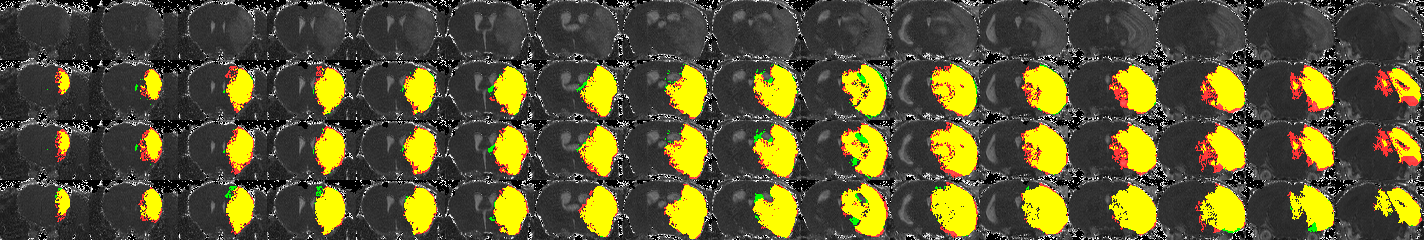

Supplement: Supplementary Material 1 — Segmentation results on the entire validation set. [file DataSheet1.zip › LS_m47_48h_segmentation_results.tif]

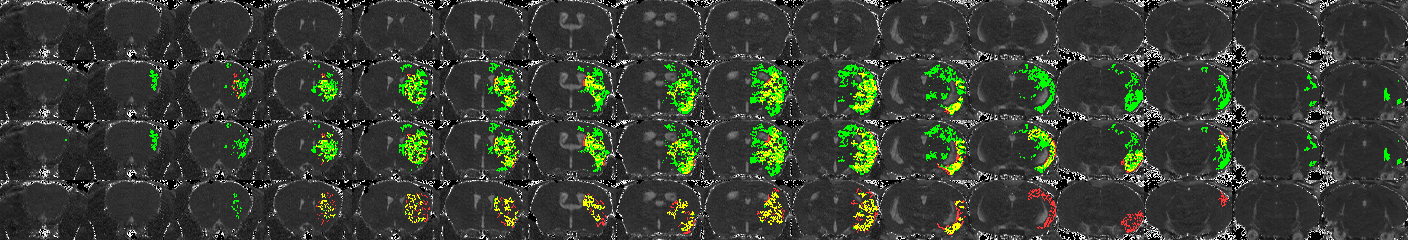

Supplement: Supplementary Material 1 — Segmentation results on the entire validation set. [file DataSheet1.zip › LS_m48_4h_segmentation_results.tif]

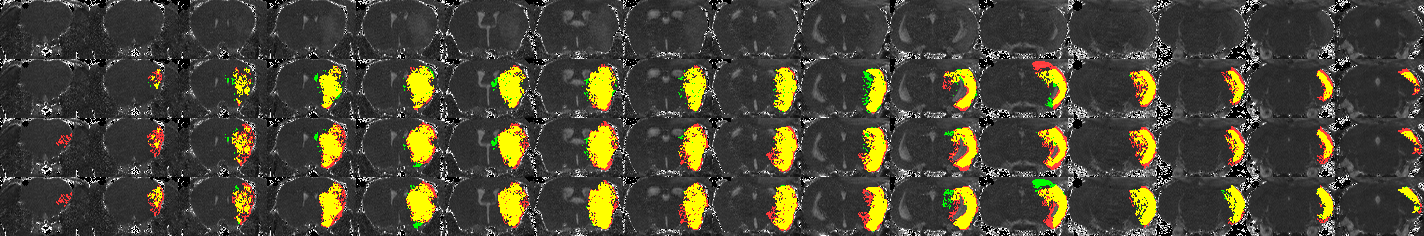

Supplement: Supplementary Material 1 — Segmentation results on the entire validation set. [file DataSheet1.zip › LS_m48_24h_segmentation_results.tif]

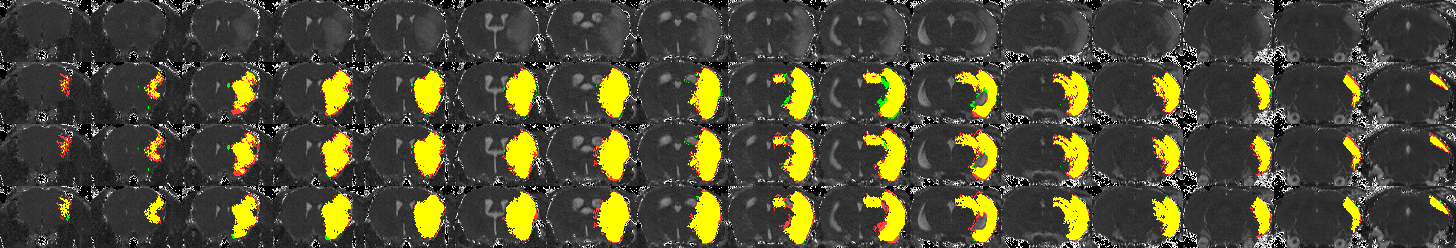

Supplement: Supplementary Material 1 — Segmentation results on the entire validation set. [file DataSheet1.zip › LS_m48_48h_segmentation_results.tif]
